# Haptotropic Phenomena in Digold(I) Triple-Bonded Complexes

**DOI:** 10.1021/acs.inorgchem.5c04642

**Published:** 2025-12-05

**Authors:** Ignacio Nieto-Vargas, Juan Cayuela-Castillo, Francisco J. Fernández-de-Córdova, Israel Fernández, Pablo Ríos

**Affiliations:** † Instituto de Investigaciones Químicas (IIQ), Departamento de Química Inorgánica, Centro de Innovación en Química Avanzada (ORFEO−CINQA), 201454 CSIC and Universidad de Sevilla, 41092 Sevilla, Spain; ‡ Departamento de Química Orgánica I and Centro de Innovación en Química Avanzada (ORFEO−CINQA), Facultad de Químicas, 16734 Universidad Complutense de Madrid, Madrid 28040, Spain

## Abstract

Addition of either IPrAuOTf or IPrCuOTf (IPr = 1,3-bis­(2,6-diisopropylphenyl)­imidazol-2-ylidene; OTf = trifluoromethanesulfonate anion) to the digold acetylide IPrAuC≡CAuIPr results in the selective formation of the corresponding trimetallic cationic species [IPrAuC≡C­(π-MIPr)­AuIPr]­[OTf] (M = Au or Cu). Variable-temperature ^1^H NMR experiments (VT-NMR) reveal that while the homotrimetallic gold complex exhibits dynamic σ,π-exchange in solution at temperatures even as low as −130 °C, the heterometallic analogue presents a static scenario. On the other hand, extension of the acetylide bridge by one additional acetylide unit using IPrAuC≡C–C≡CAuIPr introduces a new fluxional process in the corresponding analogous trimetallic compounds [IPrAuC≡C­(π-MIPr)–C≡CAuIPr]­[OTf] (M = Au or Cu), namely π,π-exchange. In the case of the copper-containing complex, this exchange occurs even at low temperatures, whereas exchange can be thermally arrested in the trigold system at temperatures below −10 °C. Computational studies indicate that the divergent behavior between gold and copper regarding π,π-exchange does not appear to stem from their interaction with the alkyne fragment but rather in how this interaction changes along the reaction coordinate toward the transition state geometry.

## Graphical abstract

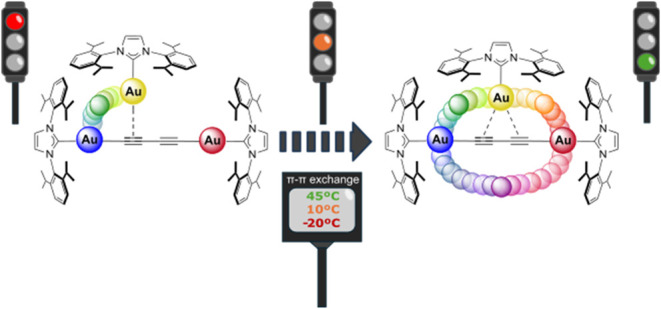

## Introduction

Historically, the field of homogeneous gold­(I) catalysis has been primarily centered on the activation of C–C multiple bonds, typically including a single metal center in the catalytic cycle.^1^ However, a paradigm shift took place in 2008 when Houk, Toste, and co-workers evidenced that cationic digold­(I) σ,π-acetylide complexes are key reactive intermediates in the catalytic cycloisomerization of 1,5-allenynes.^2^ This initial discovery spurred the investigation of cationic diaurated species and their role in the catalytic activation of π systems by others. Consequently, this collective effort has dramatically expanded the scope of synthetic strategies that leverage gold in organic chemistry.^3^ Nevertheless, there is still ongoing debate regarding the role of cationic digold­(I) compounds stemming from the unpredictable reactivity of these species. While some groups have reported examples wherein digold­(I) σ,π-acetylide complexes are productive intermediates in catalytic transformations,^2,4^ others have demonstrated that digold­(I) σ,π-acetylide and/or *gem*-diaurated complexes are off-cycle, dead-ends in catalysis.^4g,5^ This unpredictability makes catalyst selection non-obvious and thus hampers rational reaction design.

Moreover, despite the numerous examples of organogold complexes, information on their fluxional phenomena remains limited, which is striking, given that many gold-catalyzed reactions involve thermodynamic equilibria. In the particular case of cationic digold­(I) σ,π-acetylides, some examples include equilibria between mono- and diaurated species,^5b^ between alkenes and alkynes,^6^ competition between π or σ,π activation modes,^7^ or equilibria involving coordination of the LAu^+^ moiety to different alkyne fragments within the same molecule (Scheme 1A).^4,8^ Results from the computational investigations of the latter example suggest that the formation of dibenzopentalenes through intramolecular cyclization is possible because an NHC–Au^+^ (NHC =*N*-heterocyclic carbene) group is able to migrate from a thermodynamically stable σ,π geometry wherein both gold atoms are on the same π system (Isomer **I**, Scheme 1A) to a higher energy isomer **II**, which enables the subsequent steps.^8^ Although intermediates like **II** have been previously proposed,^4^ this case clearly illustrates how the mobility of the NHC–Au^+^ is key in the catalytic process, as observed for a similar rearrangement where the computationally proposed mobility of gold across an aryne intermediate determines the observed product (Scheme 1B).^4c^ In addition to the positionally fluxional bonding of each NHC-Au^+^ fragment between different alkyne moieties, the coordination mode between these fragments can also display dynamic behavior, as the triple bond can undergo “slippage” between η^2^ and η^1^ geometries, with the latter mode exhibiting increased electrophilicity of the distal carbon atom (Scheme 1C).^6,9^

**Scheme 1:**
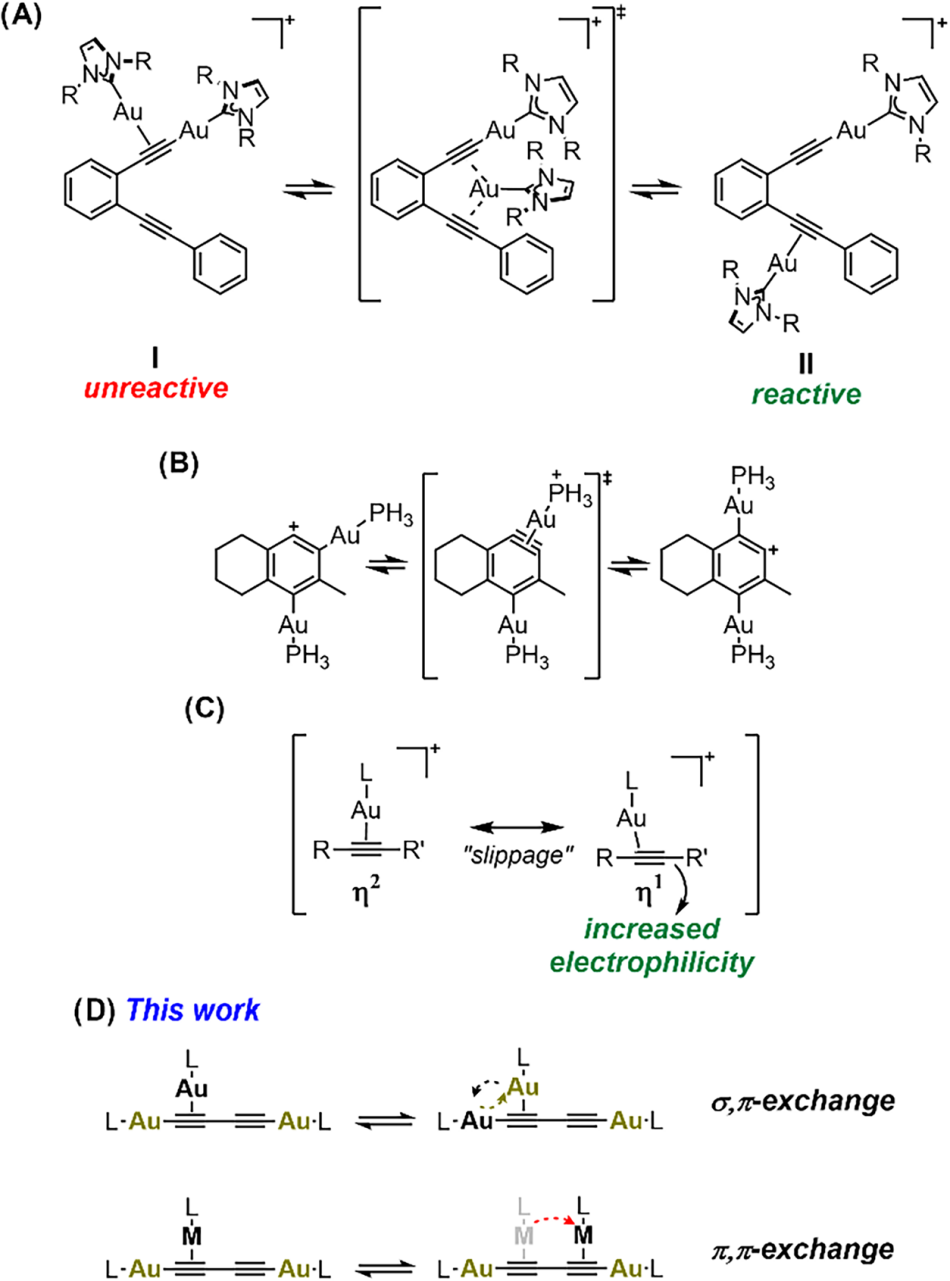
(A–C) Representative Examples of Computationally Investigated Fluxional Processes Involving Cationic Au­(I) Alkyne Species and (D) Dynamic Phenomena Explored in this Work

While fluxionality has been identified computationally as a widespread, critical process in gold­(I) acetylide complexes, to the best of our knowledge, in-depth experimental studies where certain dynamic processes can be thermally arrested are nonexistent, leaving a gap that is essential to investigate for a deeper understanding of the factors that control the electronic properties of these species, and ultimately, their reactivity. For this purpose, we describe in this work the preparation of digold­(I) complexes with a variable number of acetylide units and their coordination to IPr–M^+^ (M = Au, Cu) fragments to yield the corresponding trimetallic species. These products exhibit σ,π, and/or π,π haptotropic shifts (Scheme 1D), which have been examined in detail both experimentally and computationally.

## Results and Discussion

Previous research from this laboratory concerning migratory insertion reactions on NHC-supported bimetallic gold­(I) acetylide complexes involved the synthesis of trinuclear species **1** (Scheme 2 and Figure 1) as part of our mechanistic studies.^10^ Cationic trimetallic compound **1** comprises three IPrAu units surrounding an acetylide C_2_^2–^ moiety charge-balanced with a triflate anion. In the solid state, two of the IPrAu fragments are σ-bound to the acetylide carbon atoms, while the third metal is π-bound to the C≡C triple bond. However, compound **1** displays dynamic behavior in solution, as only one set of IPr resonances (as opposed to two sets in a 1:2 ratio) is observed by ^1^H NMR spectroscopy in THF-*d*_8_ at temperatures as low as −95 °C.^10^ This observation suggests that a rapid σ,π haptotropic shift occurs, making all of the IPrAu groups chemically equivalent, in line with previously reported σ,π-digold­(I) acetylide examples.^4d,5c,11^ Thus, in an attempt to arrest this exchange, we carried out VT-NMR experiments over an expanded temperature range. However, slow exchange could not be observed even at −130 °C using CDCl_2_F as solvent (Figure S11),^12^ indicating that the exchange between the σ and π coordination modes is extremely facile in this complex. Density functional theory (DFT) calculations corroborate this observation, as the calculated Gibbs activation barrier for this process is only 5.3 kcal mol^–1^ (see SI for full computational details).^13^ In order to examine how the metal identity may influence the fluxionality of the resulting complex, a heterometallic analogue was prepared. To this end, 1 equiv of IPrCuOTf was added to a solution of bimetallic acetylide **2** in dichloromethane at 25 °C (Scheme 2); precipitation by addition of *n*-pentane, followed by cooling at –30 °C yielded species **3** in 73% yield.^14^

**Figure 1:**
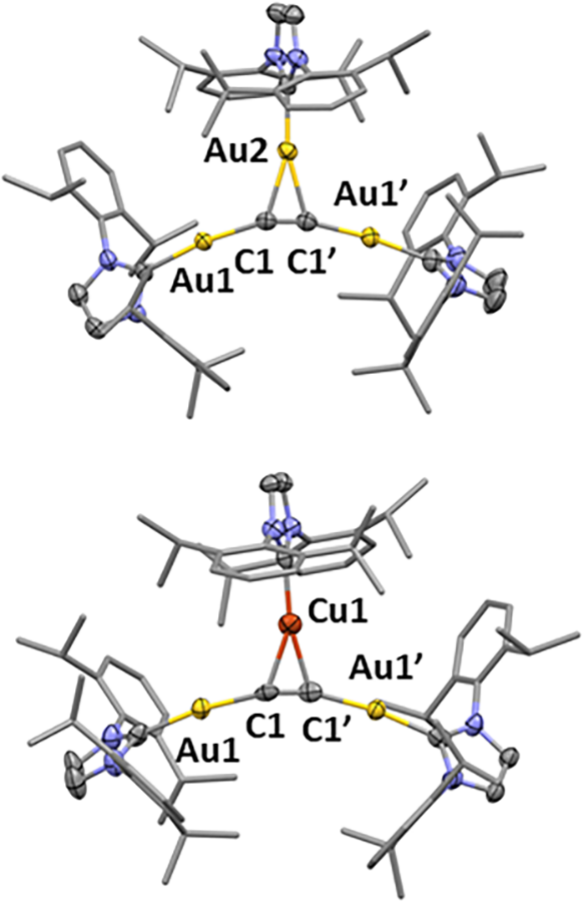
Solid-state structure of trimetallic complexes **1** and **3** (50% probability ellipsoids). H atoms, THF molecules, and OTf anions were omitted, and 2,6-diisopropylphenyl groups were represented as capped sticks for clarity. Partial occupation of Au in the position of Cu1 in complex **3** is not shown.

**Scheme 2:**
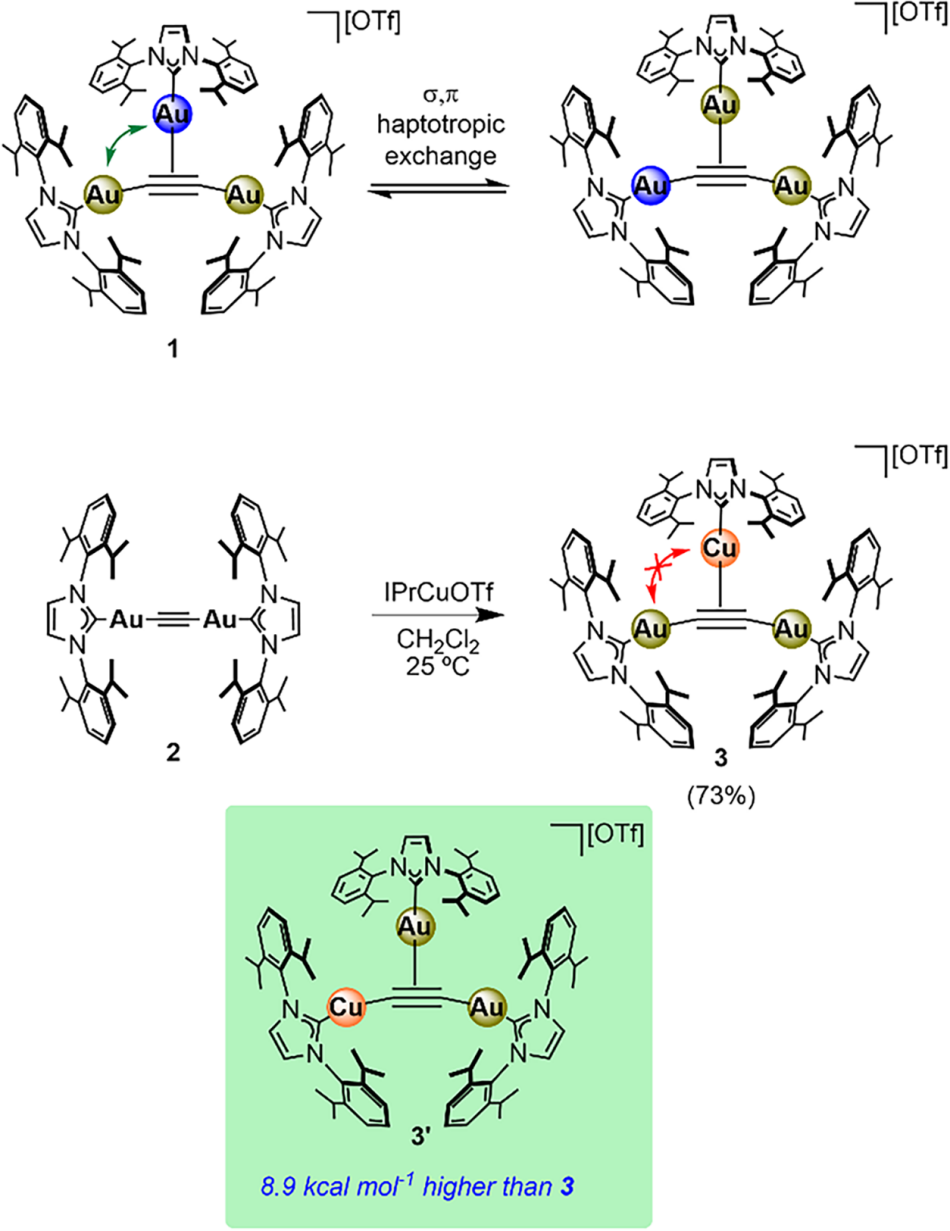
Metal Dependence of σ,π-Exchange in Complexes **1** and **3**

Structural assignment of trimetallic complex **3** was confirmed by X-ray diffraction studies on single crystals grown by slow diffusion of *n-*pentane into a THF solution of **3** (Figure 1). As with **1**, the IPrCu^+^ unit of **3** establishes π-bonding with the acetylide moiety in a symmetrical manner (Cu–C1 and Cu–C1′ bond distances ≈ 2.1 Å) with no appreciable metallophilic interactions, given that the Cu···Au distances of 3.5 Å are considerably longer than the sum of their van der Waals radii (3.06 Å).^15^ For comparison, similar features are observed for trigold complex **1**, which also presents symmetrical π-bonding (Au–C bond distances ≈ 2.2 Å) and no aurophilic contacts (Au···Au ≈ 3.6–3.7 Å).^10,16^ Both solid-state structures exhibit bending of the acetylide Au–C≡C–Au axis (∠Au1–C1–C1′ angles ≈ 161–166°) likely due to the steric congestion attributed to the IPr ligands. Surprisingly, partial scrambling of complex **3** appears to occur from thermal decomposition in solution during the synthesis through an unidentified mechanism. This is evidenced by the cocrystallization of complex **1** within the crystal structure of target compound **3**, based on the 71:29 Cu/Au occupancy at the central metal site in the asymmetric unit (Figure S33). Consistently, ^1^H NMR spectroscopy presents small amounts (ca. 10%) of complex **1** in the spectrum of species **3**. However, the ^1^H NMR analysis of **3** at 25 °C reveals two sets of resonances for the IPr ligands in a 2:1 ratio (Figure S1), in agreement with a static structure wherein the IPrCu^+^ component does not exchange with the σ-bound IPrAu units under these conditions (Scheme 2). Additionally, heating a solution of **3** in 1,2-dichlorobenzene at temperatures up to 125 °C did not appreciably alter the ^1^H NMR spectrum (Figure S12). These results highlight the stark contrast between the two complexes, as simply changing the central metal atom (despite both being group-11 metals) dramatically affects the fluxional behavior of the system. This seems to be related to the heterometallic nature of the system, given that rapid σ,π exchange has been previously observed for dicopper­(I) and tetracopper­(I) acetylides.^17^ Although the putative isomer **3′** may also be expected to give rise to the observed NMR data (i.e., σ-bound IPrCu^+^ and two rapidly exchanging IPrAu moieties, Scheme 2), it is 8.9 kcal mol^–1^ higher in energy than complex **3** (according to DFT calculations; the transition state for the σ,π exchange could not be located). These findings are consistent with the infeasibility of the σ,π exchange process between Cu and Au under the experimental conditions tested.

Motivated by these results, we hypothesized that extending the acetylide linker should increase the number of fluxional processes displayed by these platforms since the presence of two C≡C bonds could potentially lead to π,π haptotropic shifts in addition to the σ,π shifts previously described. Metal π-π exchange has been described in the literature as walking^18^ or sliding^19^ and is of paramount importance in areas such as molecular switches,^20^ the selective activation of C–X bonds,^21^ and polymerization processes (catalyst-transfer polycondensation).^22^ However, dynamic processes involving π,π exchanges on gold­(I) complexes remain unknown.

The extension of the acetylide linker was effected through the synthetic strategy depicted in Scheme 3. Heating of a mixture of trimethylsiloxide complex **4** ^23^ and 0.5 equiv of 1,4-bis­(trimethylsilyl)­butadiyne in toluene solution at 120 °C for 4 days led to the selective formation of the target digold­(I) diacetylide **5** and hexamethyldisiloxane (HMDSO), as observed by ^1^H NMR spectroscopy. Species **5** was isolated in 87% yield as an analytically pure beige solid, which readily crystallized from toluene/*n*-pentane mixtures, allowing for its solid-state structure determination via single-crystal X-ray diffraction (Figure 2). The most noticeable feature of its geometry is the slightly bent character of the Au–C≡C–C≡C–Au axis (∠Au1–C1–C2 angle = 170.4(6)°), presumably due to packing effects, given that longer *sp* carbon chains are usually necessary for curved polyyne structures.^24^ Similar to acetylide **2**, compound **5** reacts with 1 equiv of either IPrAuOTf or IPrCuOTf to yield the corresponding trinuclear architectures **6** and **7**, respectively, in excellent yields (Scheme 3). The solid-state structures of these complexes were obtained from the X-ray diffraction analysis of single crystals grown by the vapor diffusion of *n*-pentane into THF solutions of each compound (Figure 2). Both structures present the third IPrM^+^ fragment bound to one of the C≡C bonds of the diacetylide, causing a deviation of the adjacent σ-bound IPrAu moiety away from the linearity with the rest of the C≡C–C≡C–Au axis due to the steric demand of the IPr ligands. While the third gold­(I) atom binds an alkyne fragment symmetrically in species **6**, the structure of complex **7** evinces partial slippage of the Cu atom (Figure 2), contrary to some examples in the literature.^6^ The addition of 1 equiv of IPrAuOTf to compound **6** in an attempt to install a fourth IPrM^+^ group did not result in any change in the ^1^H NMR spectrum. Presumably, the addition of another fragment in this manner is inhibited by the significant steric occlusion about the diyne unit observed in both structures.

**Figure 2:**
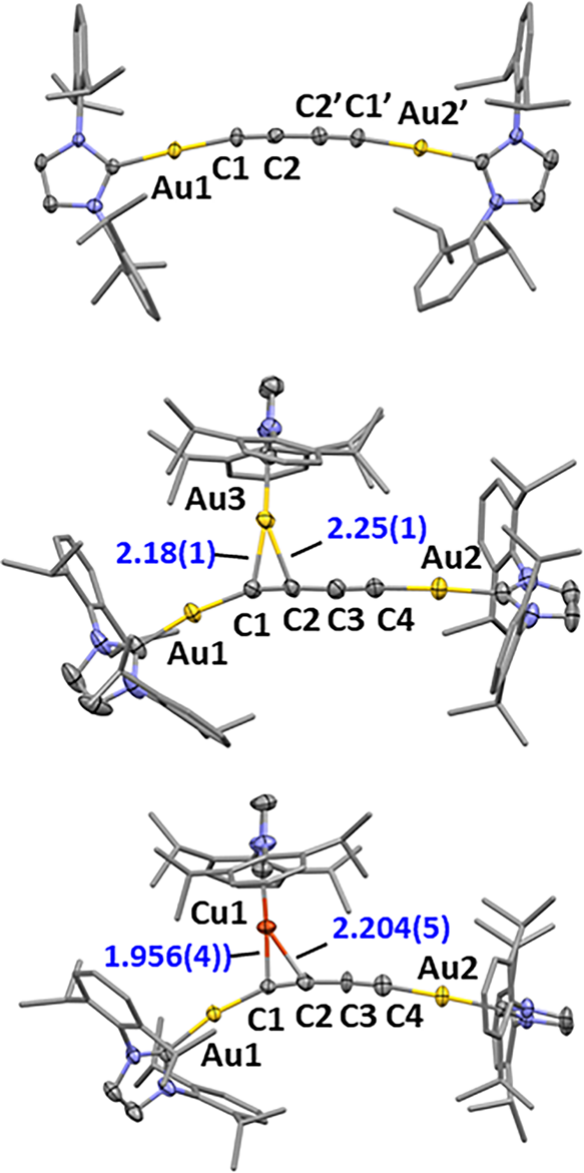
Solid-state structure of complexes **5**, **6**, and **7** (50% probability ellipsoids). H atoms and OTf anions were omitted, and 2,6-diisopropylphenyl groups were represented as capped sticks for the sake of clarity. Only one component of the unit cell of **7** is shown (the other one presents the IPrCu^+^ fragment approximately perpendicular to that observed in the figure, see Figure S33). Selected bond distances are given in angstroms.

**Scheme 3:**
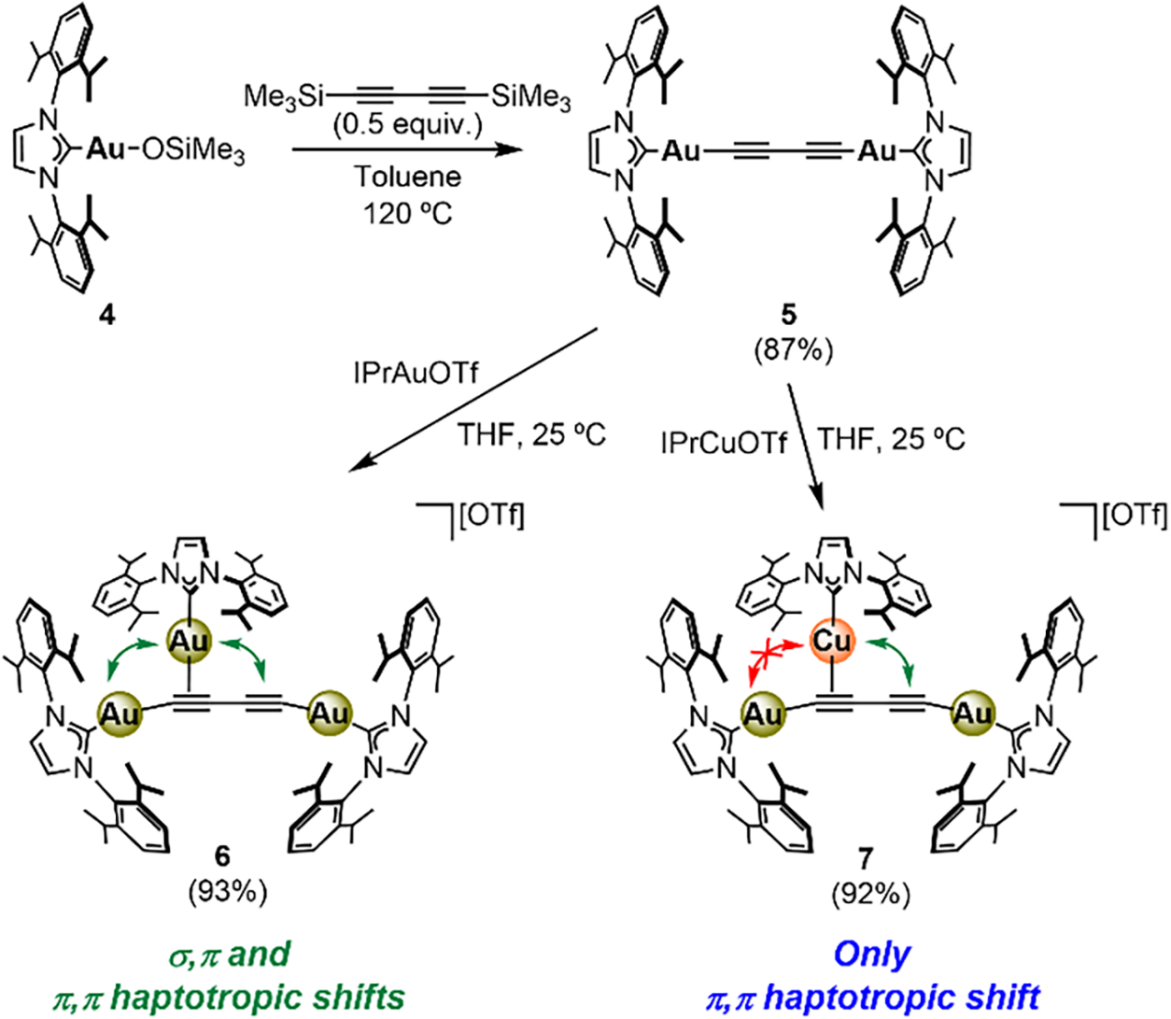
Preparation of Complexes **5**-**7** (Isolated Yields in Parentheses)

In order to investigate the solution state behavior of these π-extended complexes, we employed VT-NMR spectroscopic techniques. The ^1^H NMR spectrum of trigold­(I) complex **6** in CDCl_3_ at 25 °C displays coalescence of the IPrAu resonances (Figure S14). Indeed, warming the sample to 55 °C reveals one single set of well-resolved signals for the IPr ligands, consistent with complex **6** exhibiting rapid σ,π and π,π-haptotropic shifts at these temperatures. Conversely, cooling the sample to −10 °C results in the splitting of resonances into two sets of IPr signals with a 2:1 ratio, in agreement with a bonding situation where only the π,π-haptotropic shift has ceased and the σ,π-exchange is still operative (Figure 3). This observation has also been corroborated via NOESY experiments (Figures S22–S24). The ^1^H NMR spectrum does not further evolve at lower temperatures (−90 °C in CD_2_Cl_2_ or −130 °C in CDCl_2_F), consistent with the dynamic behavior of **1**. In order to rule out alkyne displacement by the triflate anion (as previously reported by Widenhoefer et al.),^11c^ **6·BAr**_**24**_^**F**^ was prepared (BAr_24_^F^ = tetrakis­[3,5-bis­(trifluoromethyl)­phenyl]­borate group, see SI). Nonetheless, similar spectra and activation parameters were observed (see Figure S28), ruling out the potential alkyne displacement by the weakly coordinating anion.

**Figure 3:**
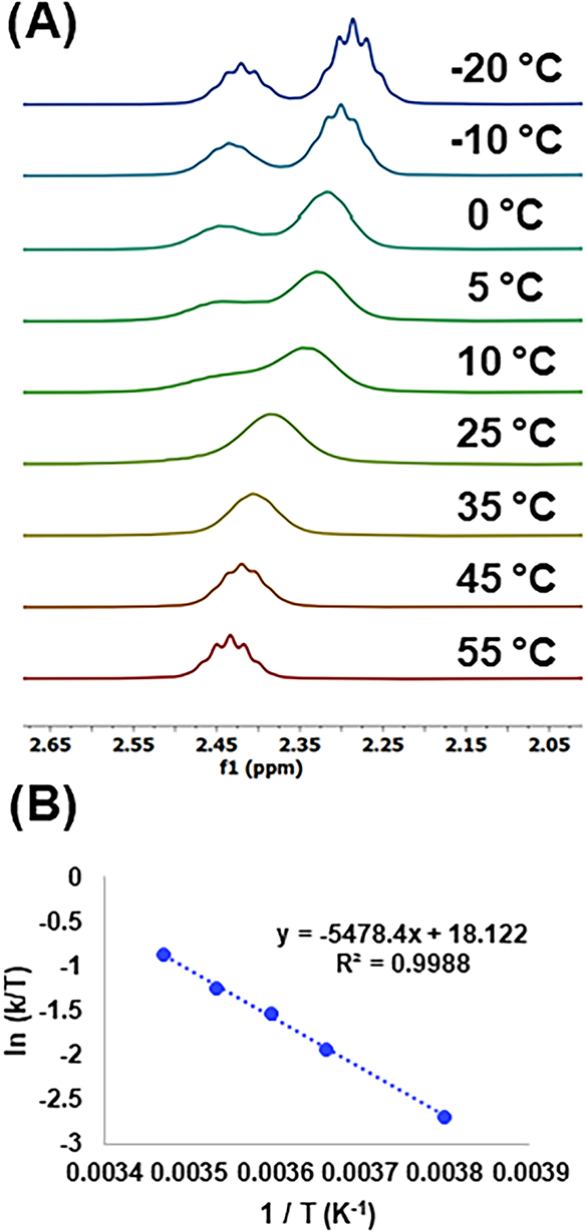
(A) Portion of the variable-temperature ^1^H NMR spectra (400 MHz, CDCl_3_) of complex **6**, corresponding to the methine groups of the IPr ligands. (B) Eyring plot for the π,π-haptotropic shift in complex **6** (ln (k/T) vs 1/T).

Exchange rate constants for the π,π-shift process were extracted from the ^1^H NMR spectra (see SI for details),^25^ from which an Eyring analysis afforded an activation enthalpy Δ*H* ^‡^ = 10.9 ± 0.2 kcal mol^–1^ and an activation entropy Δ*S* ^‡^ = −11.2 ± 0.8 cal mol^–1^ K^–1^ (Figure 3). Thus, the Gibbs free energy associated with this process is Δ*G* ^‡^ = 14.2 ± 0.2 kcal mol^–1^. Similar parameters were obtained using methods based on the coalescence temperature (Table S1).^26^ DFT calculations are in good agreement with these results, giving Δ*G* ^‡^ = 15.7 kcal mol^–1^, Δ*H* ^‡^ = 13.9 kcal mol^–1^, and Δ*S* ^‡^ = −6.2 cal mol^–1^ K^–1^.

In contrast to that of **6**, the ^1^H NMR spectrum of heterometallic complex **7** in CD_2_Cl_2_ at 25 °C exhibits two sets of resonances for the IPr ligands in a 2:1 ratio. Lowering the temperature in CDCl_2_F to −120 °C did not result in any appreciable change in the spectrum that would be consistent with the deceleration of dynamic phenomena. This observation, in combination with the information gathered above for **3** and **6**, points to a facile π,π-haptotropic shift of the IPrCu^+^ unit along the two triple bonds and the absence of σ,π exchange. Again, transition states and structures derived from potential Au–Cu σ,π exchange in **7** are energetically unfavorable (Figure S41). Warming of compound **7** in 1,2-dichlorobenzene to 105 °C led to the progressive broadening of the IPrCu^+^ resonances while the diacetylide-bound IPrAu peaks remained unaltered, indicating potential decoordination of IPrCu^+^ from the π system rather than haptotropic phenomena. As expected, returning to 25 °C gave an identical ^1^H NMR spectrum to the starting one (Figure S21). In contrast, the VT-NMR experiment of **3** did not reveal the broadening of any of the resonances during heating, suggesting a tighter binding between IPrCu^+^ and the triple bond. The computed barrier for the π,π-exchange of the copper­(I) fragment in **7** is also consistent with the experimental observations, as Δ*G* ^‡^ = 9.2 kcal mol^–1^, Δ*H* ^‡^ = 7.2 kcal mol^–1^, and Δ*S* ^‡^ = −6.5 cal mol^–1^ K^–1^. Therefore, Cu­(I) seems to present less difficulty to hop between the two alkyne groups compared to Au­(I), and it does not appear to engage in σ,π exchange with gold under the aforementioned experimental conditions.

The more facile metallotropy observed for Cu­(I)-containing complex **7** as compared to the analogous Au­(I)-only complex **6** can be initially attributed to a weaker interaction between the Cu­(I)-NHC moiety and the digold­(I) diacetylide **5**, as found in related alkyne complexes.^27^ However, energy decomposition analysis-natural orbital for chemical valence (EDA-NOCV) calculations indicate that the intrinsic interaction (Δ*E*_int_) between **5** and [IPrCu]^+^ in complex **7** is nearly identical to that involving [IPrAu]^+^ in complex **6** (see Table 1). At variance, this interaction is much stronger in the transition state associated with the π-π shift involving the Cu­(I)-system (**TS-7**). As a result, the change in the interaction energy in going from the initial complex to the corresponding transition state (ΔΔ*E*_int_) is much more pronounced in the Au­(I)-only complex than in its Cu­(I)-containing counterpart, i.e., ΔΔ*E*_int_ = 13.7 vs 6.4 kcal mol^–1^, respectively, which matches the total energy difference between the initial species and the corresponding transition structures (Δ*E* = 12.9 vs 6.5 kcal mol^–1^), therefore constituting the main factor controlling the different barrier heights in the metallotropic transformation. Partitioning of the crucial Δ*E*_int_ term into its constituent energy contributors indicates that the lower ΔΔ*E*_int_ computed for the Cu­(I)-containing system does not originate from the change in the Pauli repulsion (ΔΔ*E*_Pauli_ = −15.9 vs −39.0 kcal mol^–1^ for the Cu­(I)-containing and Au­(I)-only species, respectively) but from smaller differences in electrostatic interactions (ΔΔ*E*_elstat_ = 19.9 vs 42.1 kcal mol^–1^) and, to a lesser extent, also from smaller differences in orbital interactions (ΔΔ*E*_orb_ = 4.0 vs 12.0 kcal mol^–1^, respectively), including both the σ-donation (ρ1) and the π-backdonation (ρ2). The smaller differences in electrostatic interactions have also been supported via molecular electrostatic potential (MESP) distribution and charge analysis (see Figure S42).^28^ These results therefore suggest that the lower barrier observed for the π-π metallotropy involving **7** is not dominated by the interaction between the individual fragments in the initial complexes but by the change of these interactions as the transition states are reached.

**Table 1:** EDA-NOCV Values (in kcal/mol) for Complexes **6** and **7** and Their Corresponding Transition States **TS-6** and **TS-7** for the π,π-Haptotropic Shift^a^

	**6**	**TS-6**	**ΔΔE**	**7**	**TS-7**	**ΔΔE**
Δ*E*‡			12.9			6.5
Δ*E*_int_	–107.7	–94.0	13.7	–107.6	–101.2	6.4
Δ*E*_Pauli_	139.4	100.4	-39.0	92.9	76.9	-15.9
Δ*E*_elstat_	–146.0	–103.9	42.1	–111.7	–91.8	19.9
Δ*E*_orb_	–77.4	–65.4	12.0	–65.0	–61.0	4.0
Δ*E*_orb_(ρ1)	–33.4	–24.5	8.9	–24.6	–18.1	6.5
Δ*E*_orb_(ρ2)	–12.3	–6.9	5.4	–9.1	–7.1	2.0
Δ*E*_disp_	–23.7	–25.0	**-1.3**	–23.7	–25.2	-1.5

a All data have been computed at the ZORA-PBE(0)-D3BJ/DZP//SMD-PBE(0)-D3BJ/6-31G­(d,p)&SDD (Cu,Au)

## Concluding Remarks

In conclusion, this work describes the preparation of group-11-based trimetallic mono- and diacetylide complexes and the investigation of their dynamic behavior in solution. While σ,π-exchange phenomena in gold-only complexes **1** and **6** seem to be extremely facile even at −130 °C, the copper-containing complexes of **3** and **7** do not appear to exhibit σ,π shifts under the experimental conditions tested. On the other hand, π,π metallotropic shifts are observed in the synthesized diacetylide species for both gold and copper-containing units. Whereas the π,π-shift of copper in compound **7** cannot be thermally arrested at low temperatures, this process can be halted in trigold complex **6** at −10 °C. The stark difference between both complexes is computationally supported to reside in the higher electrostatic and orbital energy changes necessary for complex **6** to achieve the transition state geometry. These results demonstrate how the nature of the metal has a pronounced repercussion in the resulting fluxional character of a certain chemical system, which can be of interest from the point of view of molecular machines. Additionally, these findings provide the first experimental evidence for a dynamic process in which the gold moiety can be thermally “localized” on one region of the alkynyl-containing molecule, as it is no longer able to alternate between the triple bonds. These findings might have implications from the perspective of homogeneous catalysis, as thermal control and/or metal identity can drive the selectivity of a certain transformation.^29^ On the other hand, the mobility of NHC–M^+^ fragments along a carbon chain can be regarded as a molecular model of NHC-gold adatoms or adsorbed metals on surfaces,^30^ whose motion is critical in the formation of self-assembled monolayers (SAMs).

## Experimental Details

Unless stated otherwise, all reactions were performed in a glovebox or on a Schlenk line under an atmosphere of pure Ar or high-purity N_2_ by using standard Schlenk techniques. All solvents were dried and degassed prior to use. C_6_D_6_ was distilled under Ar and stored over 3 Å molecular sieves for at least 24 h prior to use. CDCl_3_, CD_2_Cl_2_, and 1,2-dichlorobenzene were dried over calcium hydride before being distilled and degassed by three freeze–pump–thaw cycles. IPrAuOTf,^29^ IPrCuOTf,^31^ CDCl_2_F,^12^ and complexes **1** ^10^ and **4** ^23^ were prepared according to literature procedures. All other reagents were purchased from commercial suppliers and used as received. CDCl_2_F was distilled and stored over 3 Å molecular sieves in a sealed vessel at −24 °C. No uncommon hazards are noted for the experimental work described in this article.^32^

## Supplementary Material





## Notes

Computed species (XYZ)
Material including synthetic, spectroscopic, crystallographic, and computational details (PDF)
