# Supplementary material for: Haptotropic Phenomena in Digold(I) Triple-Bonded Complexes
Source: Inorg Chem. 2025 Dec 5;65(13):7153–60. doi: 10.1021/acs.inorgchem.5c04642 (PMC13058889; doi:10.1021/acs.inorgchem.5c04642)
Supplement: Supplementary file 2 [file ic5c04642_si_002.pdf]

Supporting Information

Haptotropic phenomena in digold(I) triple-bonded complexes

Ignacio Nieto-Vargas,[a] Juan Cayuela-Castillo,[a] Francisco J. Fernández-de-Córdova,[a] Israel Fernández[b]* and Pablo Ríos[a]*

[a] *Instituto de Investigaciones Químicas (IIQ), Departamento de Química Inorgánica, Centro de Innovación en Química Avanzada (ORFEO-CINQA), CSIC and Universidad de Sevilla, 41092 Sevilla (Spain).*

[b] *Departamento de Química Orgánica I and Centro de Innovación en Química Avanzada (ORFEO-CINQA), Facultad de Químicas, Universidad Complutense de Madrid, Madrid 28040, Spain*

Email: israel@quim.ucm.es; prios1@us.es

1. General Considerations **2**

2. Synthesis and characterization **3**

3. NMR spectra **8**

4. Variable-temperature NMR (VT-NMR) spectra **14**

4.1. Determination of the thermochemical parameters for the π,π exchange **22**

5. Vibrational spectra **24**

6. X-ray crystallography **26**

7. Computational studies **34**

8. Calculated energy values of the exchange processes **36**

9. Cartesian coordinates of the optimized structures **38**

10. References **104**

1. General considerations

Unless stated otherwise, all reactions were performed in a glovebox or on a Schlenk line under an atmosphere of pure Ar or high purity N_2 using standard Schlenk techniques. All solvents were dried and degassed prior to use. C_6D_6 was distilled under Ar and stored over 3 Å molecular sieves for at least 24 h prior to use. $CDCl_3$,CD_2Cl_2 and 1,2-dichlorobenzene were dried over calcium hydride before being distilled and degassed by three freeze-pump-thaw cycles. IPrAuOTf,[1] IPrCuOTf,[2] $CDCl_2F$[3] and complexes **1**[4] and **4**[5] were prepared according to literature procedures. All other reagents were purchased from commercial suppliers and used as received. $CDCl_2F$ was distilled and stored over 3 Å molecular sieves in a sealed vessel at -24 °C.

NMR spectroscopy

NMR spectra were recorded on Bruker Avance NEO 500, Avance NEO 400, Avance NEO 300 spectrometers, and they were referenced to external $SiMe_4$ (δ 0 ppm) using the residual protio solvent peaks as internal standard (^{1}H NMR experiments) or the characteristic resonances of the solvent nuclei (^{13}C NMR experiments). In the case of $CDCl_2F$, the residual solvent peak of $CHCl_2F$ consists of a doublet at δ = 7.47 ppm. Spectral assignments were made by routine one- and two-dimensional NMR experiments where appropriate.

Elemental analysis

Elemental analyses were performed by José Manuel Pérez Falcón at the Microanalytical Facility at IIQ (Instituto de Investigaciones Químicas de Sevilla), using a LECO TruSpec CHN analyzer for determination of %C, %H and %N.

Vibrational spectroscopy

FT-IR spectra were acquired using a Bruker Tensor 27 spectrometer with a Platinum ATR accessory (Thermo Scientific Nicolet iS5 iD7).

2. Synthesis and characterization

- **[IPrAuC≡CAuIPr] (2)**

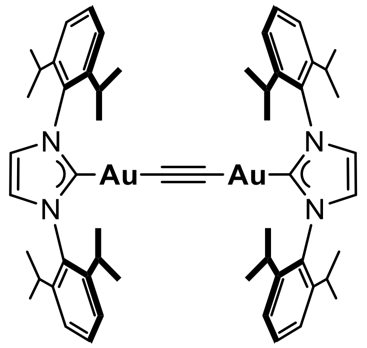

This method is based on that reported by this laboratory[4] with some modifications. In a N_2-filled glovebox, a J. Young flask was charged with a stir bar, alkynyl gold complex **IPrAuCCSiMe$_3$** [4] (235 mg, 0.344 mmol) and gold trimethylsiloxide **4** (232 mg, 0.344 mmol). Dry toluene (4 mL) was added to the mixture of solids, and the resulting colorless solution was left stirring at 120 °C in an oil bath for 48 h. Then, the resulting pale yellow solution was concentrated under vacuum to approximately 1 mL. *From this point, subsequent work-up was carried out under air*. Addition of *n*-pentane (10 mL) under vigorous stirring resulted in the immediate precipitation of bimetallic acetylide **2**. The supernatant was carefully removed, and the precipitate was dissolved in THF (3 mL) and transferred to a vial. The colorless solution was layered with *n*-pentane (15 mL) and the resulting bilayer was left to stand at 4 ºC for 1 day, after which colorless crystals were observed. The supernatant was carefully removed, and the crystals were washed with *n*-pentane (5 mL). Then, the product was dried under vacuum, giving complex **2** as a colorless solid (308 mg, 0.258 mmol, 75% yield). The washing solutions were used for a second batch of crystals. To this end, the solvent was evaporated under vacuum and the bilayer crystallization system was repeated, giving a second crop of 52 mg (0.0435 mmol). **Total yield:** 360 mg, 0.301 mmol, 88%.

The NMR chemical shifts for this sample in C_6D_6 match those described previously.[4]

- **[IPrAuC≡C(π-CuIPr)AuIPr][OTf] (3)**

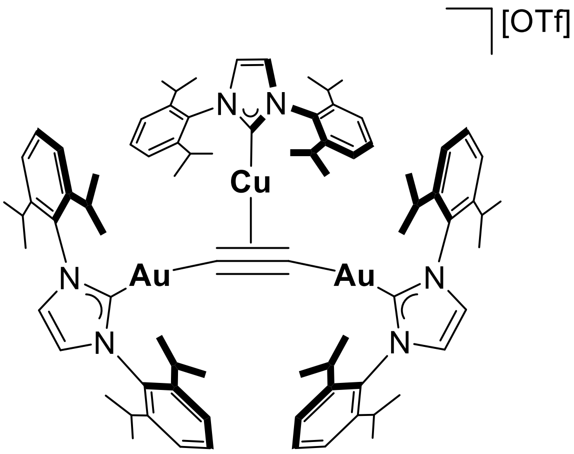

In a N_2-filled glovebox, a scintillation vial was charged with IPrAuOTf (17.9 mg, 0.0298 mmol) and a stir bar. A solution of **2** (35.6 mg, 0.0298 mmol) in CH_2Cl_2 (2 mL) was added to the vial, and the reaction mixture was left stirring at 25 °C for 3 h. Addition of *n*-pentane (10 mL) under vigorous stirring led to the immediate precipitation of a beige solid. Then, the mixture was placed in a freezer at -30 ºC overnight to ensure complete precipitation and purification of the product. The supernatant was carefully removed, and the product was dried under N_2, giving complex **3** as a beige solid (44 mg, 0.0247 mmol). Colorless crystals suitable for X-ray diffraction analysis were grown by slow diffusion of *n*-pentane into a THF solution of **3**. Crystallizing this complex affords **3** accompanied with approximately 10% of species **1** (based on ^{1}H NMR integration, see Figure SX), giving an actual 73% yield.

^{1}H NMR (400 MHz, THF-*d*$_8$, 298 K): δ 7.51 (t, $^{3}J_{H\text{-}H}$ = 7.8 Hz, 4H, σ-**Au**IPr C***H*** Ph$_{para}$), 7.48 (s, 4H, σ-**Au**IPr C***H***$_{imid}$), 7.36 (t, $^{3}J_{H\text{-}H}$ = 7.8 Hz, 2H, π-**Cu**IPr C***H*** Ph$_{para}$), 7.33 (s, 2H, π-**Cu**IPr C***H***$_{imid}$), 7.30 (d, $^{3}J_{H\text{-}H}$ = 7.8 Hz, 8H, σ-**Au**IPr C***H*** Ph$_{meta}$), 7.10 (d, $^{3}J_{H\text{-}H}$ = 7.8 Hz, 4H, π-**Cu**IPr C***H*** Ph$_{meta}$), 2.58 (sept, $^{3}J_{H\text{-}H}$ = 6.8 Hz, 8H, σ-**Au**IPr C***H***$(CH_3)_2$), 2.37 (sept, $^{3}J_{H\text{-}H}$ = 6.8 Hz, 4H, π-**Cu**IPr C***H***$(CH_3)_2$), 1.16 (t, $^{3}J_{H\text{-}H}$ = 6.3 Hz, 48H, σ-**Au**IPr CH(C***H***$_3$)$_2$), 1.04 (d, $^{3}J_{H\text{-}H}$ = 6.8 Hz, 12H, π-**Cu**IPr CH(C***H***$_3$)$_2$), 0.75 (d, $^{3}J_{H\text{-}H}$ = 6.8 Hz, 12H, π-**Cu**IPr CH(C***H***$_3$)$_2$) ppm. **^{13}C{^{1}H} NMR (101 MHz, THF-*d*$_8$, 298 K):** δ 189.4 (σ-**Au**IPr Au***C***$_{carbene}$), 179.7 (π-**Cu**IPr Au***C***$_{carbene}$), 148.7 (Au***C***$_{alkyne}$), 146.4 (σ-**Au**IPr ***C***$_q$ Ph$_{orto}$), 146.3 (π-**Cu**IPr ***C***$_q$ Ph$_{orto}$), 135.8 (σ-**Au**IPr N***C***$_q$ Ph$_{ipso}$), 135.6 (π-**Cu**IPr N***C***$_q$ Ph$_{ipso}$), 131.3 (σ-**Au**IPr ***C***H Ph$_{para}$), 130.8 (π-**Cu**IPr ***C***H Ph$_{para}$), 125.5 (σ-**Au**IPr =***C***H$_{imid}$), 125.0 (σ-**Au**IPr ***C***H Ph$_{meta}$), 124.6 (π-**Cu**IPr ***C***H Ph$_{meta}$), 124.4 (π-**Cu**IPr =***C***H$_{imid}$), 29.6 (σ-**Au**IPr ***C***H$(CH_3)_2$), 29.4 (π-**Cu**IPr ***C***H$(CH_3)_2$), 26.3 (π-**Cu**IPr CH(***C***H$_3$)$_2$), 24.9 (σ-**Au**IPr CH(***C***H$_3$)$_2$), 24.4 (σ-**Au**IPr CH(***C***H$_3$)$_2$), 23.6 (π-**Cu**IPr CH(***C***H$_3$)$_2$) ppm.

Elem. Anal. Calcd for: $C_{84}H_{108}Au_2CuF_3N_6O_3S\cdot0.1C_{84}H_{108}Au_3F_3N_6O_3S$: C, 55.79; H, 6.02; N, 4.65. Found: C, 56.19; H, 5.83; N, 4.66.

- **[IPrAuC≡C−C≡CAuIPr] (5)**

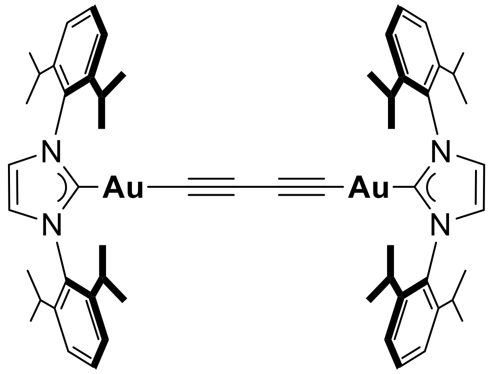

In a N_2-filled glovebox, a solution of 1,4-bis(trimethylsilyl)butadiyne (21.1 mg, 0.109 mmol) in 0.5 mL of dry toluene was added over a suspension of complex **4** (146.6 mg, 0.217 mmol) in 2 mL of the same solvent. The reaction mixture was then transferred to a J.Young flask, taken out of the glovebox and placed in an oil bath at 120°C for 4 days. At this point, the resulting brown solution was transferred to a 13 mL scintillation vial. Addition of *n*-pentane (6 mL) under vigorous stirring resulted in the immediate precipitation of a brown solid. After 40 minutes, the light-yellow supernatant was carefully removed and the precipitate was vacuum dried in the Schlenk line for 2 hours, resulting in complex **5** as a pale brown solid (114.9 mg, 0.0942 mmol, 87% yield).

^{1}H NMR (500 MHz, C_6D_6, 298 K): δ 7.20 (t, $^3J_{H\text{-}H}$ = 7.8 Hz, 4H, C**<u>H</u>** Ph_{para}), 7.02 (d, $^3J_{H\text{-}H}$ = 7.8 Hz, 8H, C**<u>H</u>** Ph_{meta}), 6.24 (s, 4H, C**<u>H</u>**$_{imid}$), 2.50 (sept, $^3J_{H\text{-}H}$ = 6.9 Hz, 8H, C**<u>H</u>**$(CH_3)_2$), 1.29 (d, $^3J_{H\text{-}H}$ = 6.9 Hzz, 24H, CH(C**<u>H</u>**$_3)_2$), 1.03 (d, $^3J_{H\text{-}H}$ = 6.9 Hz, 24H, CH(C**<u>H</u>**$_3)_2$) ppm. **^{13}C{^{1}H} NMR (126 MHz, C_6D_6, 298 K):** δ 192.7 (Au**<u>C</u>**$_{carbene}$), 145.7 (**<u>C</u>**$_q$ Ph_{orto}), 134.7 (N**<u>C</u>**$_q$ Ph_{ipso}), 130.6 (**<u>C</u>**H Ph_{para}), 124.2 (**<u>C</u>**H Ph_{meta}), 122.6 (=**<u>C</u>**H_{imid}), 114.7 (**<u>C</u>**$_{alkyne}$), 91.1 (**<u>C</u>**$_{alkyne}$), 29.00 (**<u>C</u>**H $(CH_3)_2$), 24.80 (CH (**<u>C</u>**$H_3)_2$), 24.00 (CH (**<u>C</u>**$H_3)_2$) ppm.

Elem. Anal. Calcd for: $C_{58}H_{72}Au_2N_4$: C 57.14%; H 5.95%; N 4.60; Found: C 57.22%; H 5.77%; N 4.44.

- **[IPrAuC≡C(π-AuIPr)C≡CAuIPr][OTf] (6)**

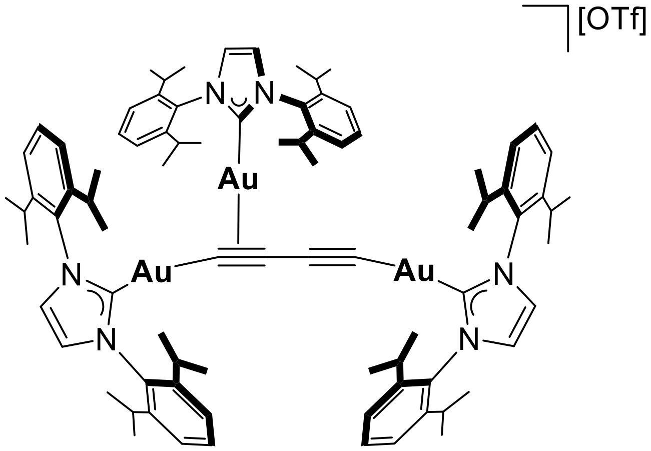

In a N_2-filled glovebox, a solution of IPrAuOTf (19.4 mg, 0.0264 mmol) in 0.3 mL of dry THF was added over a solution of complex **5** (33.2 mg, 0.0264 mmol) in 0.4 mL of the same solvent, stirring the reaction mixture for 2 hours. Then, the magnetic stir bar was removed, and 5 mL of *n*-pentane were carefully added over the resulting yellow solution, creating a bilayer that was left to stand overnight. The supernatant was discarded, and the resulting yellow crystals were vigorously stirred in 4 mL of *n*-pentane for 40 minutes. At this point, the colorless supernatant was carefully removed, and the solid was left to dry overnight in the N_2-filled atmosphere, giving complex **6** as a yellow powder (48.0 mg, 0.0246 mmol, 93% yield).

^{1}H NMR (400 MHz, $CDCl_3$, 243 K): δ 7.47 (t, $^3J_{H-H}$ = 7.8 Hz, 2H, π-AuIPr: C**H** Ph_{para}), 7.42 (t, $^3J_{H-H}$ = 7.8 Hz, 4H, σ-AuIPr: C**H** Ph_{para}), 7.25 (d, $^3J_{H-H}$ = 7.7 Hz, 4H, π-AuIPr: C**H** Ph_{meta}), 7.20 (s, 2H, π-AuIPr: C**H**$_{imid}$), 7.17 (s, 4H, σ-AuIPr: C**H**$_{imid}$), 7.15 (d, $^3J_{H-H}$ = 7.8 Hz, 8H, σ-AuIPr: C**H** Ph_{meta}), 2.41 (sept, $^3J_{H-H}$ = 6.7 Hz, 4H, π-AuIPr: C**H**$(CH_3)_2$), 2.27 (sept, $^3J_{H-H}$ = 6.7 Hz, 8H, σ-AuIPr: C**H**$(CH_3)_2$), 1.23 (d, 12H, $^3J_{H-H}$ = 6.6 Hz, π-AuIPr: CH(C**H**$_3$)$_2$), 1.19 (d, $^3J_{H-H}$ = 6.6 Hz, 12H, π-AuIPr: CH(C**H**$_3$)$_2$), 1.11 (d, $^3J_{H-H}$ = 6.8 Hz, 24H, σ-AuIPr: CH(C**H**$_3$)$_2$), 0.90 (d, $^3J_{H-H}$ = 6.8 Hz, 24H, σ-AuIPr: CH(C**H**$_3$)$_2$) ppm. **^{13}C{^{1}H} NMR (101 MHz, $CDCl_3$, 243 K):** δ 187.8 (π-AuIPr: **C**$_{carbene}$), 181.6 (σ-AuIPr: **C**$_{carbene}$), 145.4 (π-AuIPr: **C**$_q$ Ph_{orto}), 145.1 (σ-AuIPr: **C**$_q$ Ph_{orto}), 133.7 (π-AuIPr: N**C**$_q$ Ph_{ipso}), 133.4 (σ-AuIPr: N**C**$_q$ Ph_{ipso}), 130.6 (σ-AuIPr: **C**H Ph_{para}), 130.5 (π-AuIPr: **C**H Ph_{para}), 124.1 (σ-AuIPr: **C**H Ph_{meta}), 124.0 (σ-AuIPr: =**C**H$_{imid}$), 123.7 (π-AuIPr: **C**H Ph_{meta}), 123.5 (π-AuIPr: =**C**H$_{imid}$), 103.3 (**C**$_{alkyne}$), 99.1 (**C**$_{alkyne}$), 28.7 (π-AuIPr: **C**H$(CH_3)_2$), 28.5 (σ-AuIPr: **C**H$(CH_3)_2$), 25.0 (π-AuIPr: CH(**C**H$_3$)$_2$), 25.0 (σ-AuIPr: CH(**C**H$_3$)$_2$), 23.9 (π-AuIPr: CH(**C**H$_3$)$_2$), 23.8 (σ-AuIPr: CH(**C**H$_3$)$_2$) ppm.

Elem. Anal. Calcd for: $C_{86}H_{108}Au_3N_6F_3SO_3$: C 52.87; H 5.57; N 4.30; Found: C 52.95; H 5.68; N 4.10.

- **[IPrAuC≡C(π-CuIPr)C≡CAuIPr][OTf] (7)**

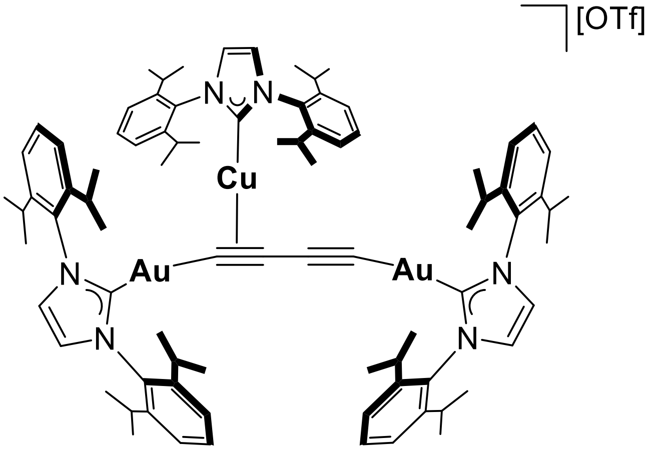

In a N_2-filled glovebox, a solution of IPrCuOTf (17.3 mg, 0.0288 mmol) in 0.6 mL of dry THF was added over a solution of complex **5** (35.0 mg, 0.0287 mmol) in 0.4 mL of the same solvent, stirring the reaction mixture for 1.5 hours. Then, the magnetic stir bar was removed, and 5 mL of *n*-pentane were carefully added over the resulting solution, creating a bilayer that was left to stand overnight. The following day, the supernatant was discarded, and the resulting colorless crystals were vigorously stirred with 4 mL of *n*-pentane for 1 hour. At this point, the supernatant was carefully removed, and the solid was left to dry overnight in the N_2-filled atmosphere, giving complex **7** as a colorless powder (48.0 mg, 0.0263 mmol, 92% yield).

^{1}H NMR (400 MHz, CD_2Cl_2, 298 K): δ 7.53 (t, $^3J_{H\text{-}H}$ = 7.8 Hz, 4H, σ-AuIPr: C**H** Ph$_{para}$), 7.39 (t, $^3J_{H\text{-}H}$ = 7.8 Hz, 2H, π-CuIPr: C**H** Ph$_{para}$), 7.30 (d, $^3J_{H\text{-}H}$ = 7.8 Hz, 8H, σ-AuIPr: C**H** Ph$_{meta}$), 7.18 (s, 4H, σ-AuIPr: C**H**$_{imid}$), 7.13 (d, $^3J_{H\text{-}H}$ = 7.8 Hz, 4H, π-CuIPr: C**H** Ph$_{meta}$), 7.11 (s, 2H, π-CuIPr: C**H**$_{imid}$), 2.47 (sept, $^3J_{H\text{-}H}$ = 6.9 Hz, 8H, σ-AuIPr: C**H**(CH_3)$_2$), 2.32 (sept, $^3J_{H\text{-}H}$ = 6.7 Hz, 4H, π-CuIPr: C**H**(CH_3)$_2$), 1.19 (m, 48H, σ-AuIPr: CH(C**H**$_3$)$_2$), 1.08 (d, $^3J_{H\text{-}H}$ = 6.8 Hz, 12H, π-CuIPr: CH(C**H**$_3$)$_2$), 0.78 (d, $^3J_{H\text{-}H}$ = 6.8 Hz, 12H, π-CuIPr: CH(C**H**$_3$)$_2$) ppm. **^{13}C{^{1}H} NMR (101 MHz, CD_2Cl_2 , 298 K):** δ 187.7 (Au: **C**$_{carbene}$), 178.1 (Cu: **C**$_{carbene}$), 146.0 (Au: **C**$_q$ Ph$_{orto}$), 145.8 (Cu: **C**$_q$ Ph$_{orto}$), 134.4 (Cu: N**C**$_q$ Ph$_{ipso}$), 134.4 (Au: N**C**$_q$ Ph$_{ipso}$), 131.1 (Au: **C**H Ph$_{para}$), 130.8 (Cu: **C**H Ph$_{para}$), 124.6 (Au: **C**H Ph$_{meta}$), 124.4 (Cu: **C**H Ph$_{meta}$), 124.21 (Au: =**C**H$_{imid}$), 124.03 (Cu: =**C**H$_{imid}$), 117.0 (**C**$_{alkyne}$), 91.5 (**C**$_{alkyne}$), 29.1 (Au: **C**H(CH_3)$_2$), 29.0 (Cu: **C**H(CH_3)$_2$), 25.5 (Cu: CH(**C**H$_3$)$_2$), 24.7 (Au: CH(**C**H$_3$)$_2$), 24.1 (Au: CH(**C**H$_3$)$_2$), 23.9 (Cu: CH(**C**H$_3$)$_2$) ppm.

Elem. Anal. Calcd for: $C_{86}H_{108}Au_2CuN_6F_3SO_3$: C 56.74; H 5.98; N 4.62. Found: 56.93; H 6.09; N 4.41.

3. NMR spectra

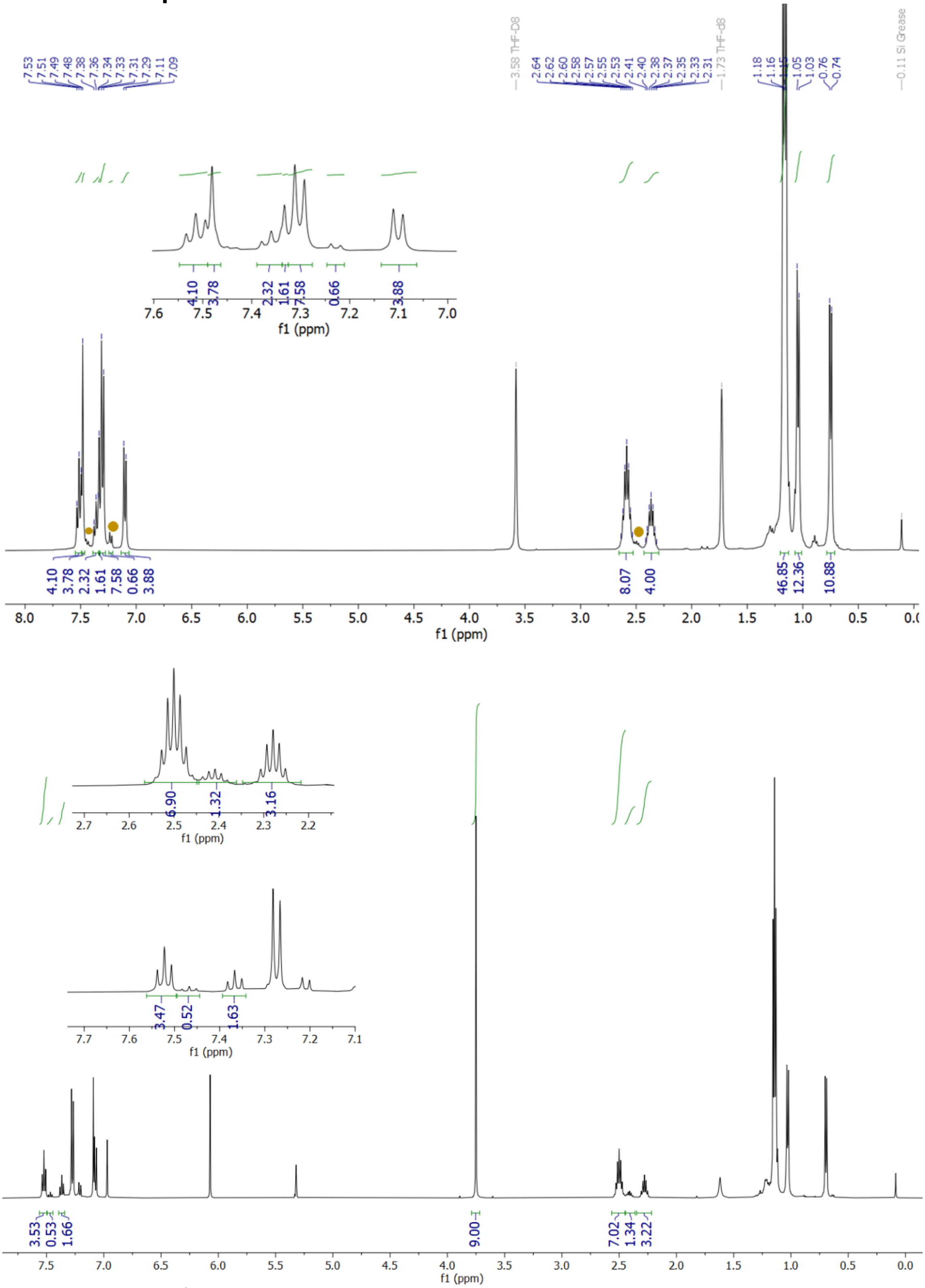

Figure S1. Top: ^{1}H NMR spectrum (THF-d_8, 400 MHz, 298 K) of complex **3**. The ochre circles indicate small amounts of complex **1** present. Integration of the aromatic resonances (7.22 and 7.10 ppm) give a relative 10% ratio of complex **1** in the sample. Inset: expanded view of the aromatic region. Bottom: ^{1}H NMR spectrum (CD_2Cl_2, 400 MHz, 298 K) of complex **3** in the presence of 1,3,5-trimethoxybenzene (1 equiv.), after standing at 25 °C for 21 h following mixing the reagents at -78 °C.

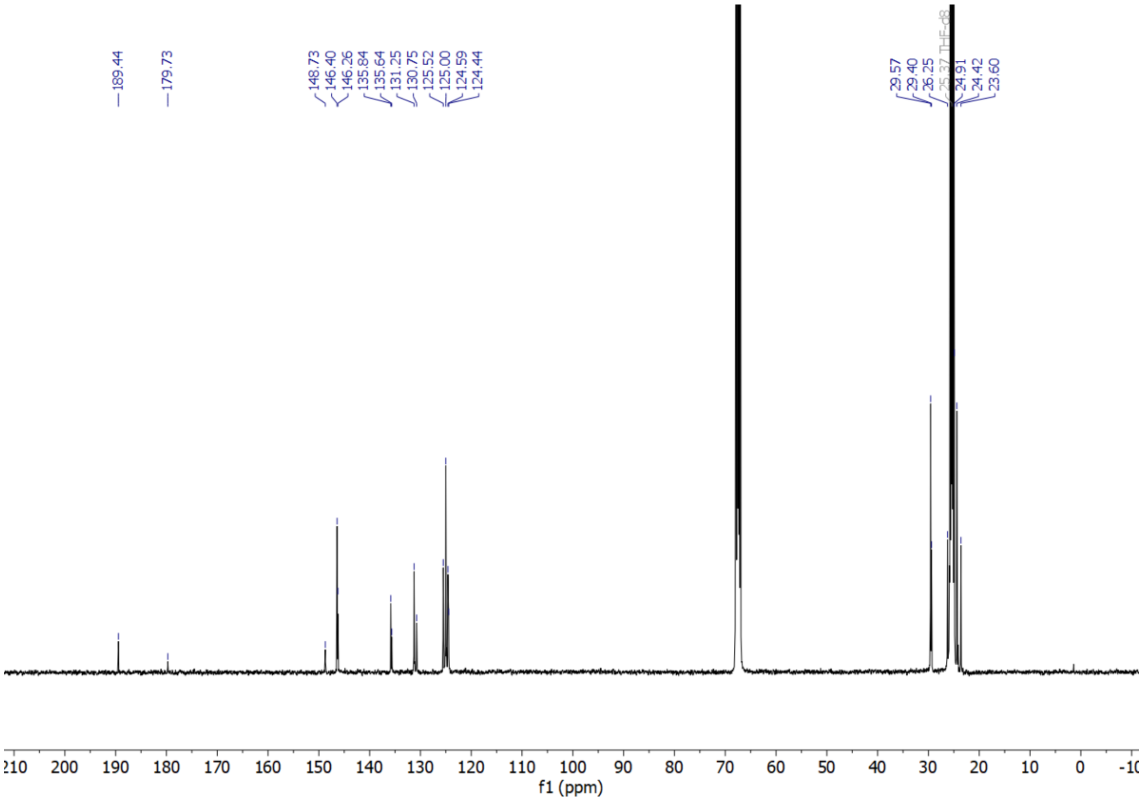

Figure S2. $^{13}C\{^{1}H\}$ NMR spectrum (THF-d_8, 101 MHz, 298 K) of complex **3**.

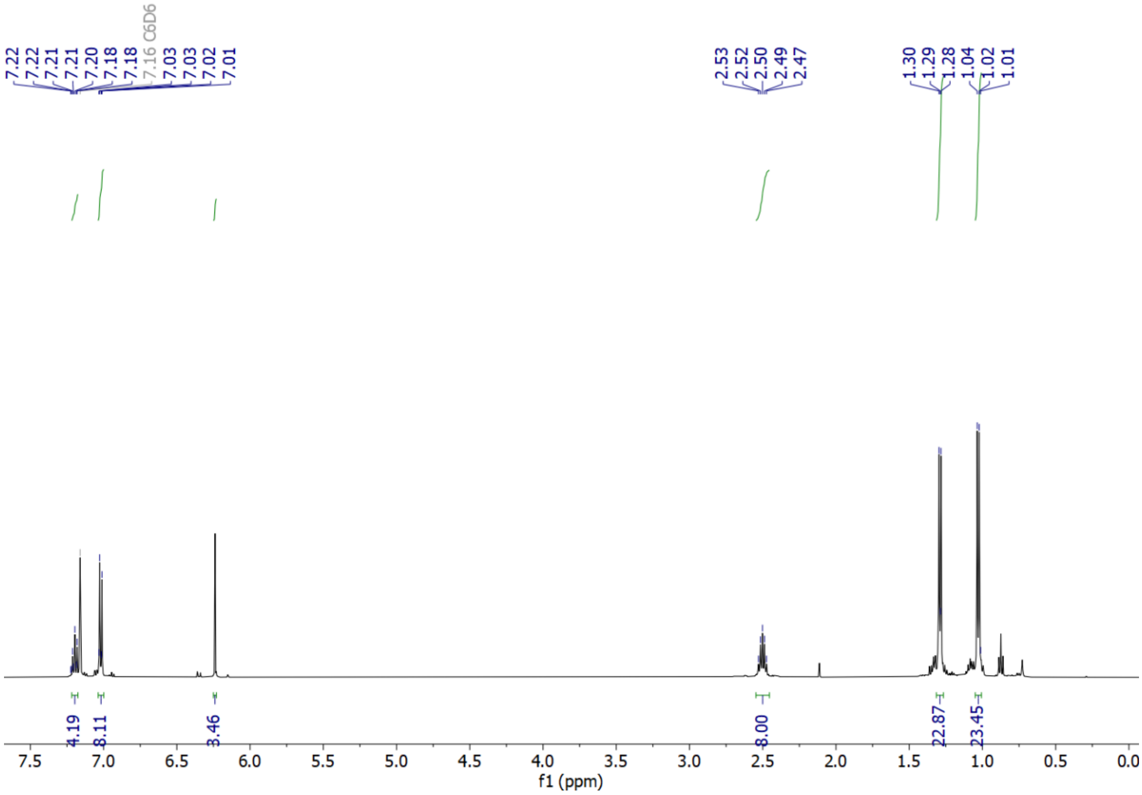

Figure S3. ^{1}H NMR spectrum (C_6D_6, 500 MHz, 298 K) of complex **5**.

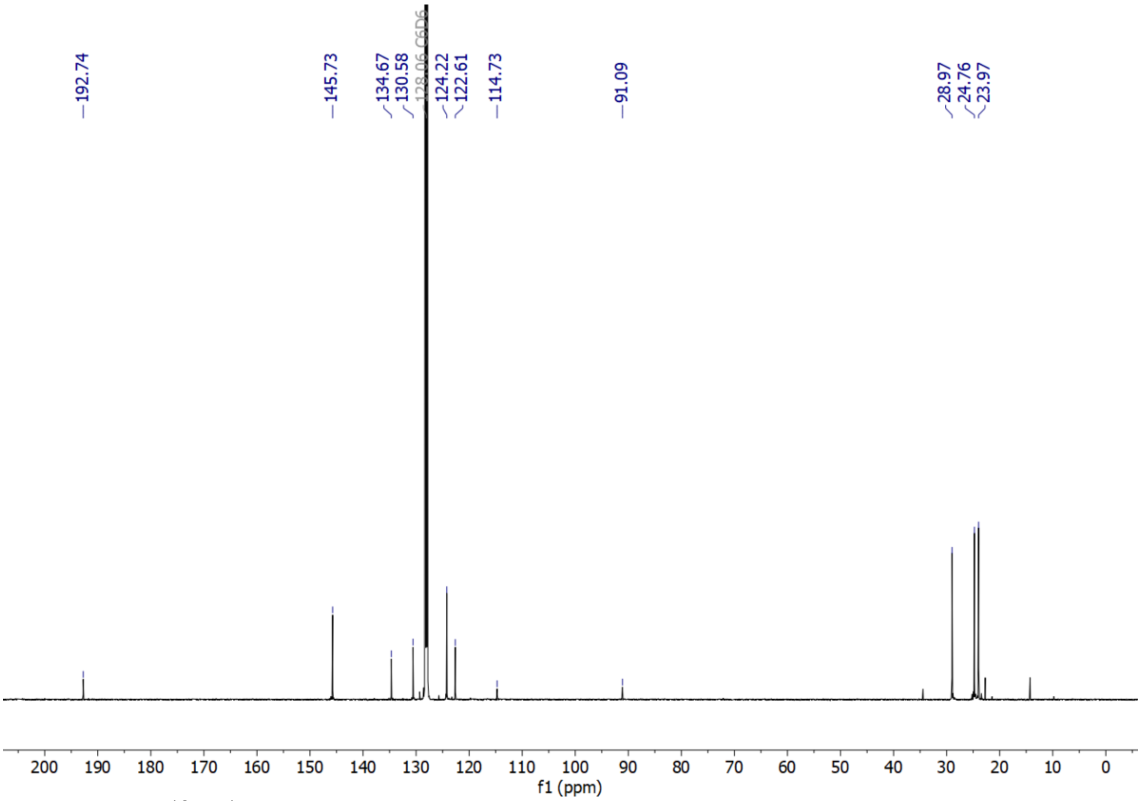

Figure S4. $^{13}C\{^{1}H\}$ NMR spectrum (C_6D_6, 126 MHz, 298 K) of complex **5**.

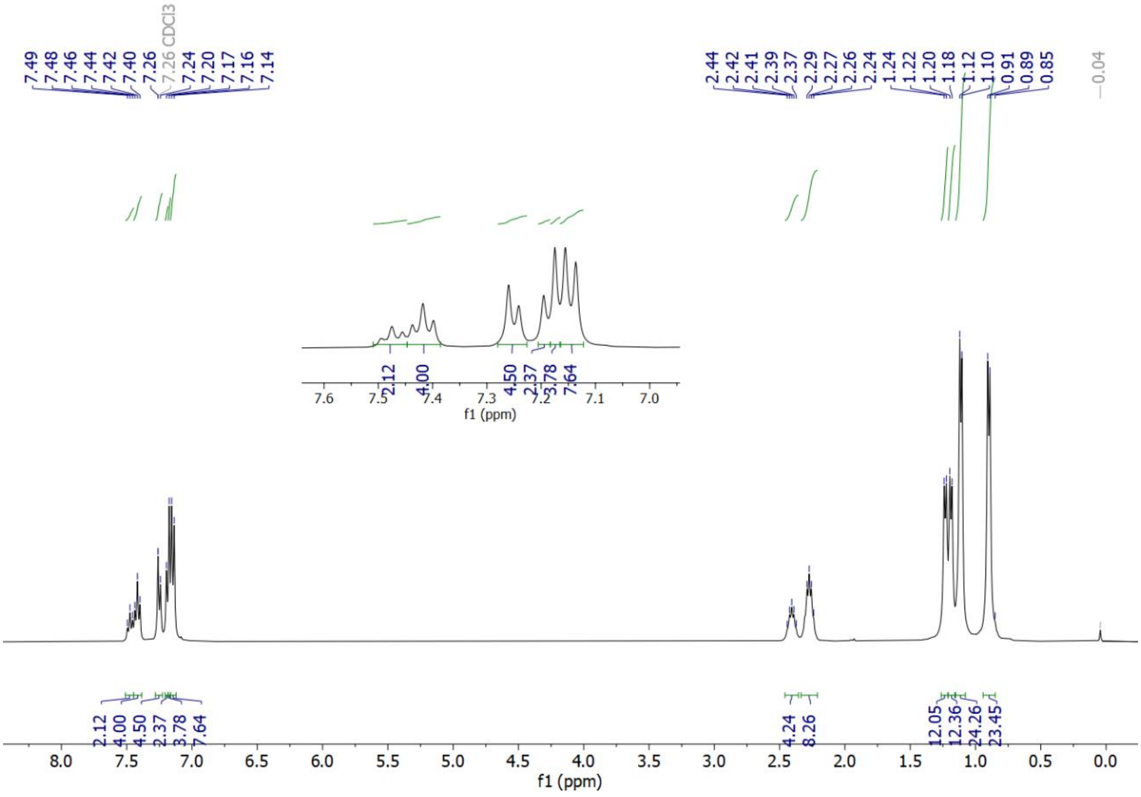

Figure S5. ^{1}H NMR spectrum ($CDCl_3$, 400 MHz, 243 K) of complex **6**. Inset: expanded view of the aromatic region.

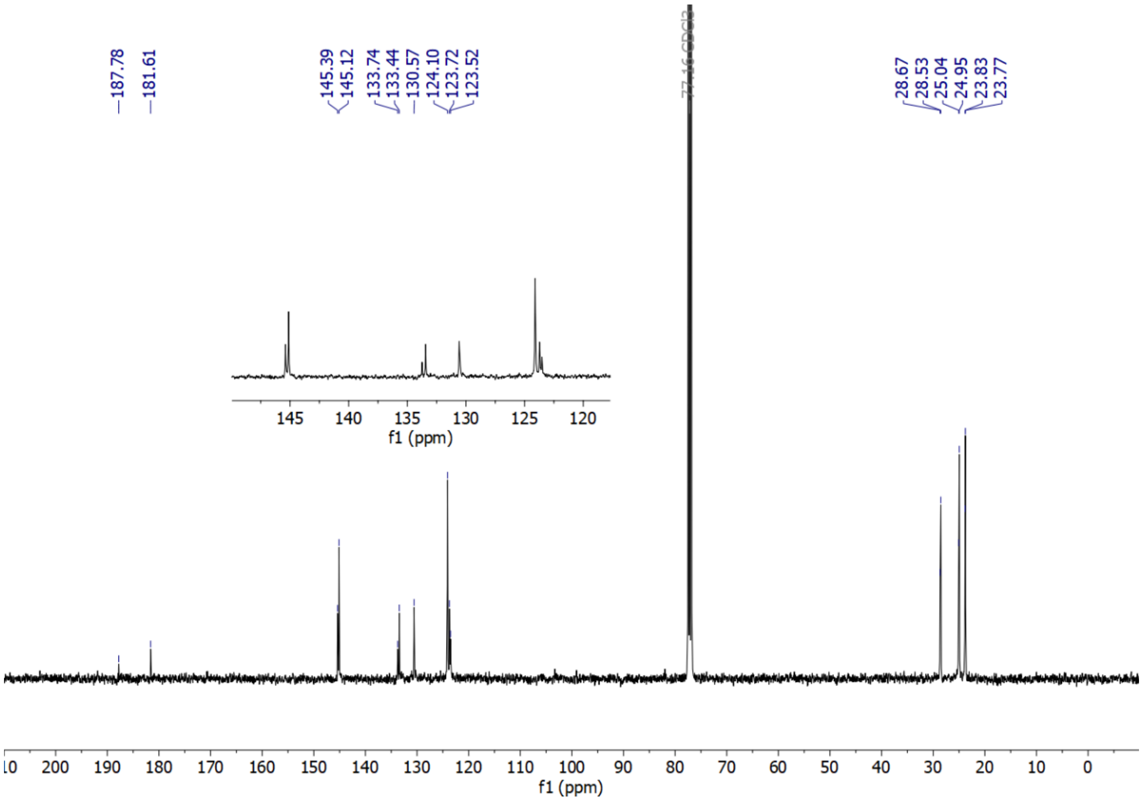

Figure S6. $^{13}C\{^1H\}$ NMR spectrum ($CDCl_3$, 400 MHz, 243 K) of complex **6**. Inset: expanded view of the aromatic region.

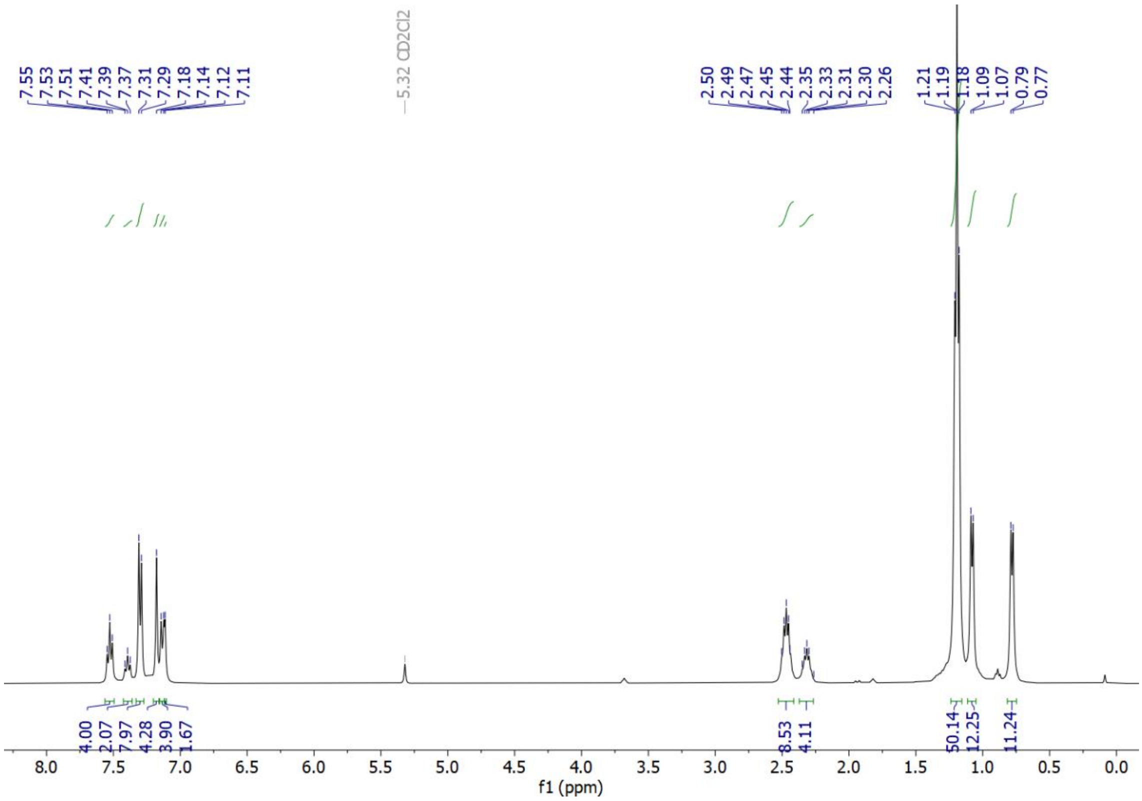

Figure S7. ^1H NMR spectrum (CD_2Cl_2, 400 MHz, 298 K) of complex **7**.

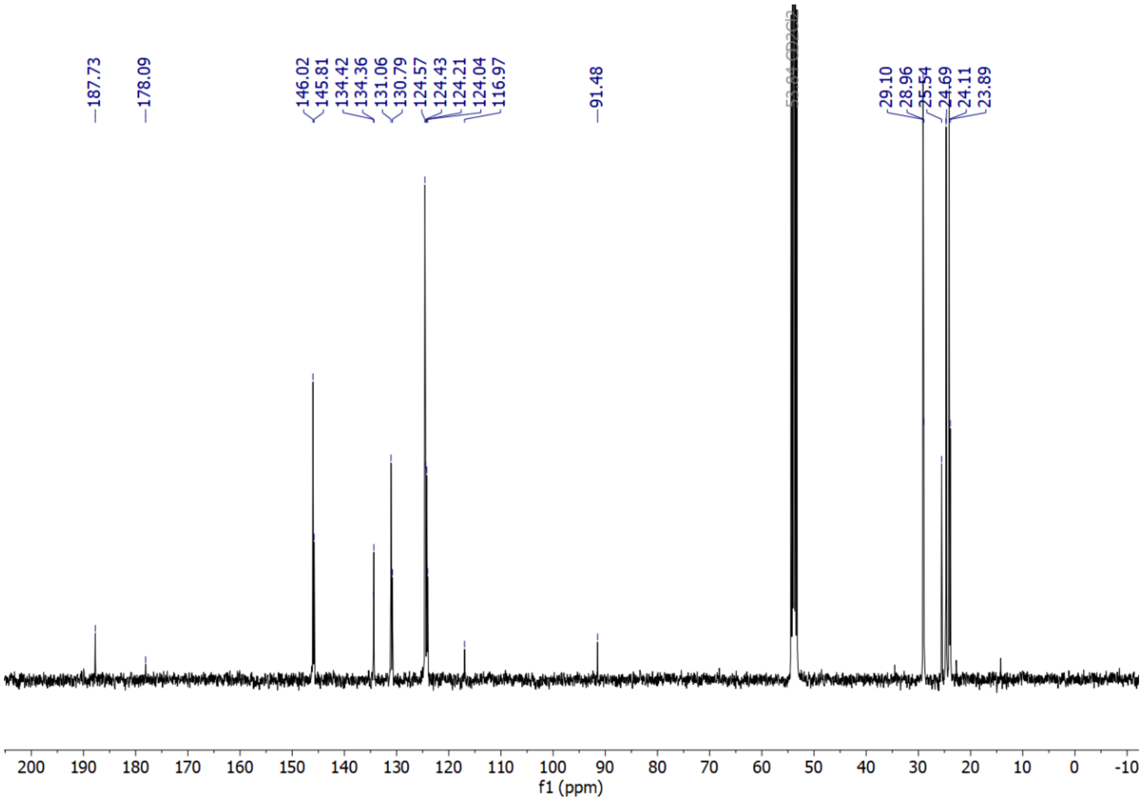

Figure S8. $^{13}C\{^{1}H\}$ NMR spectrum (CD_2Cl_2, 400 MHz, 298 K) of complex **7**.

*Anion exchange in complexes **1** and **6***

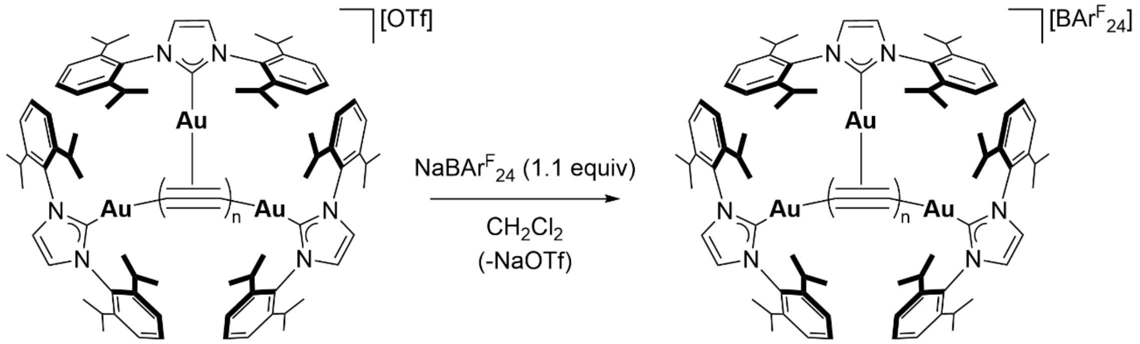

1: n = 1
6: n = 2

1·BAr$^F_{24}$: n = 1
6·BAr$^F_{24}$: n = 2

Under air, a dram vial was charged with either **1** or **6** (20 mg) and NaBAr$^F_{24}$ (1.1 equiv, approximately 10 mg). Dry dichloromethane (1 mL) was added to the mixture of solids, and the reaction mixture was left stirring at 25 °C for 1.5 h. Then, the reaction mixture was filtered through celite (1 cm height in a Pasteur pipette) to a scintillation vial. The reaction vial and filter were rinsed with dry dichloromethane (1 mL), after which an aliquot was taken to be analyzed by ^{1}H and ^{19}F NMR to ensure full conversion. The aliquot was poured back into the reaction mixture, which was evaporated under vacuum giving a residue that was washed with *n*-pentane (5 mL). The resulting solid was dried under vacuum until constant mass. The expected products were obtained in quantitative yields (27 mg for **1·BAr$^F_{24}$** and 26 mg for **6·BAr$^F_{24}$**).

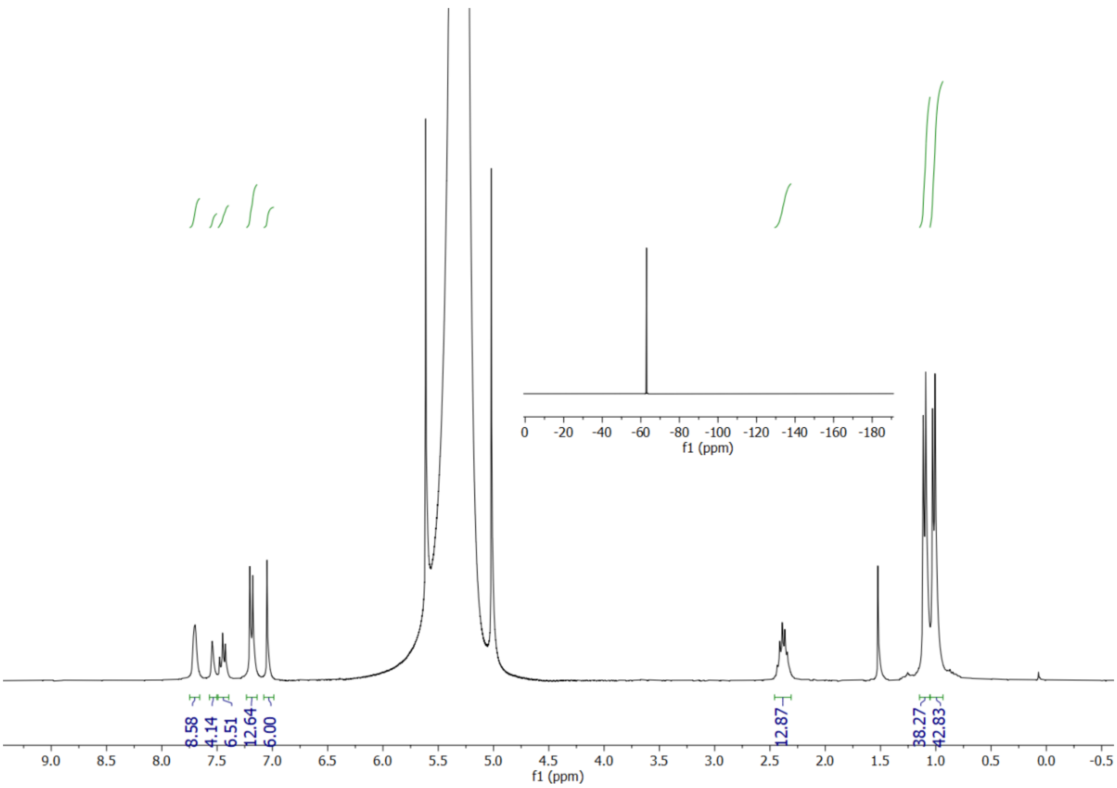

Figure S9. ^{1}H NMR spectrum (CH_2Cl_2, 300 MHz, 298 K) of complex **1·BAr$^F_{24}$**. Inset: ^{19}F{^{1}H} NMR spectrum (CH_2Cl_2, 282 MHz, 298 K). No resonance attributable to the trifluoromethanesulfonate anion is observed.

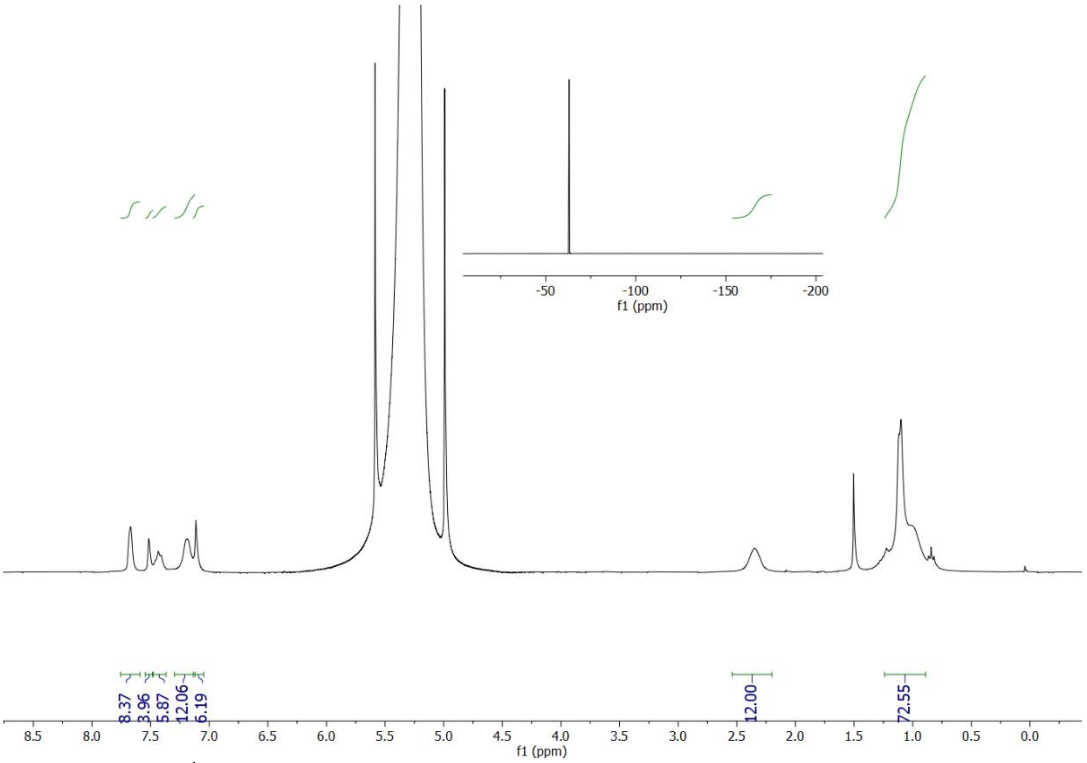

Figure S10. ^{1}H NMR spectrum (CH_2Cl_2, 300 MHz, 298 K) of complex **6·BAr$^F_{24}$**. Inset: ^{19}F{^{1}H} NMR spectrum (CH_2Cl_2, 282 MHz, 298 K). No resonance attributable to the trifluoromethanesulfonate anion is observed.

4. Variable-temperature NMR (VT-NMR) spectra

In the case of VT-NMR experiments involving $CDCl_2F$, approximately 20 mg of trinuclear acetylide or diacetylide complex were placed inside a screw-cap NMR tube, after which the solvent was added under Ar. Immediately after, the NMR tube was capped, placed in a cold bath (-60 °C) and manually shaken until the sample completely dissolved. Then, the NMR tube was placed inside a pre-cooled (0 °C) NMR spectrometer.

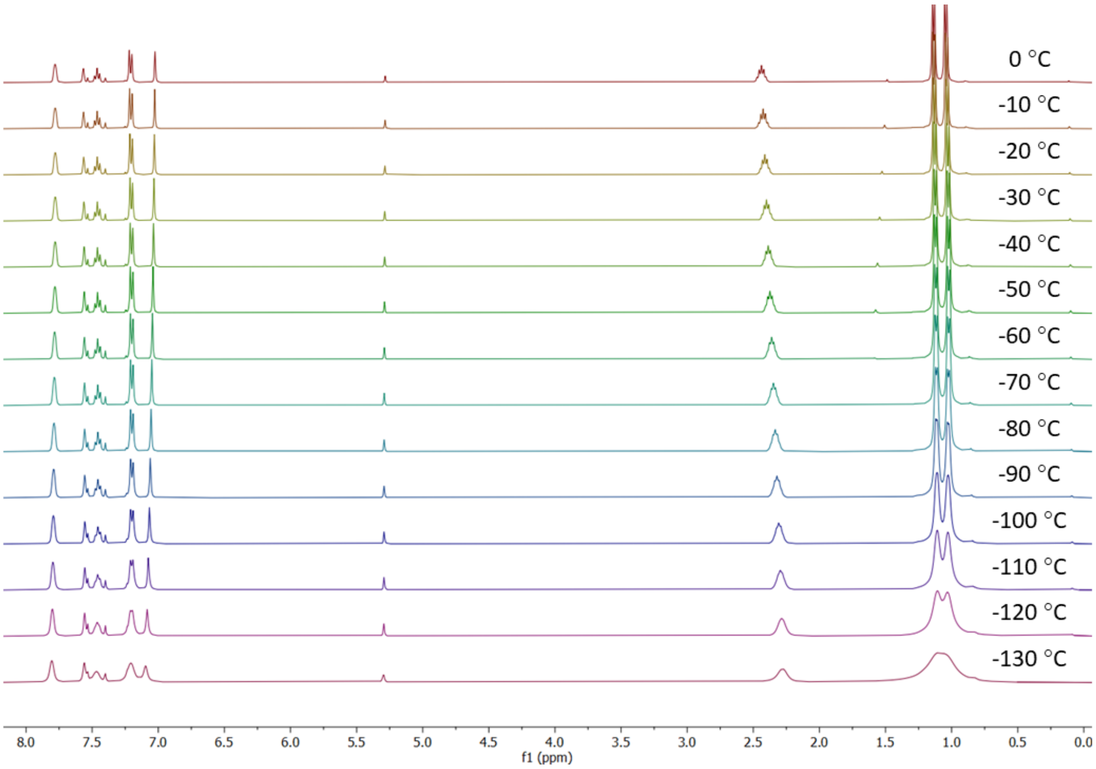

Figure S11. Variable-temperature ^1H NMR spectra (400 MHz, $CDCl_2F$) of complex **1·BAr^F$_{24}$**. The peak at approximately 5.3 ppm corresponds to residual dichloromethane.

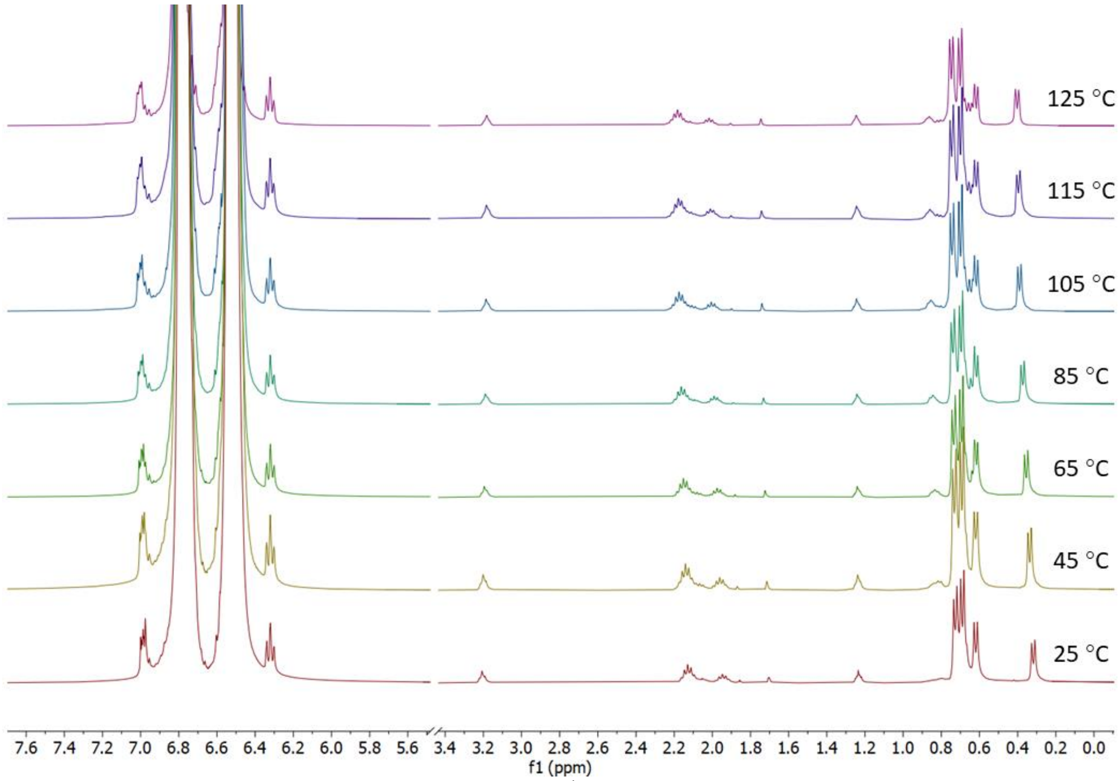

Figure S12. Variable-temperature ^{1}H NMR spectra (400 MHz, 1,2-dichlorobenzene) of complex **3**. The peaks at approximately 1.2 and 3.2 ppm correspond to residual tetrahydrofuran.

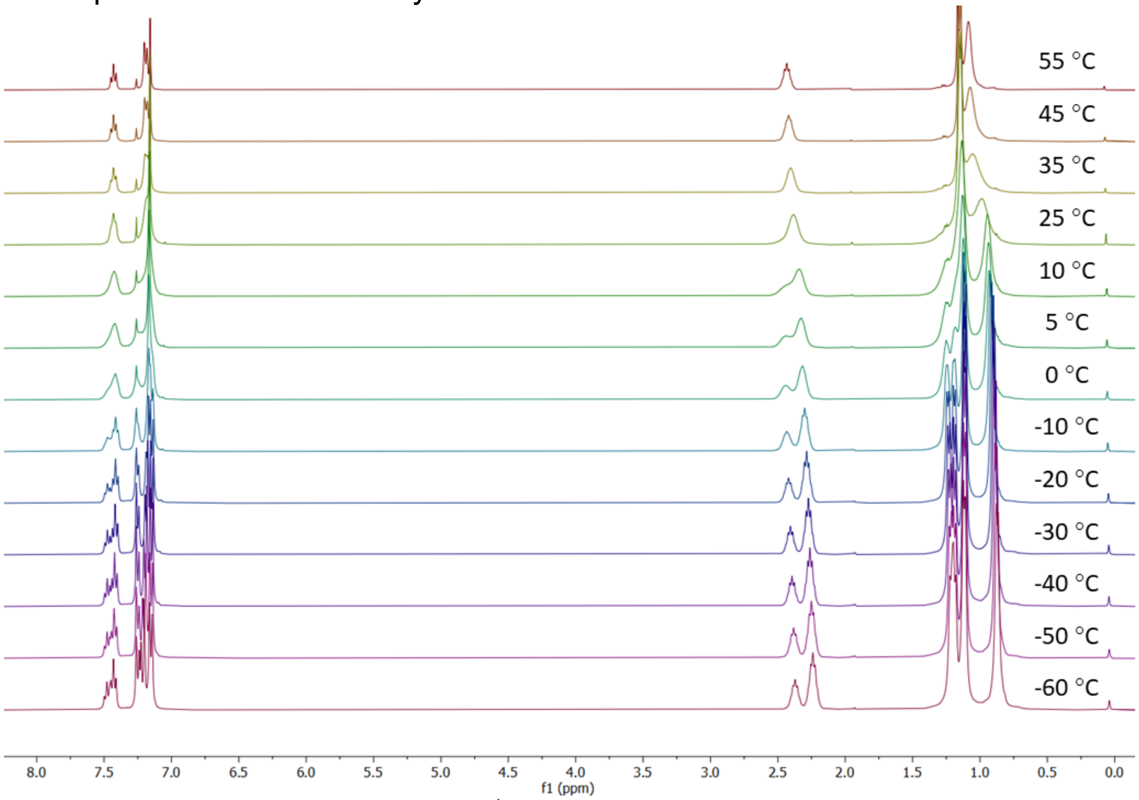

Figure S13. Variable-temperature ^{1}H NMR spectra (400 MHz, $CDCl_3$) of complex **6**.

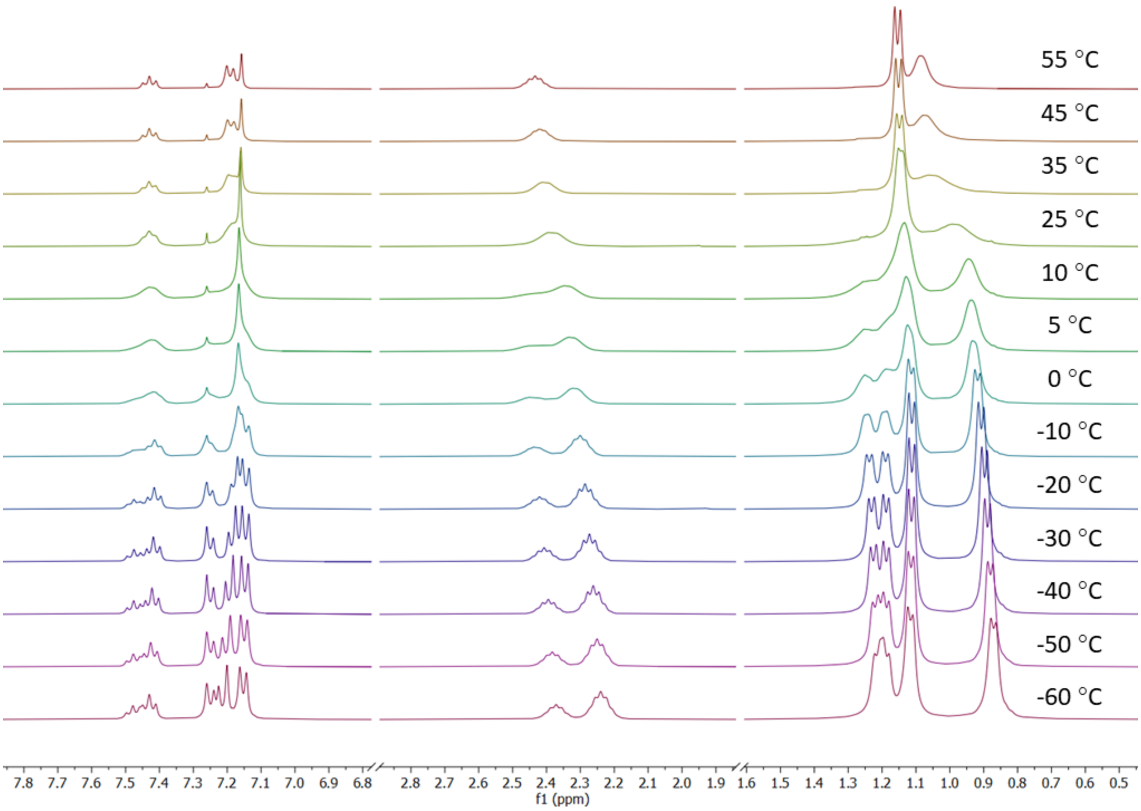

Figure S14. Expanded view of the variable-temperature ^{1}H NMR spectra (400 MHz, $CDCl_3$) of complex **6**. At 55 °C, one of the resonances attributable to the methyl groups has not yet fully coalesced into a sharp, well-resolved signal.

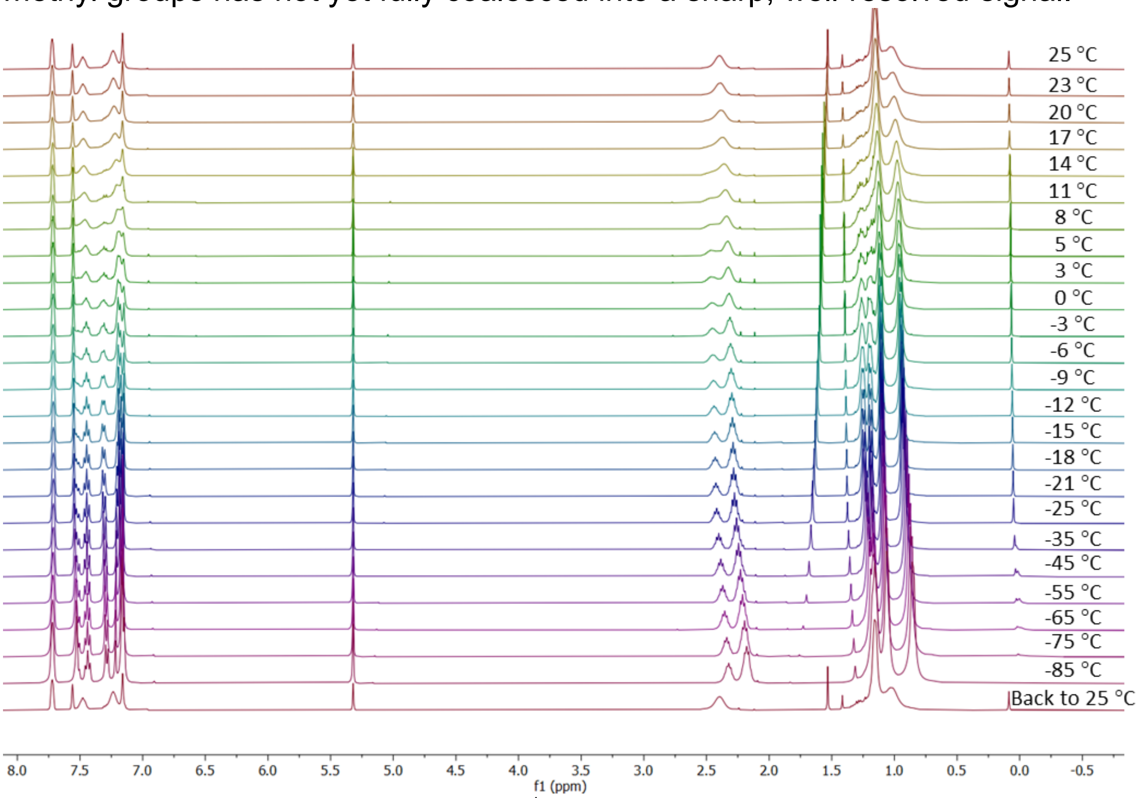

Figure S15. Variable-temperature ^{1}H NMR spectra (400 MHz, CD_2Cl_2) of complex **6·BAr^F$_{24}$**.

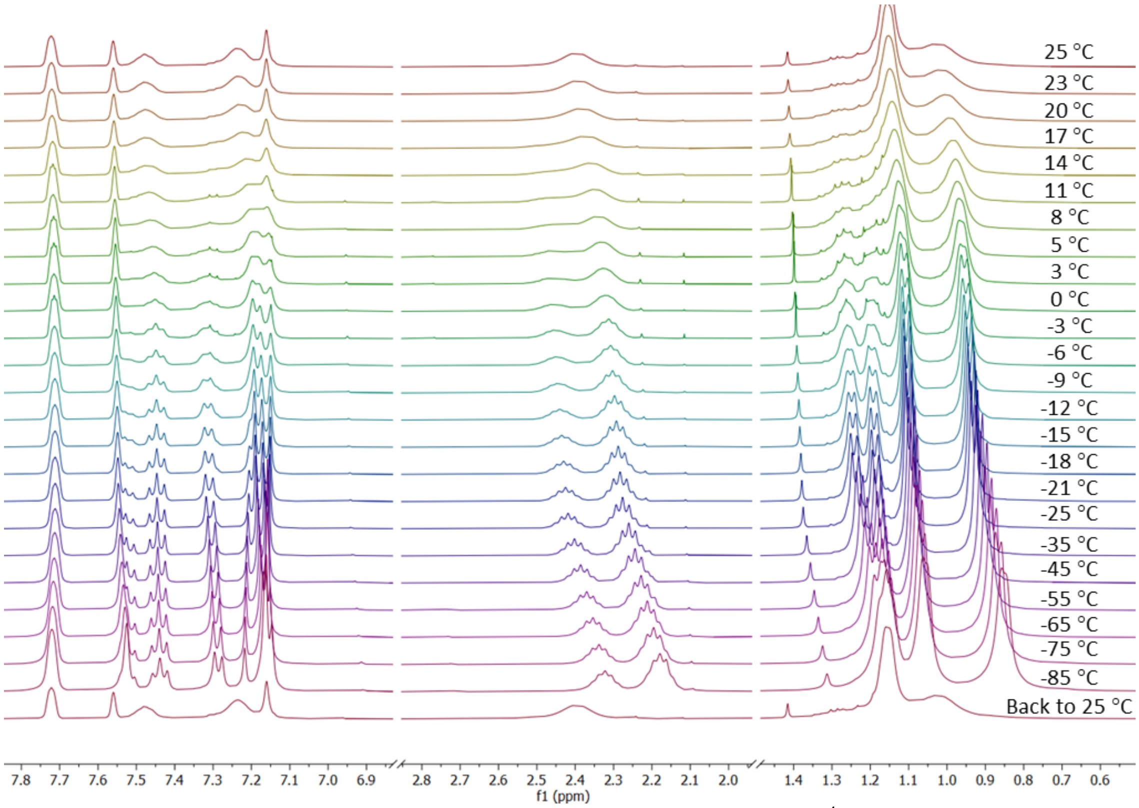

Figure S16. Expanded view of the variable-temperature ^{1}H NMR spectra (400 MHz, CD_2Cl_2) of complex **6·BAr^F$_{24}$**.

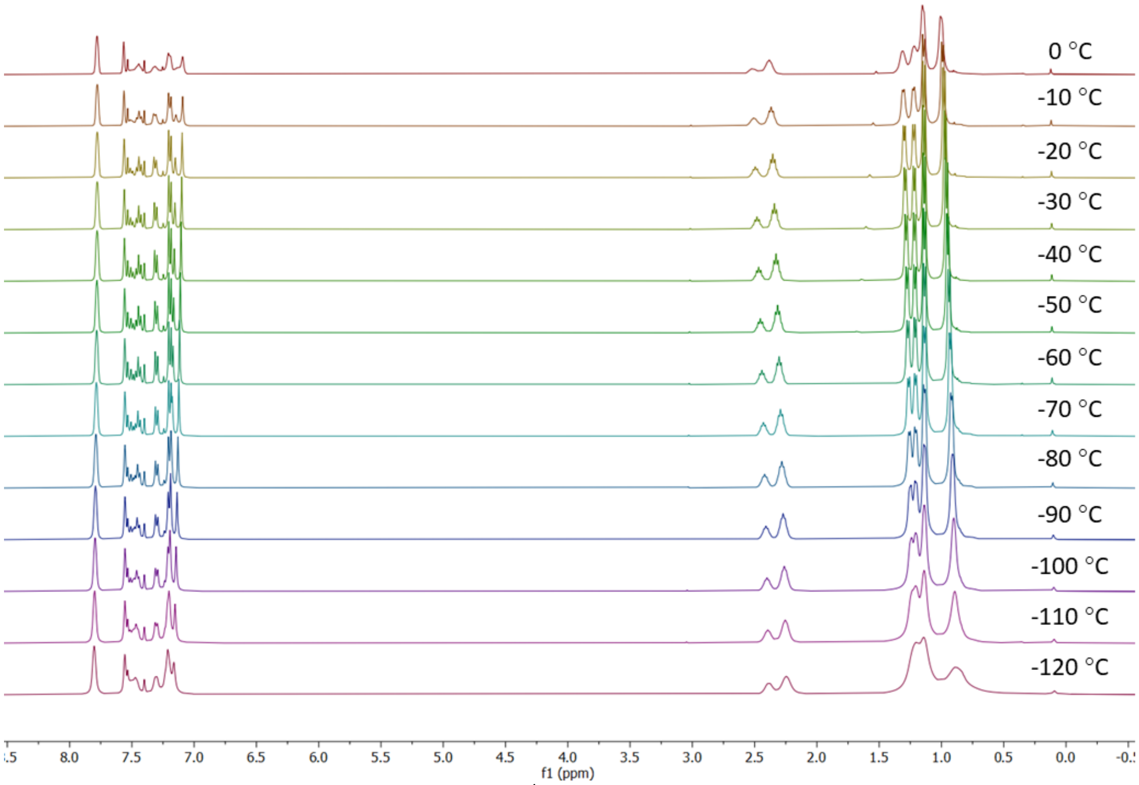

Figure S17. Variable-temperature ^{1}H NMR spectra (400 MHz, $CDCl_2F$) of complex **6·BAr^F$_{24}$**.

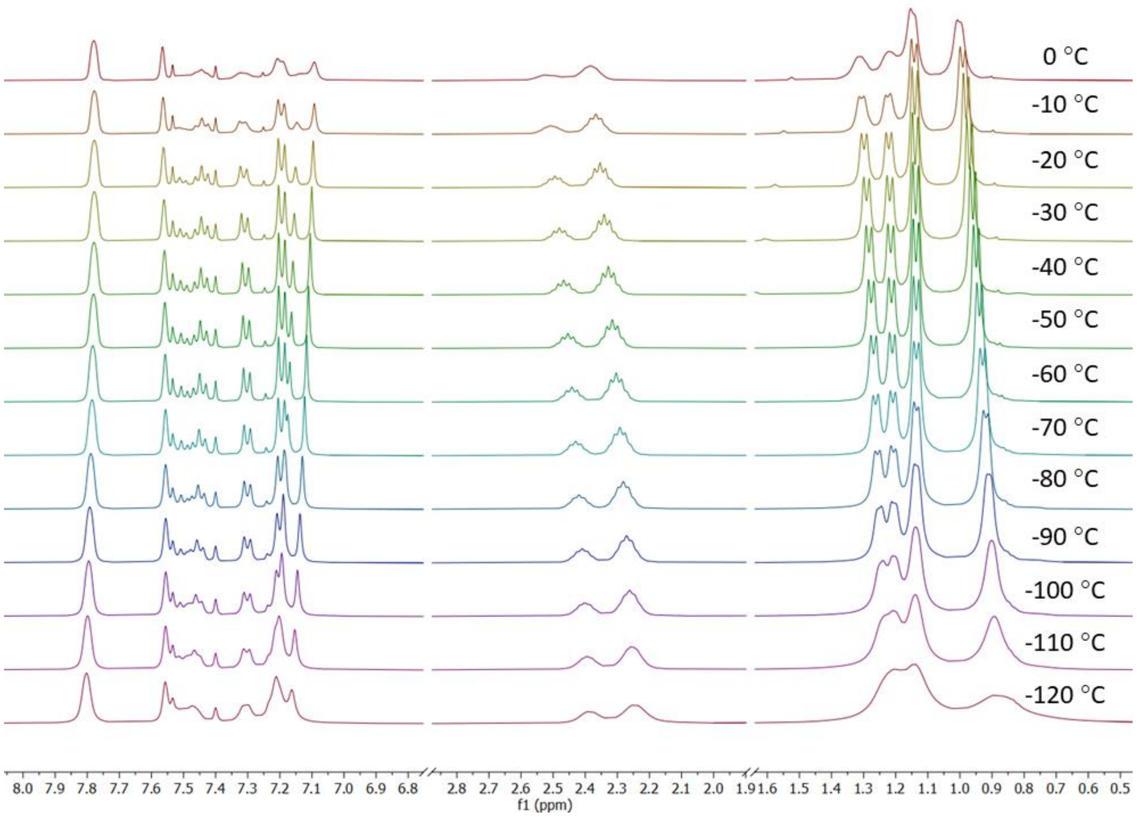

Figure S18. Expanded view of the variable-temperature ^{1}H NMR spectra (400 MHz, $CDCl_2F$) of complex **6·BAr^F$_{24}$**.

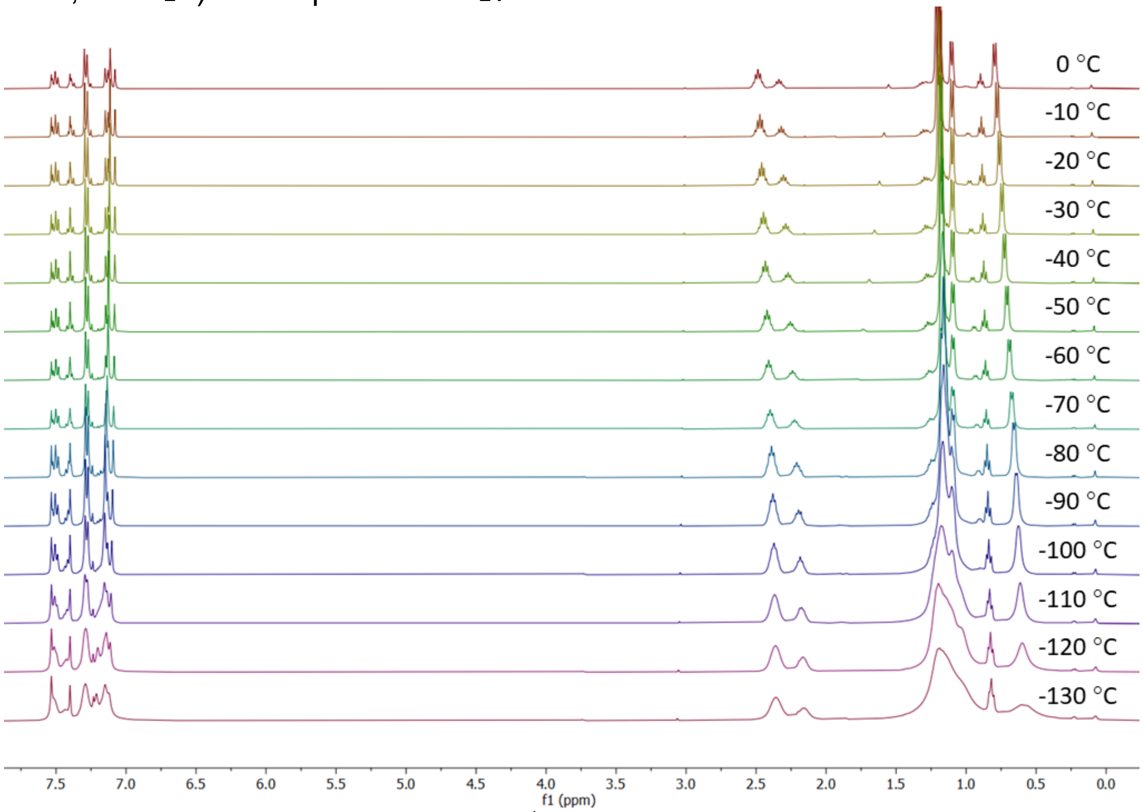

Figure S19. Variable-temperature ^{1}H NMR spectra (400 MHz, $CDCl_2F$) of complex **7**.

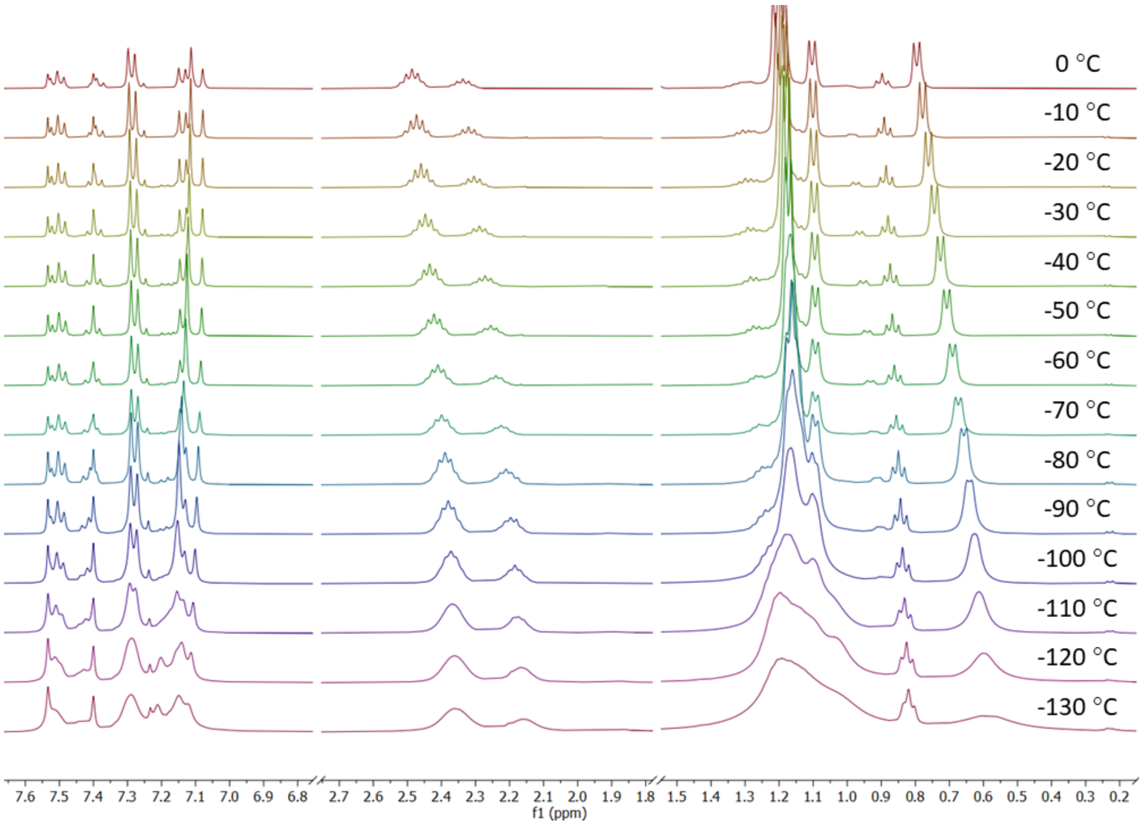

Figure S20. Expanded view of the variable-temperature ^{1}H NMR spectra (400 MHz, $CDCl_2F$) of complex **7**.

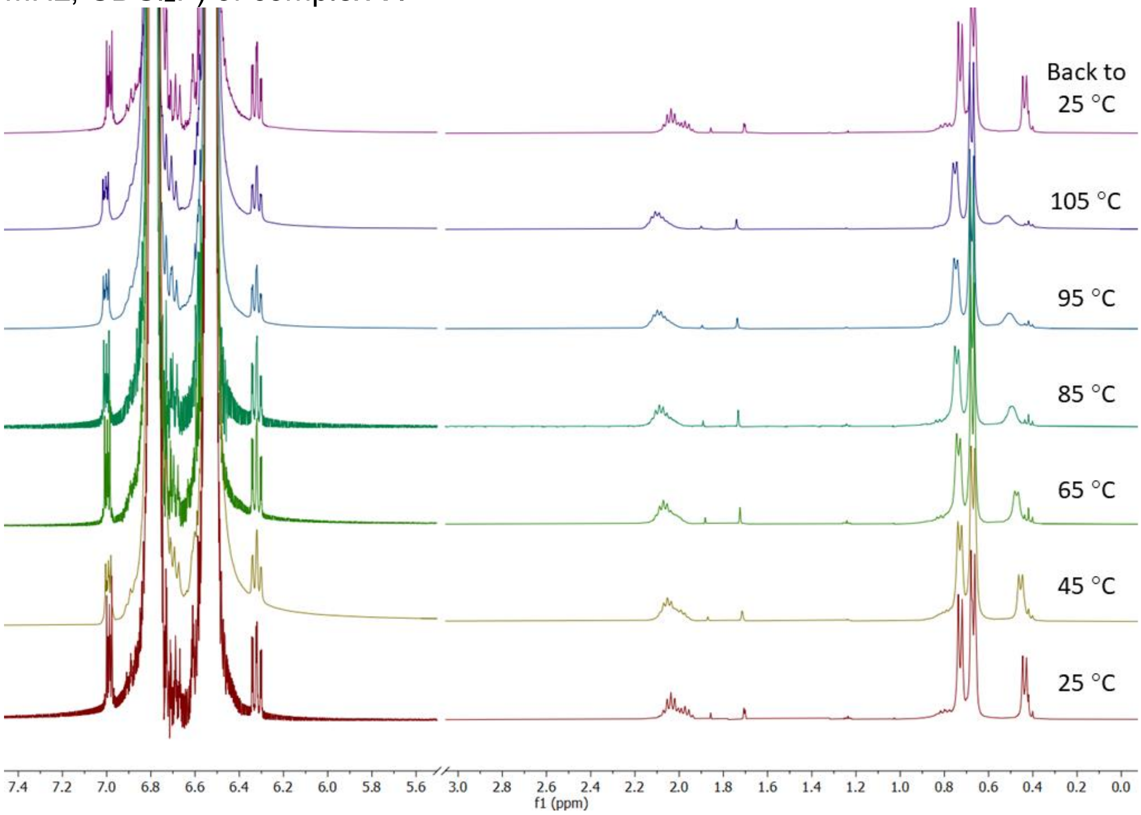

Figure S21. Variable-temperature ^{1}H NMR spectra (400 MHz, 1,2-dichlorobenzene) of complex **7**.

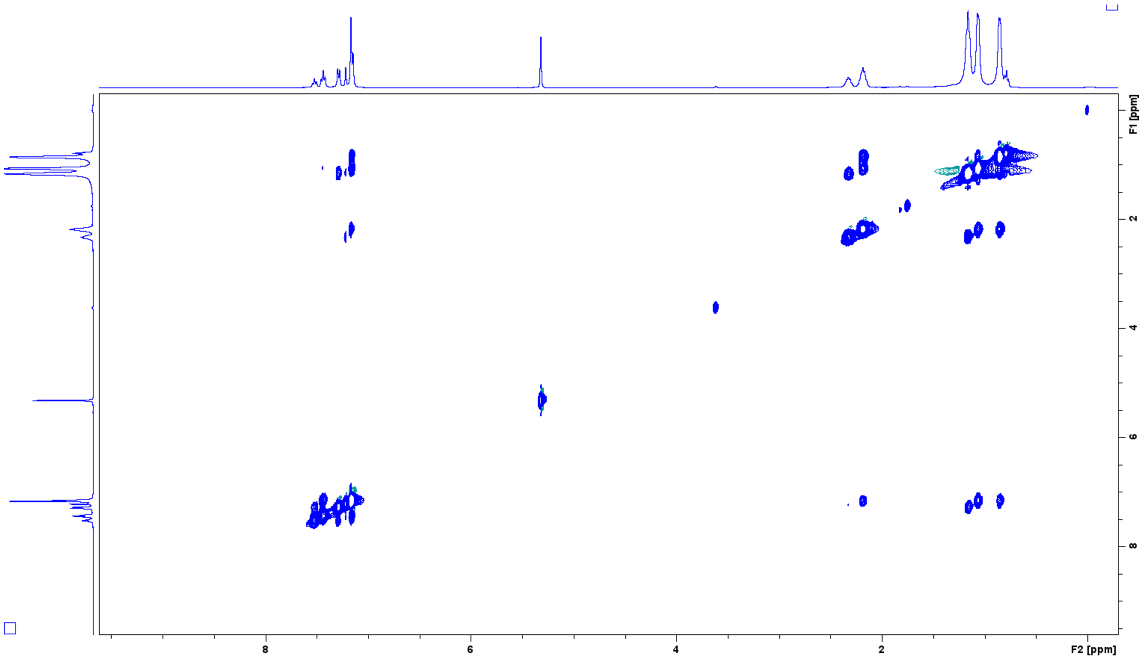

Figure S22. NOESY NMR spectrum (400 MHz, CD_2Cl_2, 193 K) of complex **6**. Under these conditions, NOE cross-peaks present a positive sign due to the molecular weight of the sample.

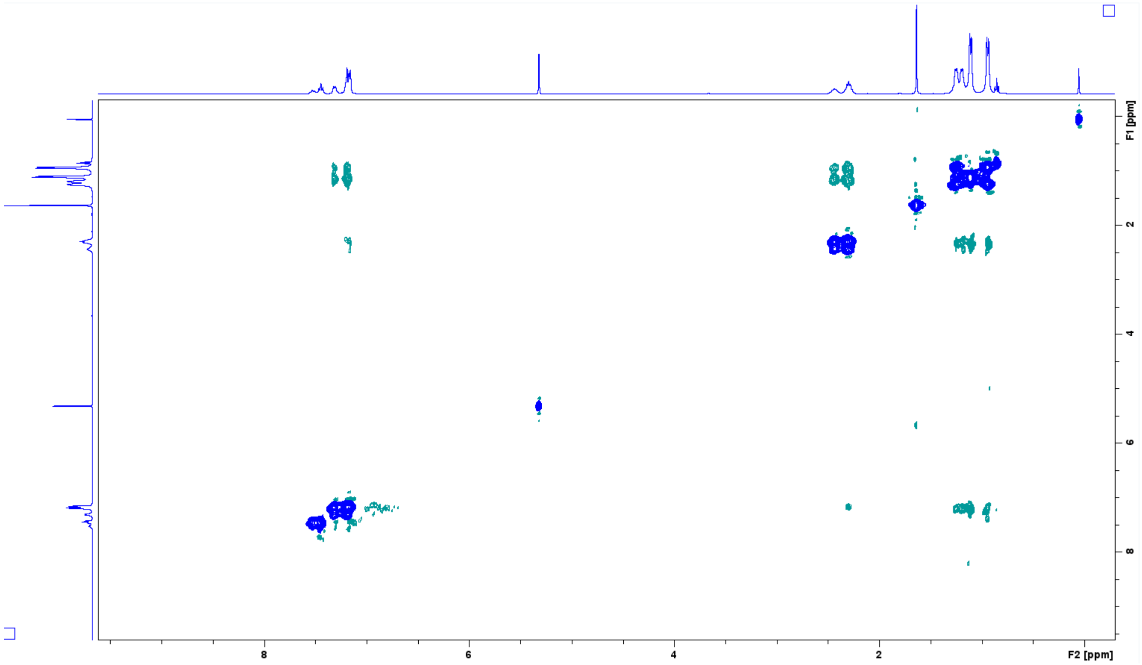

Figure S23. NOESY NMR spectrum (400 MHz, CD_2Cl_2, 263 K) of complex **6**. The temperature increase leads to a change of sign of the NOE cross-peaks, in agreement with a change in molecular motion and conformational averaging in large molecules.

Negative cross-peaks (green) are now due to cross-relaxation peaks (NOE contacts) and positive cross-peaks (blue) are due to exchange phenomena. In order to improve peak resolution, forward linear prediction (LPfr) was performed, with 48 coefficients (NCOEF) and 164 output points (LPBIN).

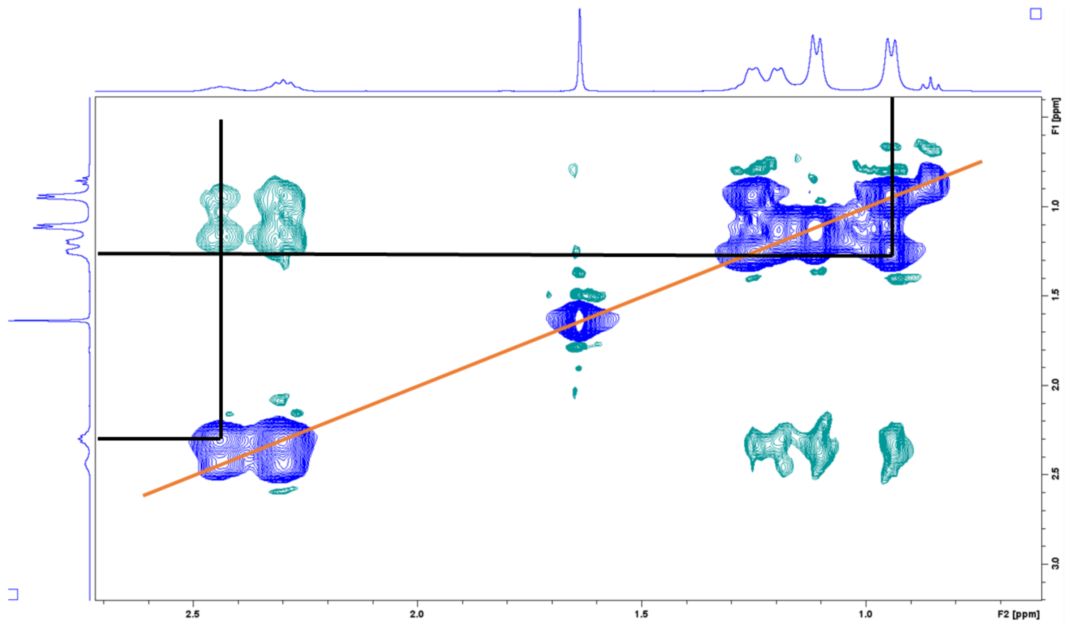

Figure S24. Expanded region of the NOESY NMR spectrum (400 MHz, CD_2Cl_2, 263 K) of complex **6**. Some exchange cross-peaks have been highlighted with black lines (diagonal as orange line).

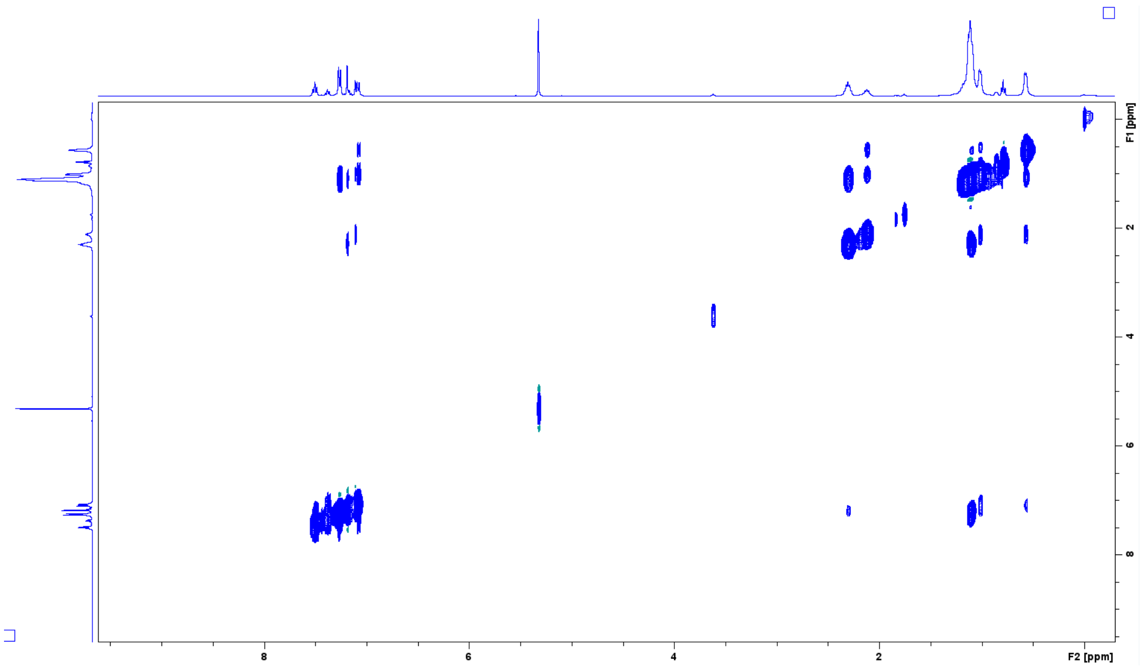

Figure S25. NOESY NMR spectrum (400 MHz, CD_2Cl_2, 193 K) of complex **7**. Under these conditions, NOE cross-peaks present a positive sign due to the molecular weight of the sample.

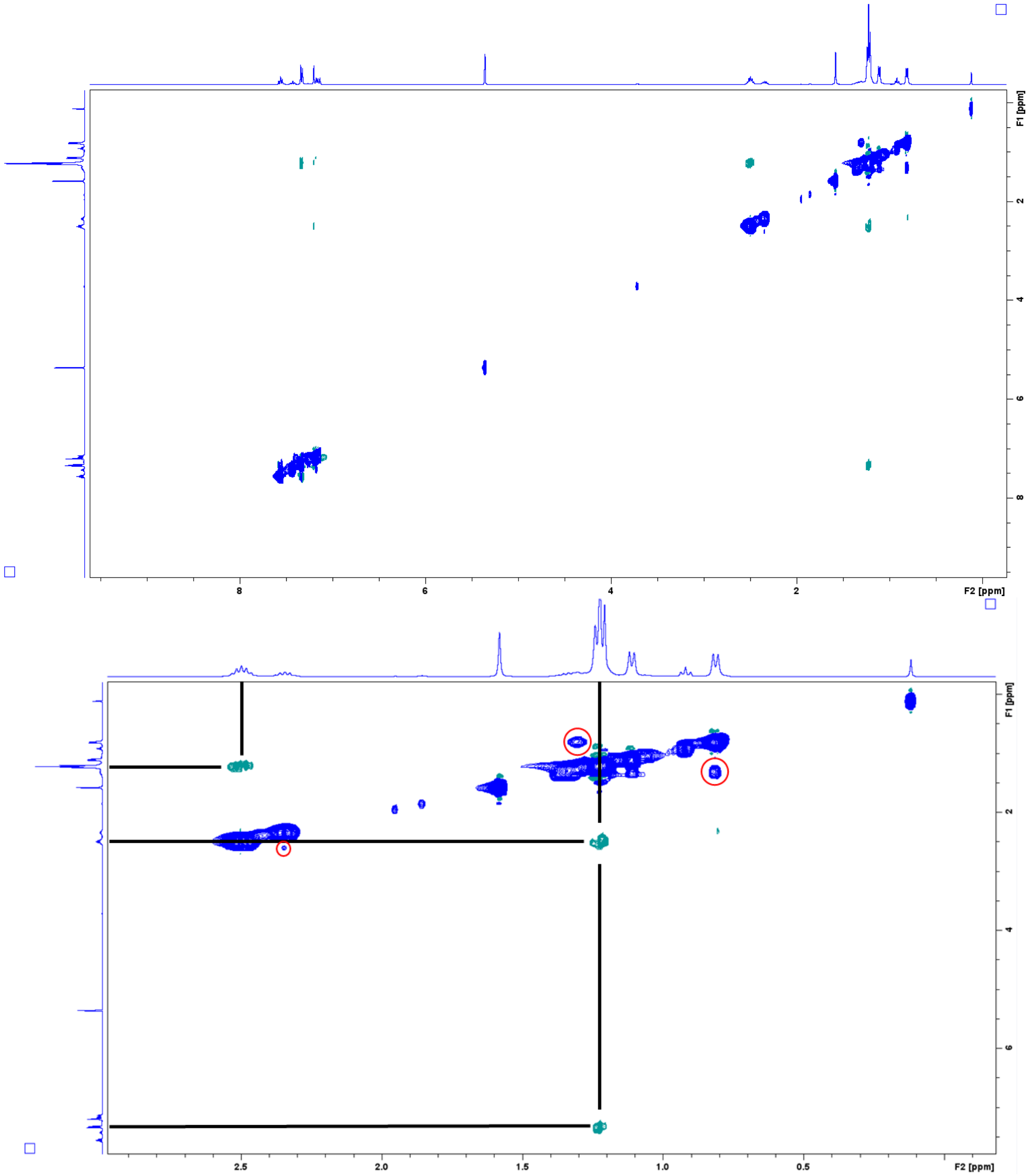

Figure S26. Top: NOESY NMR spectrum (400 MHz, CD_2Cl_2, 298 K) of complex **7**. The temperature increase leads to a change of sign of the NOE cross-peaks, in agreement with a change in molecular motion and conformational averaging in large molecules. **Bottom**: expanded region of the spectrum, highlighting some NOE contacts (artifacts like those due to *n*-pentane are shown as red circles).

4.1. Determination of the thermochemical parameters for the π,π exchange.

Extraction of the rate constants for the π,π-haptotropic shift in complex ***6*** *or* ***6·BAr^F$_{24}$*** *were calculated via the peak-to-peak separation of the signals attributed to the IPr methine protons (located between 2.0 and 2.5 ppm),[6] given their good resolution and absence of overlap with other resonances. In all cases, the peak-to-peak separation at -20 °C was assumed to correspond to the stopped-exchange limit.*

- *Expression utilized for the slow exchange regime:* $k = \frac{\pi}{\sqrt{2}}(\delta\nu^2 - \delta\nu_e^2)^{1/2}$ where δv is the peak separation in Hz in the stopped-exchange limit and $\delta\nu_e$ represents the peak separation in Hz during the exchange process.

- *Expression utilized for the coalescence temperature:* $k = \frac{\pi\delta\nu}{\sqrt{2}}$

Once the rate constants were calculated, linearization of the Eyring equation allows the determination of ΔH‡ and ΔS‡ associated with the process, and therefore, ΔG‡.

$$ln\,\frac{k}{T} = -\frac{\Delta H^{\ddagger}}{R}\cdot\frac{1}{T} + ln\frac{k_B}{h} + \frac{\Delta S^{\ddagger}}{R}$$

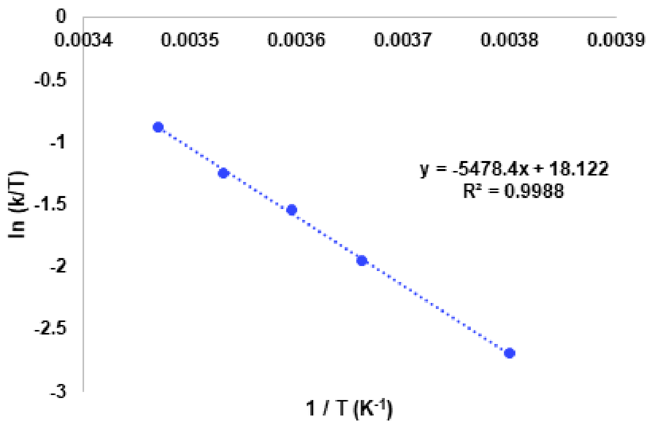

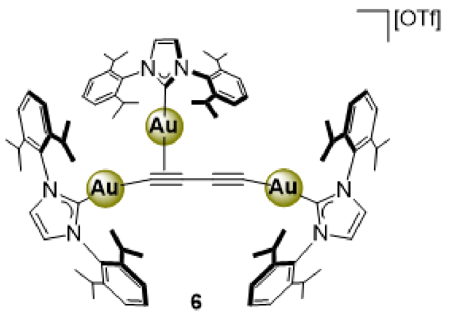

ΔH‡ = 10.9 ± 0.2 kcal mol-1
ΔS‡ = -11.2 ± 0.8 cal mol-1 K-1
ΔG‡ = 14.2 ± 0.2 kcal mol-1

Figure S27. Plot of ln (k/T) vs 1/T for complex **6** and thermochemical parameters for the proposed π,π exchange process. The k values were obtained from the experiment depicted in Figure S14.

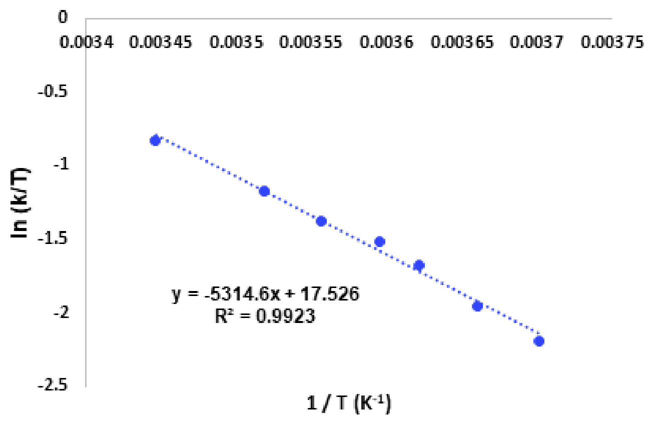

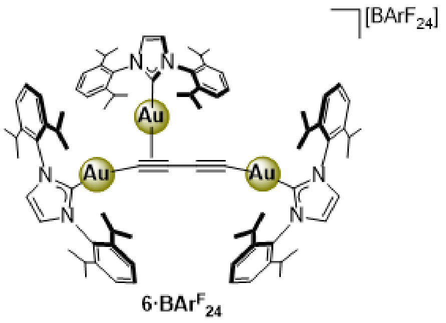

ΔH‡ = 10.6 ± 0.4 kcal mol-1
ΔS‡ = -12.4 ± 1.5 cal mol-1 K-1
ΔG‡ = 14.3 ± 0.4 kcal mol-1

Figure S28. Plot of ln (k/T) vs 1/T for complex **6·BAr^{F}24** and thermochemical parameters for the proposed π,π exchange process. The k values were obtained from the experiment depicted in Figure S16.

Alternatively, the Gibbs free energy can be determined through the coalescence temperature (T_c), as expressed in $\Delta G^{\ddagger} = RT_c[22.96 + ln\left(\frac{T_c}{\Delta\nu}\right)]$[7]

Assuming different coalescence temperatures, the following values were obtained:

Complex **6**		Complex **6·BAr$^F_{24}$**	
T_c / °C	ΔG$^{\pm}$ / kcal mol^{-1}	T_c / °C	ΔG$^{\pm}$ / kcal mol^{-1}
10	13.9	10	13.8
15	14.1	15	14.1
20	14.4	20	14.3

Table S1. Energy barrier for the proposed π,π-haptotropic shift considering different coalescence temperatures. The observed ΔG$^{\pm}$ values are almost identical to those obtained by means of the peak-to-peak separation method described above.

5. Vibrational spectra

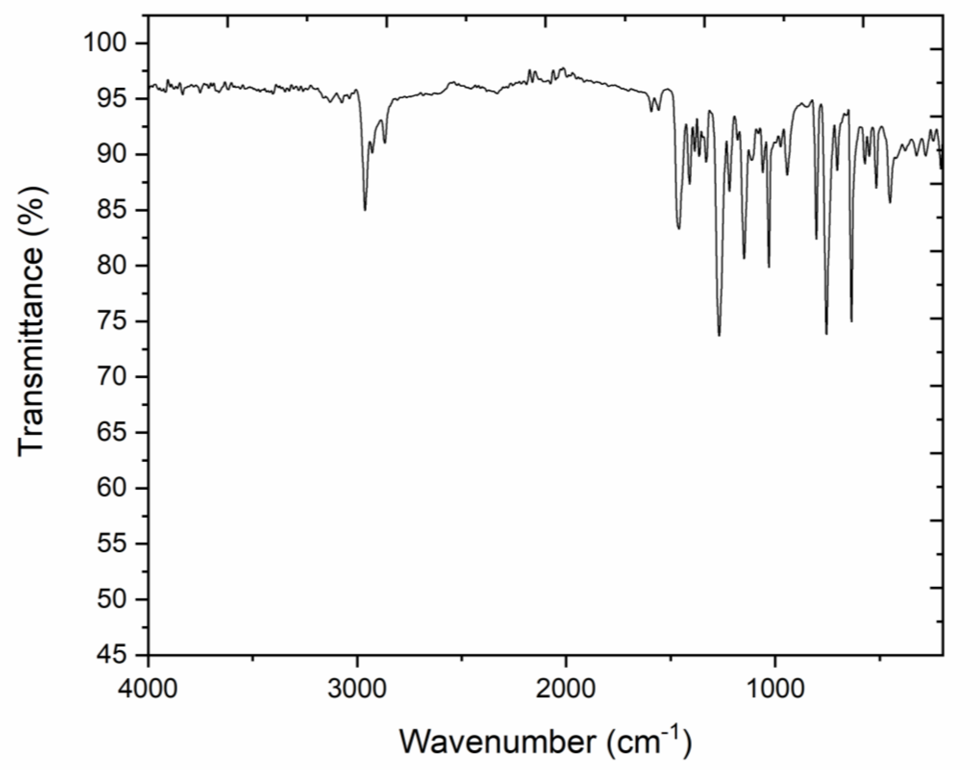

Figure S29. IR spectrum of complex **3**.

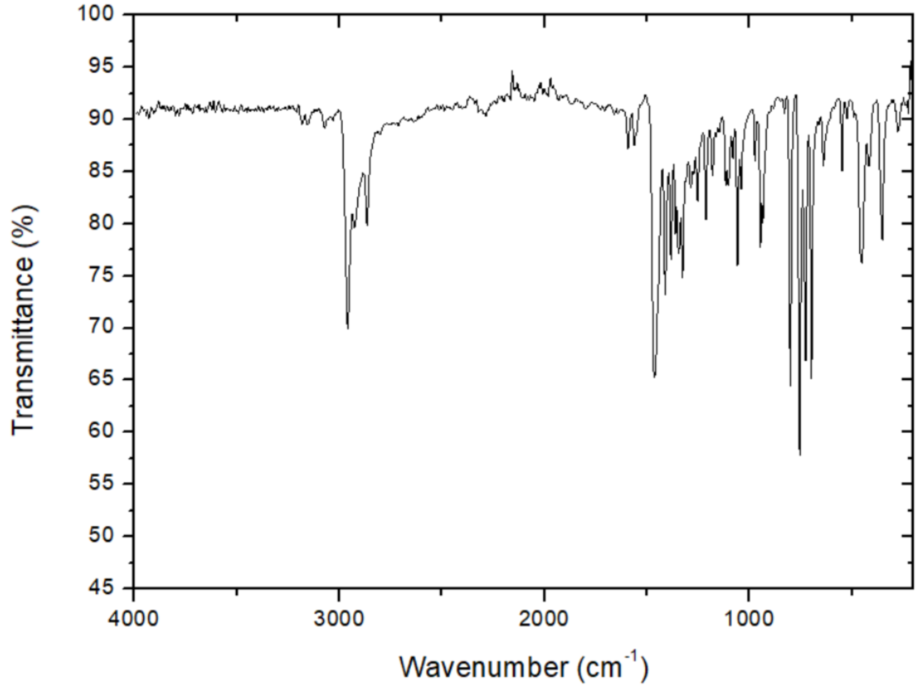

Figure S30. IR spectrum of complex **5**.

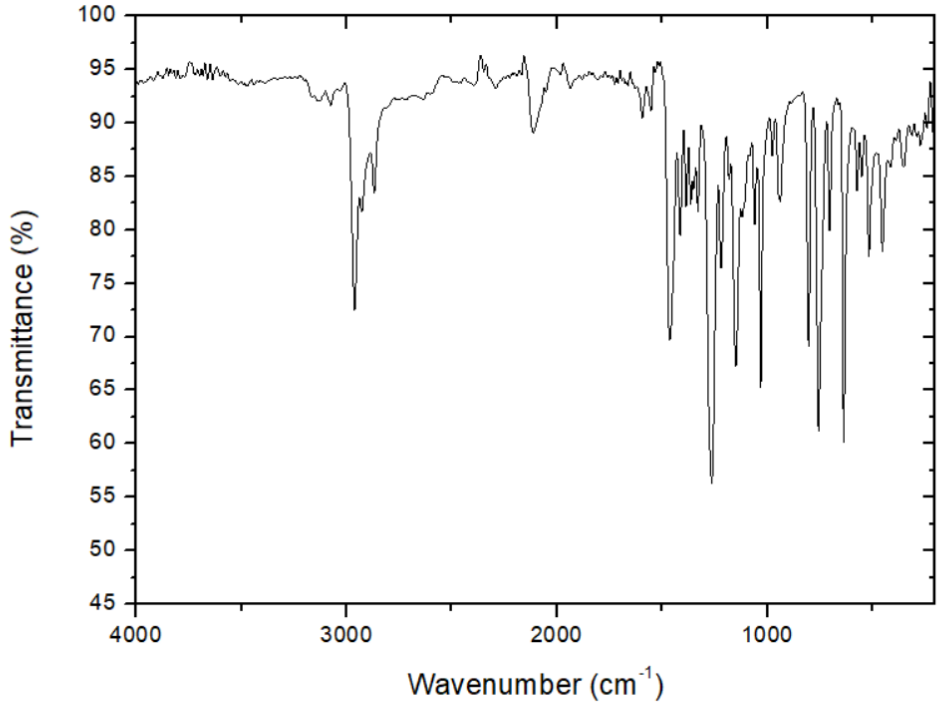

Figure S31. IR spectrum of complex **6**.

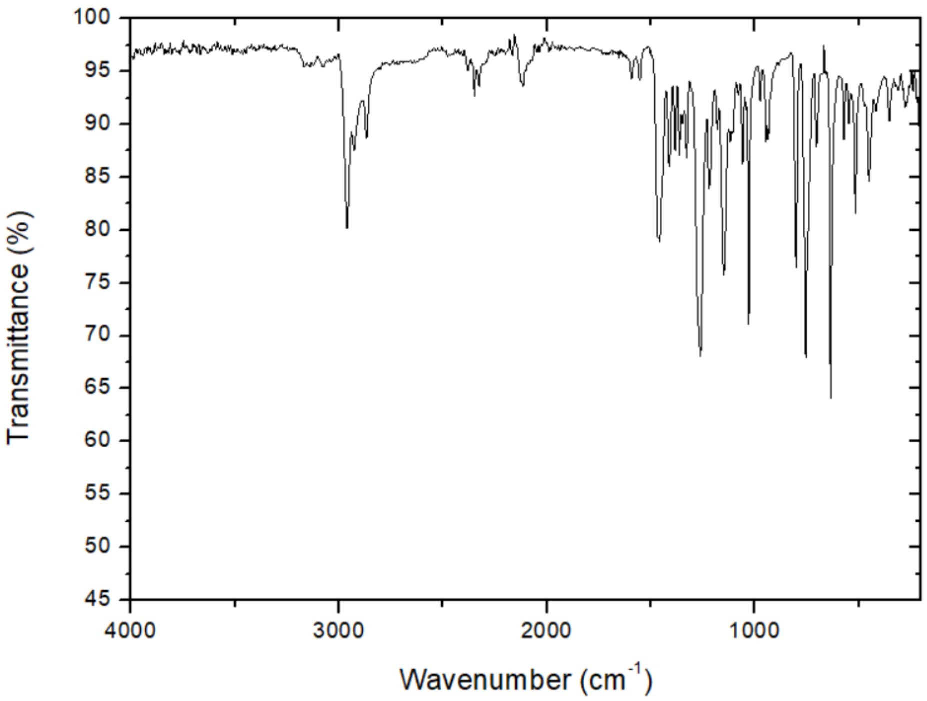

Figure S32. IR spectrum of complex **7**.

6. X-ray crystallography

- Complex **3**

Low-temperature diffraction data were collected on a Bruker D8 Quest APEX-III single crystal diffractometer with a Photon III detector and a IμS 3.0 microfocus X-ray source at the Instituto de Investigaciones Químicas, Sevilla. Data were collected by means of ω and φ scans using monochromatic radiation λ(Mo Kα1) = 0.71073 Å. The diffraction images collected were processed and scaled using APEX4 v2021.10-0 software. The structures were solved with SHELXT and was refined against F2 on all data by full-matrix least squares with SHELXL,[8] using Olex2 as graphical interface.[9] All non-hydrogen atoms were refined anisotropically. Hydrogen atoms were included in the model at geometrically calculated positions and refined using a riding model, unless otherwise noted. The unit cell contains four hexane molecules which were treated as a diffuse contribution to the overall scattering without specific atom positions by SQUEEZE.[10] The isotropic displacement parameters of all hydrogen atoms were fixed to 1.2 times the U value of the atoms to which they are linked (1.5 times for methyl groups).

Crystal Data for $C_{102}H_{146}Au_{2.291}Cu_{0.709}F_3N_6O_6S$ (M =2137.60 g/mol): monoclinic, space group P2$_1$/n (no. 14), a = 15.9067(9) Å, b = 30.7177(17) Å, c = 21.9446(13) Å, β = 96.494(2)°, V = 10653.7(11) Å^3, Z = 4, T = 193.00 K, μ(MoKα) = 3.362 mm^{-1}, $Dcalc$ = 1.333 g/cm^3, 153388 reflections measured (3.964° ≤ 2Θ ≤ 50.86°), 19623 unique (R_{int} = 0.1025, R_{sigma} = 0.0622) which were used in all calculations. The final R_1 was 0.0575 (I > 2σ(I)) and wR_2 was 0.1109 (all data).

Table S2. Crystal data and structure refinement for **3.**

Identification code	pr0425a
Empirical formula	$C_{102}H_{146}Au_{2.29}Cu_{0.71}F_3N_6O_6S$
Formula weight	2137.60
Temperature/K	193.00
Crystal system	monoclinic
Space group	$P2_1/n$
a/Å	15.9067(9)
b/Å	30.7177(17)
c/Å	21.9446(13)
α/°	90
β/°	96.494(2)
γ/°	90
Volume/Å^3	10653.7(11)
Z	4
ρ_{calc}g/cm^3	1.333
μ/mm^{-1}	3.362
F(000)	4370.0
Crystal size/mm^3	0.05 × 0.03 × 0.01
Radiation	MoKα (λ = 0.71073)
2Θ range for data collection/°	3.964 to 50.86
Index ranges	-19 ≤ h ≤ 19, -37 ≤ k ≤ 37, -26 ≤ l ≤ 26
Reflections collected	153388
Independent reflections	19623 [R_{int} = 0.1025, R_{sigma} = 0.0622]
Data/restraints/parameters	19623/273/1070
Goodness-of-fit on F^2	1.076
Final R indexes [I>=2σ (I)]	R_1 = 0.0575, wR_2 = 0.0996
Final R indexes [all data]	R_1 = 0.0901, wR_2 = 0.1109
Largest diff. peak/hole / e Å^{-3}	1.13/-1.04

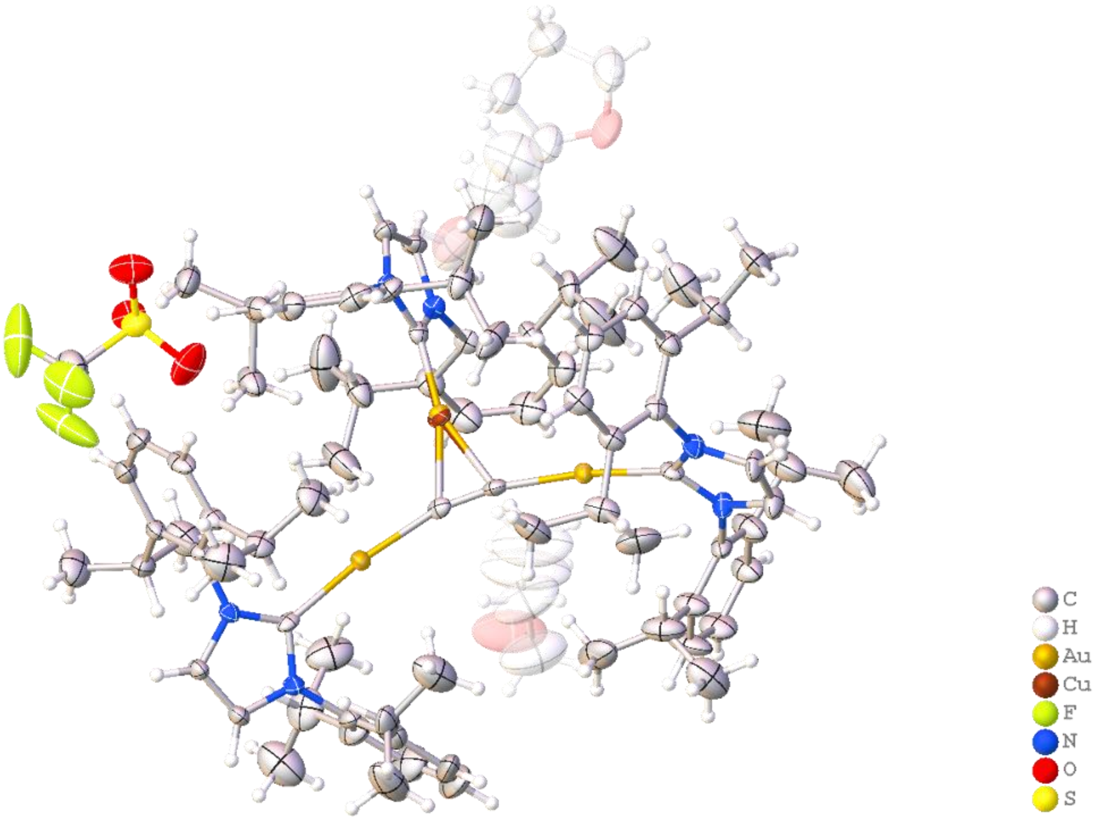

Figure S33. Solid-state structure of complex **3** at the 50% probability level.

- Complex **5**

Low-temperature diffraction data were collected on a Bruker D8 Quest APEX-III single crystal diffractometer with a Photon III detector and a IμS 3.0 microfocus X-ray source at the Instituto de Investigaciones Químicas, Sevilla. Data were collected by means of ω and φ scans using monochromatic radiation λ(Mo Kα1) = 0.71073 Å. The diffraction images collected were processed and scaled using APEX-4 v2021.4-0 software. The structures were solved with SHELXT and was refined against F2 on all data by full-matrix least squares with SHELXL,[8] using Olex2 as graphical interface.[9] All non-hydrogen atoms were refined anisotropically. Hydrogen atoms were included in the model at geometrically calculated positions and refined using a riding model, unless otherwise noted. The isotropic displacement parameters of all hydrogen atoms were fixed to 1.2 times the U value of the atoms to which they are linked (1.5 times for methyl groups).

Crystal Data for $C_{58}H_{72}Au_2N_4$ (M =1219.12 g/mol): orthorhombic, space group $P2_12_12_1$ (no. 19), a = 11.6545(4) Å, b = 16.5851(6) Å, c = 28.1410(9) Å, V = 5439.4(3) Å^3, Z = 4, T = 193.00 K, μ(MoKα) = 5.426 mm^{-1}, $Dcalc$ = 1.489 g/cm^3, 56771 reflections measured (3.796° ≤ 2Θ ≤ 56.654°), 13493 unique (R_{int} = 0.0618, R_{sigma} = 0.0557) which were used in all calculations. The final R_1 was 0.0336 (I > 2σ(I)) and wR_2 was 0.0646 (all data).

Table S3. Crystal data and structure refinement for **5.**

Identification code	pr1424a

Empirical formula	$C_{58}H_{72}Au_2N_4$
Formula weight	1219.12
Temperature/K	193.00
Crystal system	orthorhombic
Space group	$P2_12_12_1$
a/Å	11.6545(4)
b/Å	16.5851(6)
c/Å	28.1410(9)
α/°	90
β/°	90
γ/°	90
Volume/Å^3	5439.4(3)
Z	4
ρ_{calc}g/cm^3	1.489
μ/mm^{-1}	5.426
F(000)	2424.0
Crystal size/mm^3	0.35 × 0.06 × 0.05
Radiation	MoKα (λ = 0.71073)
2Θ range for data collection/°	3.796 to 56.654
Index ranges	-15 ≤ h ≤ 15, -22 ≤ k ≤ 22, -37 ≤ l ≤ 36
Reflections collected	56771
Independent reflections	13493 [R_{int} = 0.0618, R_{sigma} = 0.0557]
Data/restraints/parameters	13493/2/615
Goodness-of-fit on F^2	1.039
Final R indexes [I>=2σ (I)]	R_1 = 0.0336, wR_2 = 0.0614
Final R indexes [all data]	R_1 = 0.0439, wR_2 = 0.0646
Largest diff. peak/hole / e Å^{-3}	1.15/-1.10
Flack parameter	0.494(9)

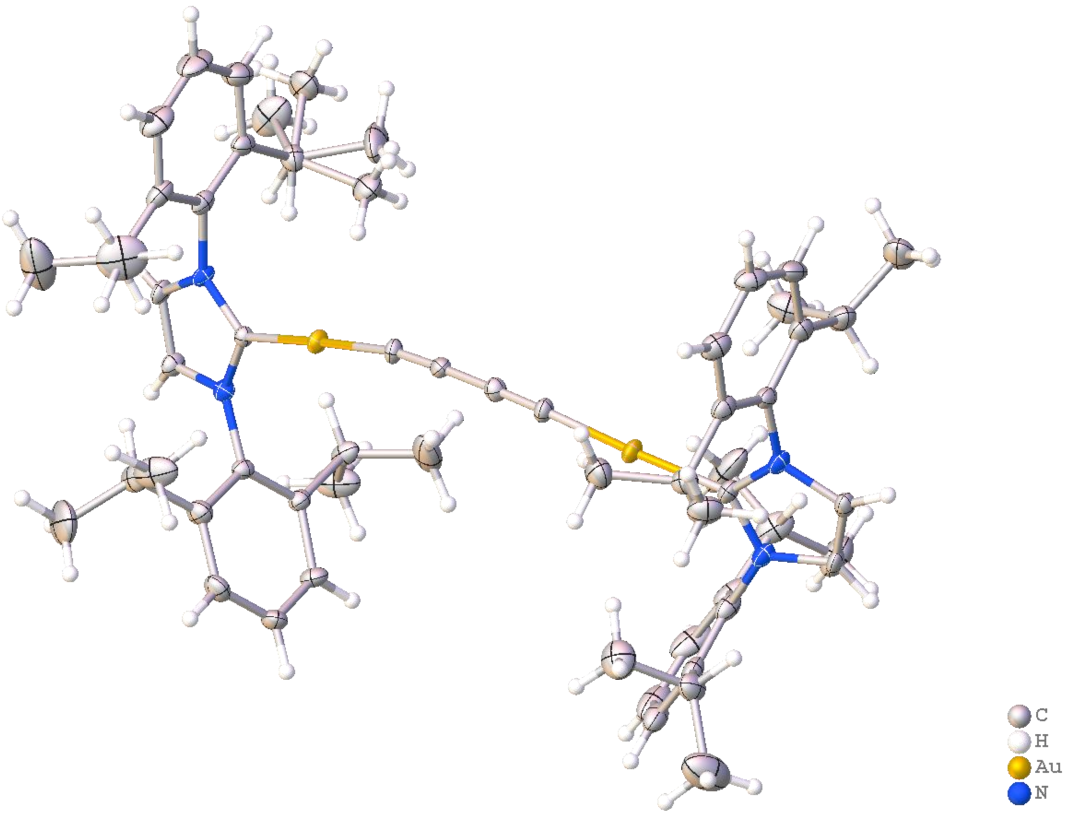

Figure S34. Solid-state structure of complex **5** at the 50% probability level.

- Complex **6**

Low-temperature diffraction data were collected on a Bruker D8 Quest APEX-III single crystal diffractometer with a Photon III detector and a IμS 3.0 microfocus X-ray source at the Instituto de Investigaciones Químicas, Sevilla. Data were collected by means of ω and φ scans using monochromatic radiation λ(Mo Kα1) = 0.71073 Å. The diffraction images collected were processed and scaled using APEX4 v2021.10-0 software. The structures were solved with SHELXT and was refined against F2 on all data by full-matrix least squares with SHELXL,[8] using Olex2 as graphical interface.[9] All non-hydrogen atoms were refined anisotropically. Hydrogen atoms were included in the model at geometrically calculated positions and refined using a riding model, unless otherwise noted. The unit cell contains one tetrahydrofuran molecule which was treated as a diffuse contribution to the overall scattering without specific atom positions by SQUEEZE.[10] The isotropic displacement parameters of all hydrogen atoms were fixed to 1.2 times the U value of the atoms to which they are linked (1.5 times for methyl groups). Some SIMU and DELU restrains were used in order to model the isopropyl groups. The crystal exhibited some twinning, so the structure was refined using HKLF 5 and BASF, allowing to improve it.

Crystal Data for $C_{88}H_{112}Au_3F_3N_6O_{3.5}S$ (M =1989.79 g/mol): triclinic, space group P-1 (no. 2), a = 16.9528(11) Å, b = 18.7747(14) Å, c = 18.7843(14) Å, α = 118.108(3)°, β = 99.956(3)°, γ = 104.331(3)°, V = 4807.4(6) Å^3, Z = 2, T = 193.0 K, μ(MoKα) = 4.639 mm^{-1}, $Dcalc$ = 1.375 g/cm^3, 40480 reflections measured (4.282° ≤ 2Θ ≤ 50.926°), 40480 unique (R_{int} = ?, R_{sigma} = 0.0697) which

were used in all calculations. The final R_1 was 0.0626 ($I > 2\sigma(I)$) and wR_2 was 0.1573 (all data).

Table S4. Crystal data and structure refinement for **6.**

Identification code	pr2024a
Empirical formula	$C_{88}H_{112}Au_3F_3N_6O_{3.5}S$
Formula weight	1989.79
Temperature/K	193.0
Crystal system	triclinic
Space group	P-1
a/Å	16.9528(11)
b/Å	18.7747(14)
c/Å	18.7843(14)
α/°	118.108(3)
β/°	99.956(3)
γ/°	104.331(3)
Volume/Å^3	4807.4(6)
Z	2
ρ_{calc}g/cm^3	1.375
μ/mm^{-1}	4.639
F(000)	1980.0
Crystal size/mm^3	0.2 × 0.1 × 0.05
Radiation	MoKα (λ = 0.71073)
2Θ range for data collection/°	4.282 to 50.926
Index ranges	-19 ≤ h ≤ 20, -22 ≤ k ≤ 0, -20 ≤ l ≤ 22
Reflections collected	40480
Independent reflections	40480 [R_{int} = ?, R_{sigma} = 0.0697]
Data/restraints/parameters	40480/63/944
Goodness-of-fit on F^2	1.037
Final R indexes [I>=2σ (I)]	R_1 = 0.0626, wR_2 = 0.1298
Final R indexes [all data]	R_1 = 0.1033, wR_2 = 0.1573
Largest diff. peak/hole / e Å^{-3}	3.53/-1.60

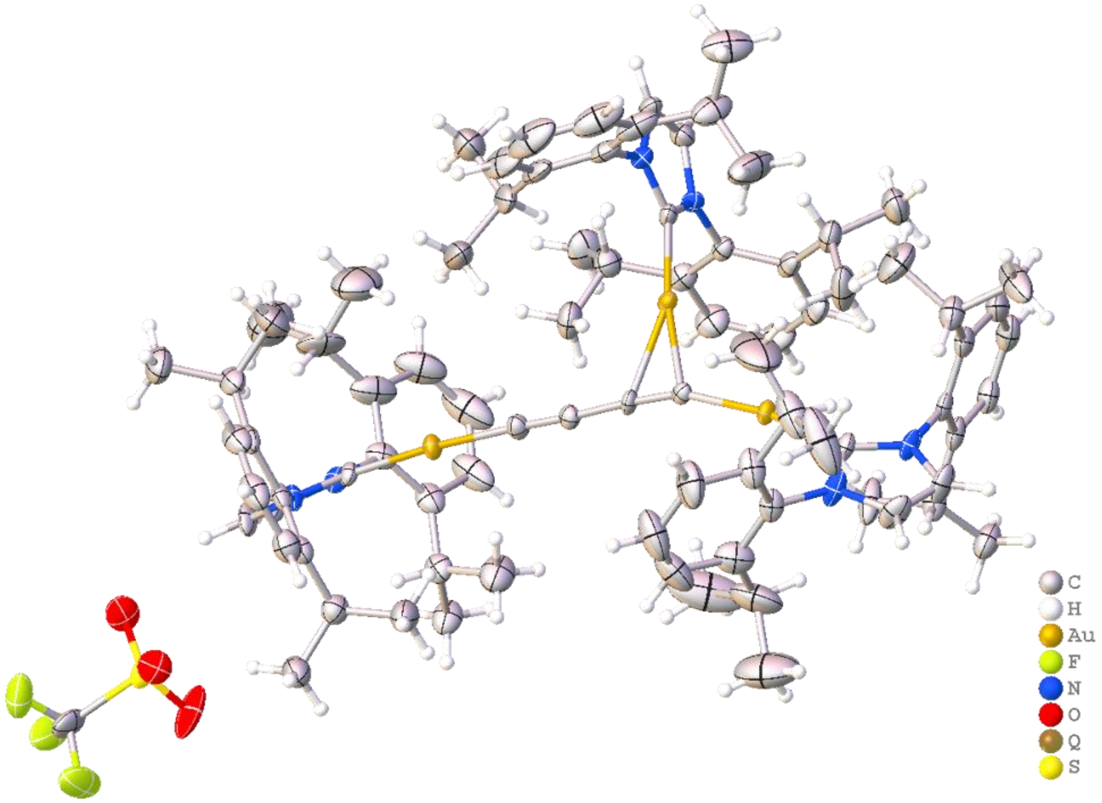

Figure S35. Solid-state structure of complex **6** at the 50% probability level.

- Complex **7**

Low-temperature diffraction data were collected on a Bruker D8 Quest APEX-III single crystal diffractometer with a Photon III detector and a IμS 3.0 microfocus X-ray source at the Instituto de Investigaciones Químicas, Sevilla. Data were collected by means of ω and φ scans using monochromatic radiation λ(Mo Kα1) = 0.71073 Å. The diffraction images collected were processed and scaled using APEX4 v2021.10-0 software. The structures were solved with SHELXT and was refined against F2 on all data by full-matrix least squares with SHELXL,[8] using Olex2 as graphical interface.[9] All non-hydrogen atoms were refined anisotropically. Hydrogen atoms were included in the model at geometrically calculated positions and refined using a riding model, unless otherwise noted. The unit cell contains 3.75 tetrahydrofuran molecules which were treated as a diffuse contribution to the overall scattering without specific atom positions by SQUEEZE.[10] The isotropic displacement parameters of all hydrogen atoms were fixed to 1.2 times the U value of the atoms to which they are linked (1.5 times for methyl groups). Some SIMU and DELU restrains were used in order to model the isopropyl groups

Crystal Data for $C_{89.75}H_{115.5}Au_2CuF_3N_6O_{3.94}S$ (M =1887.95 g/mol): triclinic, space group P-1 (no. 2), a = 19.825(2) Å, b = 20.661(4) Å, c = 23.113(4) Å, α = 91.696(3)°, β = 95.725(3)°, γ = 90.886(4)°, V = 9414(3) Å^3, Z = 4, T = 193.00 K, μ(MoKα) = 3.408 mm^{-1}, $Dcalc$ = 1.332 g/cm^3, 220122 reflections measured (3.918° ≤ 2Θ ≤ 51.364°), 35754 unique (R_{int} = 0.0988, R_{sigma} = 0.0633) which

were used in all calculations. The final R_1 was 0.0393 (I > 2σ(I)) and wR_2 was 0.0900 (all data).

Table S5. Crystal data and structure refinement for **7.**

Identification code	pr1724b
Empirical formula	$C_{89.75}H_{115.5}Au_2CuF_3N_6O_{3.94}S$
Formula weight	1887.95
Temperature/K	193.00
Crystal system	triclinic
Space group	P-1
a/Å	19.825(2)
b/Å	20.661(4)
c/Å	23.113(4)
α/°	91.696(3)
β/°	95.725(3)
γ/°	90.886(4)
Volume/Å^3	9414(3)
Z	4
ρ_{calc}g/cm^3	1.332
μ/mm^{-1}	3.408
F(000)	3830.0
Crystal size/mm^3	0.3 × 0.3 × 0.13
Radiation	MoKα (λ = 0.71073)
2Θ range for data collection/°	3.918 to 51.364
Index ranges	-24 ≤ h ≤ 24, -25 ≤ k ≤ 25, -28 ≤ l ≤ 28
Reflections collected	220122
Independent reflections	35754 [R_{int} = 0.0988, R_{sigma} = 0.0633]
Data/restraints/parameters	35754/63/1885
Goodness-of-fit on F^2	0.999
Final R indexes [I>=2σ (I)]	R_1 = 0.0393, wR_2 = 0.0767
Final R indexes [all data]	R_1 = 0.0719, wR_2 = 0.0900
Largest diff. peak/hole / e Å^{-3}	1.13/-1.26

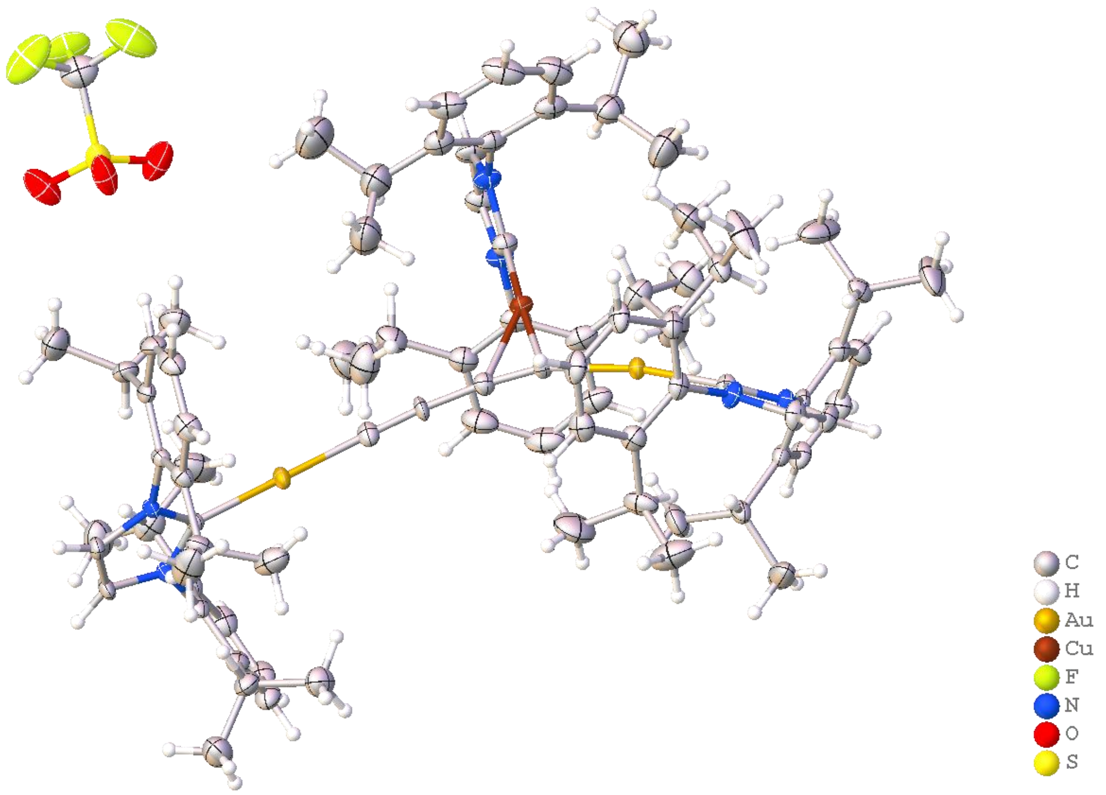

Figure S36. Solid-state structure of complex **7** at the 50% probability level.

7. Computational Details

Calculations were performed using the PBE0 functional[11] including the D3 version of Grimme's dispersion correctionand Becke-Johnson damping function[12] (PBE0-D3BJ) as implemented in Gaussian 09.[13] Geometry optimizations were performed in solution (solvent = dichloromethane, ε = 8.93) using the continuum SMD model[14] and basis set 1 (BS1). BS1 uses the double-ζ 6-31G(d,p)[15] basis set for the H, C, N and atoms and the scalar relativistic Stuttgart−Dresden SDD pseudopotential[16] and its associated double-ζ basis set, complemented with a set of polarization functions, for the Au and Cu atoms.[17] The nature of the stationary points was confirmed by frequency analysis. Connections between the transition states and the minima were checked by perturbing the transition state geometry along the TS coordinate and optimizing until the corresponding minima. All energies in solution were corrected by single-point calculations with the larger basis set 2 (BS2) including triple-ζ def2TZVP basis set for the H, C, N, Cu and Au atoms.[18] The scalar relativistic Stuttgart−Dresden SDD pseudopotential and its associated basis set for Cu and Au was used in the energy profile calculations. Gibbs energies in benzene were calculated at 298.15 K. Gibbs energy corrections were obtained based on vibrational frequencies of the BS1- optimized structures using the quasi-harmonic approximation. Thermal contributions to the Gibbs energies were corrected by employing the approximation described by Grimme, where entropic terms for frequencies below a cut-off of 100 cm-1 were calculated using the free-rotor approximation.[19] The GoodVibes program developed by Paton and Funes-Ardoiz was employed to introduce these corrections.[20] All reported energies in

the main text and section 8 of this document correspond to PBE0-D3BJ/BS2 Gibbs energies in dichloromethane solvent (1 M) at 298.15 K in kcal mol^{-1}. Structure visualization was performed with Chemcraft software.[21]

Energy Decomposition Analysis (EDA)

Within the EDA method,[22] the interaction energy can be further decomposed into the following chemically meaningful terms:

$$\Delta E_{int}(\zeta) = \Delta V_{elstat}(\zeta) + \Delta E_{Pauli}(\zeta) + \Delta E_{orb}(\zeta) + \Delta E_{disp}(\zeta)$$

The term ΔV_{elstat} corresponds to the classical electrostatic interaction between the unperturbed charge distributions of the deformed reactants and is usually attractive. The Pauli repulsion ΔE_{Pauli} comprises the destabilizing interactions between occupied orbitals and is responsible for any steric repulsion. The orbital interaction ΔE_{orb} accounts for bond pair formation, charge transfer (interaction between occupied orbitals on one moiety with unoccupied orbitals on the other, including HOMO-LUMO interactions), and polarization (empty-occupied orbital mixing on one fragment due to the presence of another fragment). Finally, the ΔE_{disp} term takes into account interactions coming from dispersion forces. Moreover, the NOCV (Natural Orbital for Chemical Valence)[23] extension of the EDA method has been also used to further partition the ΔE_{orb} term. The EDA-NOCV approach provides pairwise energy contributions for each pair of interacting orbitals to the total bond energy.

The program package ADF[24] was used the EDA(NOCV) calculations using the optimized SMD-PBE(0)-D3BJ/6-31G(d,p)&SDD geometries at the same PBE(0)-D3BJ level in conjunction with a double-ζ-quality basis set using uncontracted Slater-type orbitals (STOs) augmented by polarization functions.[25] Auxiliary sets of s, p, d, f, and g STOs were used to fit the molecular densities and to represent the Coulomb and exchange potentials accurately in each SCF cycle.[26] Scalar relativistic effects were incorporated by applying the zeroth-order regular approximation (ZORA).[27] This level of theory is denoted ZORA-PBE(0)-D3BJ/DZP//SMD-PBE(0)-D3BJ/6-31G(d,p)&SDD.

8. Calculated energy values of the exchange processes

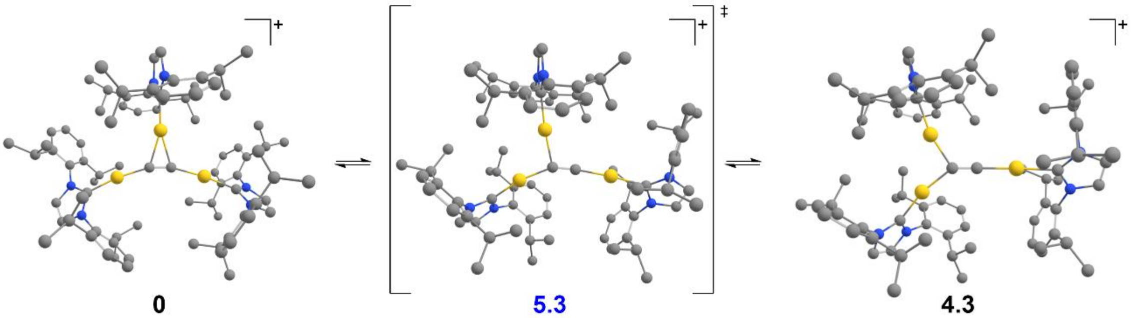

0 Complex **1**

5.3 **TS 1σ,π**

4.3 Complex **1-*gem***

Figure S37. Calculated relative Gibbs free energies for the σ,π exchange process in complex **1**. H atoms have been omitted for clarity.

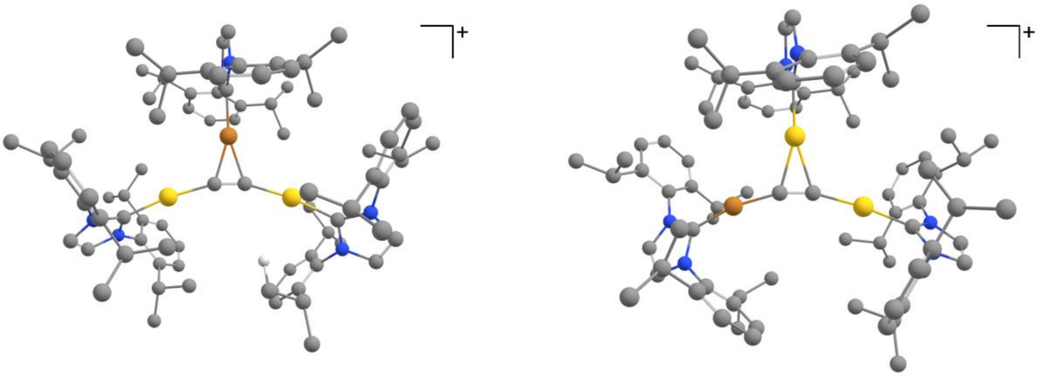

0 Complex **3**

8.9 Complex **3'**

Figure S38. Calculated relative Gibbs free energies for **3** and **3'**. H atoms have been omitted for clarity.

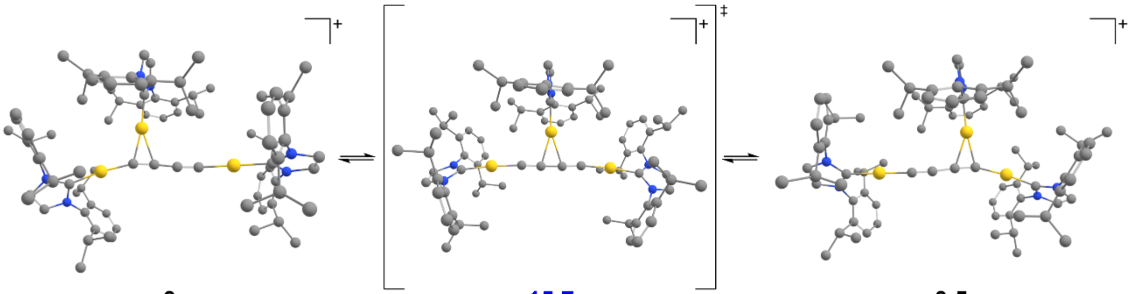

0 Complex **6**

15.7 **TS 6 π,π**

0.5 Complex **6-fw**

Figure S39. Calculated relative Gibbs free energies for the π,π exchange process in complex **6**. H atoms have been omitted for clarity.

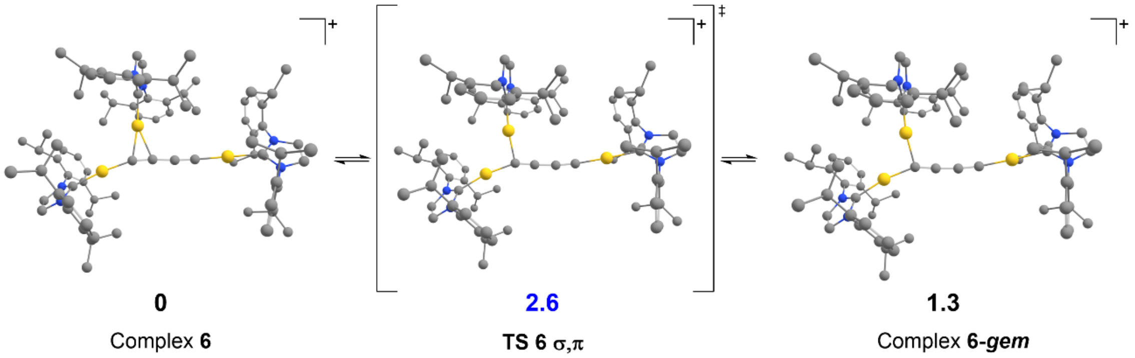

Figure S40. Calculated relative Gibbs free energies for the σ,π exchange process in complex **6**. H atoms have been omitted for clarity.

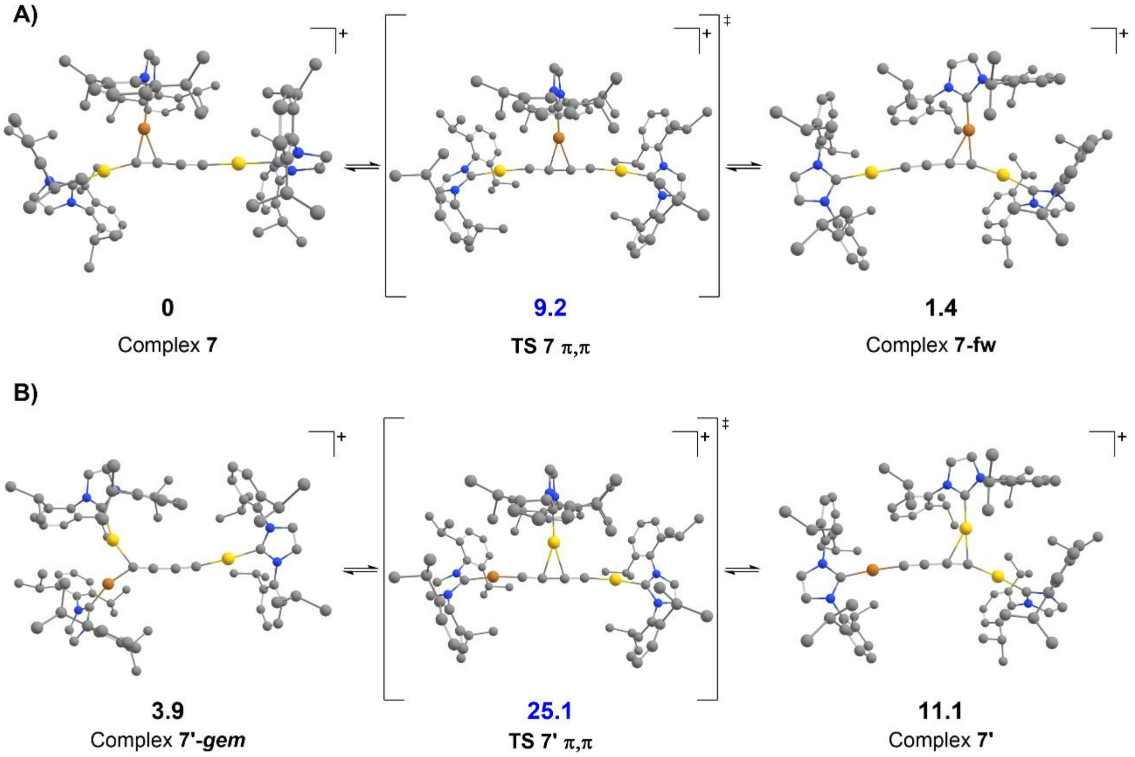

Figure S41. Calculated relative Gibbs free energies for the π,π exchange process in A) complex **7** and B) an isomer of complex **7**, where the IPrCu$^+$ fragment is σ-bound to the diacetylide bridge. H atoms have been omitted for clarity. The transition state at 9.2 kcal mol^{-1} presents a very small negative imaginary frequency (-2.4 cm^{-1}) apart from the expected one, at -70.4 cm^{-1}.

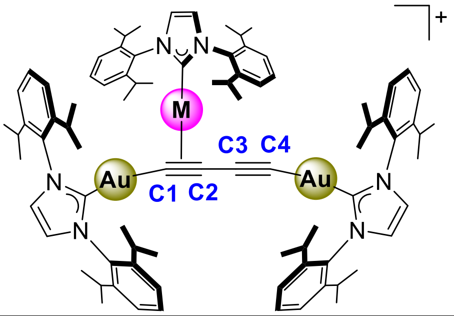

	Complex 6 (M = Au)		Complex 7 (M = Cu)	
	X-ray	DFT	X-ray	DFT
**Au1−C1−C2 (°)**	160(1)	164.2	160.7(4)	164.5
**Au1−C1 (Å)**	2.00(1)	1.99	1.985(6)	1.99
**C1−C2 (Å)**	1.23(2)	1.26	1.236(9)	1.25
**C2−C3 (Å)**	1.38(2)	1.37	1.371(9)	1.37
**C3−C4 (Å)**	1.20(2)	1.23	1.194(9)	1.23
**Au2−C4 (Å)**	1.98(1)	1.97	1.986(6)	1.97
**M3−C1 (Å)**	2.18(1)	2.18	1.956(4)	2.03
**M3−C2 (Å)**	2.25(1)	2.30	2.204(5)	2.14

Table S6. Comparison of calculated (DFT) and experimental (X-ray) geometrical features of complexes **6** and **7**. In the case of **7**, the component of the unit cell displayed in Figure 2 (main-text) is considered.

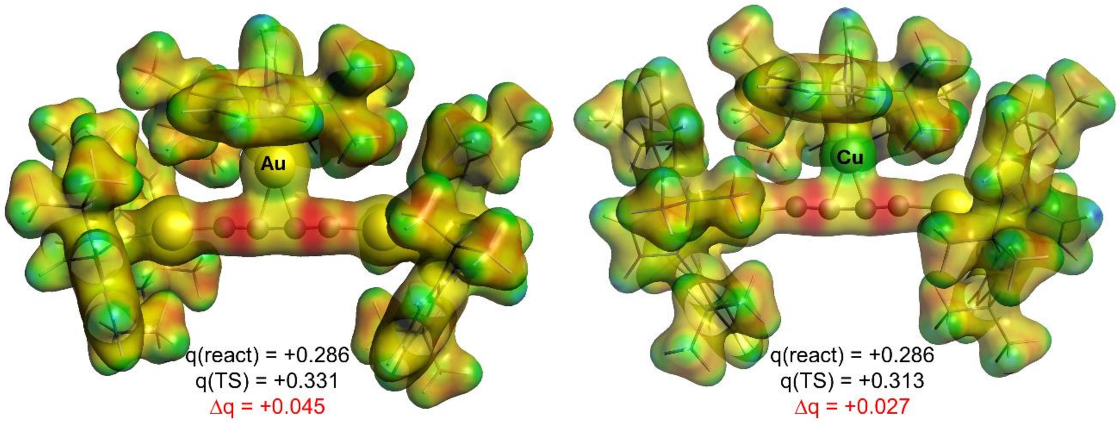

Figure S42. Molecular electrostatic potential (MESP) distribution for the transition states **TS-6** and **TS-7**, and results from charge analysis calculations. A higher charge difference between **6** and **TS-6** can be observed, in agreement with the stronger electrostatic contribution observed in the EDA calculations (see main text).

9. Cartesian coordinates of the optimized structures

Complex 1 (E(BS1) = -3959.91860875 ; G(BS1) = -3958.330094)

Au	2.511127000	0.985479000	-0.033663000
Au	-0.019601000	-1.724278000	0.375481000
Au	-2.561667000	0.843576000	-0.183439000
N	4.516763000	3.172249000	0.204890000

N	0.399432000	-4.641354000	-0.238018000
N	-0.189184000	-4.400966000	1.807812000
N	5.474374000	1.531733000	-0.779004000
C	0.051368000	-3.705096000	0.673179000
N	-5.298797000	1.444192000	-1.437326000
N	-5.007884000	2.492117000	0.406506000
C	-4.428703000	1.579915000	-0.409647000
C	0.615788000	0.389687000	0.038754000
C	-0.642092000	0.363326000	0.000140000
C	5.750183000	0.259342000	-1.377124000
C	6.596899000	-0.624535000	-0.684470000
C	4.312536000	1.890691000	-0.183970000
C	3.510063000	3.975204000	0.833286000
C	5.217913000	-0.020971000	-2.642517000
C	0.802363000	-4.308542000	-1.572433000
C	6.383898000	2.576190000	-0.770613000
H	7.366880000	2.477147000	-1.204383000
C	7.083707000	-0.303679000	0.716888000
H	7.199412000	0.783052000	0.790368000
C	0.012553000	-5.754945000	1.612422000
H	-0.126238000	-6.473914000	2.405002000
C	5.777851000	3.613687000	-0.144170000
H	6.118713000	4.611817000	0.082884000
C	2.667044000	4.740354000	0.014611000
C	0.382114000	-5.907477000	0.315260000
H	0.636113000	-6.786272000	-0.257034000
C	-0.601044000	-3.782264000	3.033298000
C	-5.798710000	-0.614553000	-2.634951000
C	-5.594992000	-1.421498000	-3.754913000
H	-6.122472000	-2.366452000	-3.837375000
C	-5.086752000	0.594529000	-2.571629000
C	5.578947000	-1.236791000	-3.233049000
H	5.193084000	-1.481962000	-4.218379000
C	-0.185256000	-4.112700000	-2.546365000
C	1.838024000	-3.123131000	3.460500000
H	1.960582000	-3.613874000	2.489962000
C	6.927481000	-1.821931000	-1.318957000
H	7.580485000	-2.530223000	-0.819531000
C	3.398790000	3.938536000	2.230483000
C	-1.964240000	-3.824373000	3.369594000
C	-6.696007000	-1.059284000	-1.496664000
H	-7.029421000	-0.165556000	-0.959410000
C	2.171932000	-4.129610000	-1.817673000
C	2.546903000	-3.742609000	-3.105251000
H	3.596293000	-3.571387000	-3.324789000
C	-3.481601000	-3.504849000	1.409079000
H	-4.126516000	-4.014328000	0.685712000
H	-2.658411000	-3.030084000	0.865098000
H	-4.064203000	-2.710597000	1.885845000
C	4.307624000	0.938493000	-3.382365000

H	4.095370000	1.785783000	-2.723772000
C	-6.402784000	2.261730000	-1.268620000
H	-7.206070000	2.290112000	-1.988619000
C	-3.556927000	4.166563000	1.401687000
C	-2.975116000	-4.496289000	2.460585000
H	-2.462335000	-5.306122000	1.930597000
C	-4.354415000	3.023800000	1.565952000
C	0.364360000	-3.135351000	3.816380000
C	6.431868000	-2.120512000	-2.585505000
H	6.712228000	-3.052972000	-3.067599000
C	6.030475000	-0.719774000	1.750385000
H	5.932806000	-1.810461000	1.778123000
H	5.047807000	-0.299035000	1.516532000
H	6.322284000	-0.380235000	2.750422000
C	8.437504000	-0.925259000	1.046087000
H	8.783258000	-0.563358000	2.019723000
H	9.194678000	-0.665212000	0.299268000
H	8.381050000	-2.017288000	1.107927000
C	-2.885377000	4.646545000	2.527824000
H	-2.251616000	5.523962000	2.435995000
C	4.284616000	3.053453000	3.083257000
H	5.089220000	2.668067000	2.448196000
C	0.241833000	-3.730570000	-3.821078000
H	-0.495624000	-3.564659000	-4.601427000
C	-6.219352000	2.923168000	-0.099252000
H	-6.826136000	3.654045000	0.412318000
C	-5.326330000	1.099711000	2.937167000
H	-5.837893000	0.917088000	1.986532000
C	2.772965000	4.725154000	-1.497282000
H	3.620467000	4.089742000	-1.772976000
C	2.395399000	4.715866000	2.813039000
H	2.271062000	4.704941000	3.891913000
C	-4.718477000	-1.034095000	-4.763808000
H	-4.573829000	-1.675624000	-5.628732000
C	3.201706000	-4.260763000	-0.714709000
H	2.744788000	-4.808591000	0.116032000
C	-4.195078000	1.012745000	-3.570040000
C	3.580433000	-2.871311000	-0.196342000
H	4.041489000	-2.272880000	-0.987762000
H	4.293643000	-2.955711000	0.629359000
H	2.697436000	-2.330280000	0.160366000
C	1.591505000	-3.547109000	-4.097375000
H	1.901895000	-3.242526000	-5.093246000
C	-4.497900000	2.359061000	2.790660000
C	-6.396968000	1.249731000	4.017971000
H	-5.950215000	1.402668000	5.006307000
H	-7.012008000	0.344829000	4.068357000
H	-7.056954000	2.098491000	3.810776000
C	-7.947751000	-1.797214000	-1.964477000
H	-8.509425000	-1.212743000	-2.700486000

H	-8.605343000	-1.988116000	-1.110129000
H	-7.709332000	-2.768014000	-2.411832000
C	-3.007948000	4.009819000	3.757980000
H	-2.474087000	4.396034000	4.621917000
C	-2.354098000	-3.174039000	4.540739000
H	-3.398801000	-3.178853000	4.833998000
C	-0.079203000	-2.499197000	4.980003000
H	0.641231000	-1.989273000	5.613346000
C	-1.421438000	-2.513739000	5.335524000
H	-1.746045000	-2.010480000	6.242148000
C	1.551972000	5.496783000	2.030394000
H	0.780485000	6.098191000	2.503498000
C	2.370159000	-1.696062000	3.322543000
H	1.811880000	-1.138693000	2.563201000
H	3.424380000	-1.711929000	3.026876000
H	2.296565000	-1.148907000	4.268460000
C	-4.026708000	0.166529000	-4.670465000
H	-3.348514000	0.460821000	-5.466271000
C	2.969134000	0.279854000	-3.719645000
H	3.096076000	-0.546702000	-4.427038000
H	2.293380000	1.010876000	-4.176912000
H	2.484262000	-0.112766000	-2.819756000
C	-3.384356000	4.847126000	0.058268000
H	-4.064475000	4.373331000	-0.656856000
C	1.687621000	5.509502000	0.646810000
H	1.018518000	6.116977000	0.044429000
C	4.435568000	-5.048128000	-1.151067000
H	4.166544000	-6.045580000	-1.514398000
H	5.120141000	-5.167497000	-0.304633000
H	4.985843000	-4.533773000	-1.944566000
C	-4.144896000	-5.121493000	3.216021000
H	-4.779004000	-4.362538000	3.686688000
H	-3.802584000	-5.810280000	3.995259000
H	-4.775866000	-5.683474000	2.519725000
C	1.519151000	4.110259000	-2.124014000
H	1.338263000	3.101661000	-1.736476000
H	1.631933000	4.045833000	-3.212191000
H	0.633062000	4.717712000	-1.911312000
C	2.648309000	-3.918708000	4.486026000
H	2.578547000	-3.468021000	5.482292000
H	3.705683000	-3.939030000	4.200414000
H	2.294986000	-4.952738000	4.558680000
C	-1.662658000	-4.261655000	-2.246960000
H	-1.766591000	-4.596040000	-1.209553000
C	-3.806872000	2.879776000	3.887475000
H	-3.890436000	2.385367000	4.851177000
C	-4.422196000	-0.103694000	3.215571000
H	-3.669847000	-0.224417000	2.428363000
H	-5.017744000	-1.021673000	3.268512000
H	-3.894245000	0.011937000	4.168738000

C	-1.960827000	4.651275000	-0.466817000
H	-1.227550000	5.105235000	0.207709000
H	-1.852623000	5.117791000	-1.452391000
H	-1.714371000	3.587658000	-0.558281000
C	3.044038000	6.122856000	-2.054527000
H	2.214204000	6.805229000	-1.840388000
H	3.167316000	6.080080000	-3.142013000
H	3.954766000	6.554425000	-1.626072000
C	-3.752232000	6.329642000	0.124875000
H	-4.775242000	6.471517000	0.488804000
H	-3.680108000	6.781395000	-0.870324000
H	-3.078612000	6.882480000	0.788615000
C	4.991457000	1.487041000	-4.636015000
H	5.930263000	1.993734000	-4.388651000
H	4.337991000	2.208491000	-5.138494000
H	5.218465000	0.686790000	-5.349236000
C	-3.456836000	2.336097000	-3.513743000
H	-3.638797000	2.789324000	-2.534650000
C	3.487325000	1.852212000	3.598778000
H	4.138461000	1.170194000	4.156456000
H	3.032026000	1.298795000	2.769989000
H	2.681942000	2.176598000	4.267607000
C	-5.890737000	-1.911603000	-0.512362000
H	-5.570760000	-2.844935000	-0.988314000
H	-6.496830000	-2.167317000	0.363958000
H	-4.994168000	-1.385275000	-0.170285000
C	-2.379297000	-2.915949000	-2.374317000
H	-1.939450000	-2.164297000	-1.709376000
H	-3.437801000	-3.020833000	-2.116728000
H	-2.321387000	-2.533560000	-3.398970000
C	-1.945323000	2.147728000	-3.652088000
H	-1.434313000	3.110664000	-3.545437000
H	-1.678290000	1.737157000	-4.632005000
H	-1.559280000	1.472474000	-2.881309000
C	4.936908000	3.821056000	4.232692000
H	4.193641000	4.188776000	4.948145000
H	5.507844000	4.680628000	3.866541000
H	5.622751000	3.165370000	4.779620000
C	-2.313309000	-5.317744000	-3.141242000
H	-3.367910000	-5.442138000	-2.872586000
H	-2.271952000	-5.027912000	-4.196887000
H	-1.818876000	-6.289417000	-3.038260000
C	-3.994813000	3.297492000	-4.576021000
H	-3.487366000	4.265674000	-4.503306000
H	-3.828485000	2.905200000	-5.585622000
H	-5.069826000	3.466868000	-4.453624000

TS 1σ.π (E = -3959.91491718 ; G = -3958.323751)

Au	2.871758000	0.497014000	-0.161459000

Au	-0.951868000	-1.635524000	0.159323000
Au	-2.109251000	1.252949000	-0.275836000
N	5.407494000	2.023214000	0.371614000
N	-1.189979000	-4.526637000	-0.509548000
N	-1.688167000	-4.264423000	1.558054000
N	5.911525000	0.128063000	-0.484888000
C	-1.356396000	-3.577830000	0.441168000
N	-4.626283000	2.861481000	-1.081677000
N	-3.687979000	3.554324000	0.715139000
C	-3.629425000	2.571997000	-0.215284000
C	0.913387000	0.308754000	-0.240616000
C	-0.339046000	0.313425000	-0.253534000
C	5.820519000	-1.184637000	-1.052646000
C	6.118325000	-2.284145000	-0.230041000
C	4.857194000	0.869936000	-0.075039000
C	4.648365000	3.135030000	0.864527000
C	5.476294000	-1.309768000	-2.405163000
C	-0.765387000	-4.206280000	-1.840254000
C	7.102638000	0.809387000	-0.305159000
H	8.052884000	0.376550000	-0.576939000
C	6.401797000	-2.102971000	1.248580000
H	6.831196000	-1.104935000	1.387696000
C	-1.721836000	-5.627023000	1.310849000
H	-1.964885000	-6.339880000	2.083413000
C	6.783186000	2.008487000	0.240469000
H	7.395078000	2.844321000	0.542232000
C	4.281829000	4.140355000	-0.043070000
C	-1.411436000	-5.792025000	0.000918000
H	-1.325330000	-6.678718000	-0.607644000
C	-1.967042000	-3.650603000	2.822177000
C	-6.001431000	1.212130000	-2.228114000
C	-6.250631000	0.461357000	-3.377541000
H	-7.083479000	-0.234458000	-3.394232000
C	-4.907867000	2.092920000	-2.257636000
C	5.448888000	-2.600891000	-2.942206000
H	5.190979000	-2.734815000	-3.989316000
C	-1.728077000	-3.788766000	-2.769696000
C	0.551426000	-3.345809000	3.184772000
H	0.572401000	-3.731493000	2.161163000
C	6.080646000	-3.549941000	-0.815549000
H	6.306474000	-4.425378000	-0.215039000
C	4.301695000	3.164261000	2.222858000
C	-3.310965000	-3.535148000	3.215814000
C	-6.833592000	1.037393000	-0.972873000
H	-6.799706000	1.977471000	-0.412032000
C	0.607820000	-4.250720000	-2.124016000
C	1.011277000	-3.859089000	-3.402293000
H	2.068183000	-3.864767000	-3.652575000
C	-4.833742000	-2.841979000	1.363638000
H	-5.597255000	-3.180816000	0.655012000

H	-3.976624000	-2.468287000	0.794922000
H	-5.246906000	-2.004200000	1.934784000
C	5.145414000	-0.118015000	-3.280718000
H	5.244116000	0.788883000	-2.676194000
C	-5.297451000	4.011198000	-0.700725000
H	-6.126605000	4.402259000	-1.269846000
C	-1.552795000	4.402670000	1.514490000
C	-4.437946000	-3.982955000	2.305372000
H	-4.065888000	-4.806611000	1.686925000
C	-2.716188000	3.659228000	1.764670000
C	-0.896987000	-3.194917000	3.604730000
C	5.756663000	-3.706189000	-2.160095000
H	5.739579000	-4.700353000	-2.597612000
C	5.088764000	-2.153455000	2.037899000
H	4.645094000	-3.153824000	1.977566000
H	4.358505000	-1.437952000	1.646079000
H	5.266360000	-1.924184000	3.094548000
C	7.406175000	-3.110006000	1.801679000
H	7.653652000	-2.854363000	2.837035000
H	8.334911000	-3.115094000	1.221952000
H	7.002441000	-4.128014000	1.807485000
C	-0.573543000	4.414767000	2.509603000
H	0.347247000	4.965000000	2.341590000
C	4.645063000	2.032451000	3.169639000
H	5.308182000	1.339125000	2.642448000
C	-1.273508000	-3.409810000	-4.035458000
H	-1.991541000	-3.075300000	-4.779207000
C	-4.704328000	4.447439000	0.439075000
H	-4.907148000	5.299243000	1.069438000
C	-4.163394000	2.096853000	3.175689000
H	-4.836791000	2.245884000	2.325317000
C	4.589335000	4.037351000	-1.523505000
H	5.292004000	3.210444000	-1.668535000
C	3.578051000	4.270333000	2.675205000
H	3.288754000	4.326006000	3.720835000
C	-5.440831000	0.587809000	-4.502394000
H	-5.651624000	-0.005962000	-5.387636000
C	1.633298000	-4.634683000	-1.075893000
H	1.101261000	-5.066388000	-0.221904000
C	-4.076772000	2.247732000	-3.376041000
C	2.367683000	-3.388389000	-0.575633000
H	2.932147000	-2.912373000	-1.383987000
H	3.073154000	-3.654066000	0.217882000
H	1.662155000	-2.650235000	-0.180294000
C	0.080588000	-3.442043000	-4.347988000
H	0.414656000	-3.134725000	-5.335257000
C	-2.925424000	2.939619000	2.949077000
C	-4.912855000	2.524644000	4.437937000
H	-4.305976000	2.367809000	5.336197000
H	-5.829902000	1.936408000	4.550332000

H	-5.190344000	3.583202000	4.398483000
C	-8.301715000	0.733443000	-1.258234000
H	-8.746705000	1.477796000	-1.926559000
H	-8.868428000	0.736491000	-0.321536000
H	-8.433053000	-0.254191000	-1.712773000
C	-0.751760000	3.712839000	3.696544000
H	0.026906000	3.727592000	4.454451000
C	-3.568157000	-2.936197000	4.449352000
H	-4.593065000	-2.825908000	4.788824000
C	-1.209956000	-2.599134000	4.830651000
H	-0.406475000	-2.236811000	5.465685000
C	-2.527766000	-2.472099000	5.248871000
H	-2.749032000	-2.007718000	6.205997000
C	3.223036000	5.296092000	1.806668000
H	2.670103000	6.153544000	2.180826000
C	1.278045000	-1.999999000	3.180234000
H	0.761497000	-1.273070000	2.545485000
H	2.294490000	-2.122871000	2.793773000
H	1.353831000	-1.581492000	4.189844000
C	-4.368912000	1.470488000	-4.501320000
H	-3.749142000	1.567137000	-5.388236000
C	3.696827000	-0.186841000	-3.768685000
H	3.527294000	-1.074373000	-4.388495000
H	3.456804000	0.695270000	-4.372293000
H	2.998636000	-0.222499000	-2.924728000
C	-1.325450000	5.145916000	0.212837000
H	-2.224214000	5.039673000	-0.403363000
C	3.564513000	5.227864000	0.460067000
H	3.264026000	6.025880000	-0.212540000
C	2.609941000	-5.693700000	-1.585109000
H	2.084325000	-6.595736000	-1.915919000
H	3.304646000	-5.977119000	-0.787295000
H	3.207607000	-5.322867000	-2.423519000
C	-5.656538000	-4.502646000	3.063456000
H	-6.167719000	-3.704244000	3.611820000
H	-5.384942000	-5.286655000	3.777795000
H	-6.379154000	-4.923266000	2.356744000
C	3.314768000	3.698895000	-2.302446000
H	2.845424000	2.786877000	-1.915944000
H	3.544789000	3.546782000	-3.362906000
H	2.583370000	4.511309000	-2.227999000
C	1.271392000	-4.358721000	4.077544000
H	1.292691000	-4.020995000	5.119744000
H	2.307177000	-4.488834000	3.744982000
H	0.779789000	-5.336994000	4.050264000
C	-3.203361000	-3.712155000	-2.434766000
H	-3.340077000	-4.074818000	-1.410893000
C	-1.917169000	2.986822000	3.915112000
H	-2.043802000	2.434436000	4.841989000
C	-3.800172000	0.611081000	3.226648000

H	-3.285463000	0.295322000	2.312501000
H	-4.702883000	0.002100000	3.343270000
H	-3.138396000	0.396285000	4.072563000
C	-0.156250000	4.543379000	-0.569829000
H	0.781259000	4.626023000	-0.009920000
H	-0.029473000	5.069504000	-1.522762000
H	-0.323381000	3.482142000	-0.780572000
C	5.252728000	5.301534000	-2.067823000
H	4.584758000	6.167145000	-2.005975000
H	5.515239000	5.163536000	-3.122018000
H	6.168706000	5.541271000	-1.517847000
C	-1.110395000	6.640315000	0.458445000
H	-1.952855000	7.082615000	1.000551000
H	-1.003425000	7.169382000	-0.494608000
H	-0.201444000	6.821109000	1.042745000
C	6.123549000	-0.000816000	-4.450234000
H	7.157808000	0.073500000	-4.098167000
H	5.899959000	0.893898000	-5.041132000
H	6.055928000	-0.866754000	-5.117933000
C	-2.924757000	3.232386000	-3.415247000
H	-2.824753000	3.683455000	-2.423329000
C	3.378990000	1.259957000	3.551036000
H	3.630026000	0.406022000	4.189708000
H	2.863290000	0.886387000	2.659271000
H	2.678904000	1.900277000	4.099757000
C	-6.216107000	-0.049648000	-0.088432000
H	-6.262380000	-1.020825000	-0.592283000
H	-6.762789000	-0.130489000	0.857480000
H	-5.165987000	0.160560000	0.136813000
C	-3.700202000	-2.265448000	-2.484649000
H	-3.128489000	-1.622238000	-1.805621000
H	-4.755477000	-2.215694000	-2.196979000
H	-3.609265000	-1.850907000	-3.494437000
C	-1.600412000	2.538362000	-3.736464000
H	-0.778177000	3.261387000	-3.706004000
H	-1.613755000	2.090679000	-4.736249000
H	-1.383213000	1.747899000	-3.009770000
C	5.388955000	2.526417000	4.409974000
H	4.764137000	3.189344000	5.017993000
H	6.298481000	3.073051000	4.139775000
H	5.677296000	1.677092000	5.038563000
C	-4.030483000	-4.609596000	-3.356498000
H	-5.085808000	-4.581895000	-3.064302000
H	-3.965203000	-4.279085000	-4.398829000
H	-3.691791000	-5.649946000	-3.310390000
C	-3.214746000	4.359972000	-4.408291000
H	-2.399750000	5.091851000	-4.398927000
H	-3.308096000	3.973943000	-5.429448000
H	-4.144504000	4.882319000	-4.159251000

Complex 1-*gem* (E = -3959.91511930 ; G = -3958.327777)

Au	2.974530000	0.283347000	-0.197597000
Au	-1.331679000	-1.527788000	0.111024000
Au	-1.868629000	1.383283000	-0.287064000
N	5.666032000	1.512447000	0.385796000
N	-1.856792000	-4.367314000	-0.574524000
N	-2.360168000	-4.074885000	1.488566000
N	5.946970000	-0.436631000	-0.450941000
C	-1.966373000	-3.411302000	0.378062000
N	-4.095375000	3.363740000	-1.095996000
N	-3.121477000	3.862205000	0.746291000
C	-3.169821000	2.915109000	-0.219294000
C	1.012442000	0.208297000	-0.280479000
C	-0.237045000	0.180737000	-0.262272000
C	5.695636000	-1.747242000	-0.973582000
C	5.756905000	-2.834582000	-0.086041000
C	4.985726000	0.440118000	-0.080174000
C	5.038049000	2.692845000	0.901214000
C	5.417634000	-1.888387000	-2.339702000
C	-1.362276000	-4.075023000	-1.888099000
C	7.210114000	0.081762000	-0.226793000
H	8.104218000	-0.474342000	-0.462517000
C	5.968432000	-2.630381000	1.401460000
H	6.470568000	-1.667569000	1.543519000
C	-2.493379000	-5.430134000	1.234129000
H	-2.800885000	-6.125437000	1.999721000
C	7.031759000	1.315597000	0.306448000
H	7.737214000	2.063722000	0.632849000
C	4.838806000	3.775366000	0.032081000
C	-2.178819000	-5.613763000	-0.072468000
H	-2.151700000	-6.502810000	-0.683187000
C	-2.595279000	-3.454964000	2.759105000
C	-5.649431000	1.928347000	-2.296477000
C	-5.976028000	1.243110000	-3.467052000
H	-6.888563000	0.657100000	-3.508417000
C	-4.455201000	2.668080000	-2.295039000
C	5.206858000	-3.184567000	-2.820199000
H	4.987471000	-3.330697000	-3.874190000
C	-2.255665000	-3.577181000	-2.846506000
C	-0.060517000	-3.332703000	3.127710000
H	-0.063030000	-3.688360000	2.092965000
C	5.547994000	-4.107880000	-0.617569000
H	5.588066000	-4.973773000	0.035874000
C	4.647044000	2.709724000	2.247877000
C	-3.928243000	-3.247159000	3.152983000
C	-6.510561000	1.829796000	-1.052050000
H	-6.378688000	2.753470000	-0.478178000
C	0.013593000	-4.220841000	-2.124019000
C	0.492016000	-3.844128000	-3.380802000

H	1.554884000	-3.924359000	-3.591795000
C	-5.355914000	-2.393128000	1.296257000
H	-6.128159000	-2.652759000	0.563908000
H	-4.456151000	-2.083947000	0.755318000
H	-5.710044000	-1.532874000	1.873857000
C	5.325201000	-0.704139000	-3.279787000
H	5.581856000	0.198217000	-2.716020000
C	-4.618894000	4.578139000	-0.686327000
H	-5.373507000	5.095465000	-1.258319000
C	-0.934479000	4.386224000	1.677930000
C	-5.080178000	-3.580180000	2.224775000
H	-4.776006000	-4.424682000	1.597251000
C	-2.191834000	3.783589000	1.835179000
C	-1.496288000	-3.089952000	3.549519000
C	5.281754000	-4.282056000	-1.972116000
H	5.125174000	-5.282141000	-2.366444000
C	4.611822000	-2.550533000	2.109926000
H	4.082347000	-3.506841000	2.030616000
H	3.976424000	-1.775791000	1.667281000
H	4.746581000	-2.323972000	3.173434000
C	6.852577000	-3.701424000	2.035412000
H	7.059252000	-3.441903000	3.078822000
H	7.810116000	-3.795122000	1.512898000
H	6.367843000	-4.683408000	2.035807000
C	-0.013531000	4.228406000	2.715799000
H	0.976631000	4.663420000	2.617352000
C	4.807477000	1.509287000	3.158561000
H	5.335177000	0.727059000	2.603910000
C	-1.727887000	-3.218991000	-4.089579000
H	-2.391288000	-2.823584000	-4.853658000
C	-4.004383000	4.891346000	0.482670000
H	-4.109764000	5.739562000	1.141076000
C	-3.886180000	2.340101000	3.094733000
H	-4.493399000	2.605962000	2.223457000
C	5.179768000	3.697670000	-1.442330000
H	5.737470000	2.771846000	-1.615280000
C	4.061973000	3.882877000	2.730731000
H	3.746859000	3.929718000	3.769513000
C	-5.143618000	1.296126000	-4.581573000
H	-5.414979000	0.753856000	-5.483220000
C	0.971863000	-4.711123000	-1.056844000
H	0.381978000	-5.058271000	-0.202403000
C	-3.599270000	2.747828000	-3.401672000
C	1.859300000	-3.565824000	-0.564749000
H	2.486539000	-3.172994000	-1.371702000
H	2.519686000	-3.912972000	0.236056000
H	1.254299000	-2.739041000	-0.180759000
C	-0.369080000	-3.347322000	-4.353312000
H	0.023315000	-3.053268000	-5.322956000
C	-2.546354000	3.035547000	2.966731000

C	-4.641308000	2.800421000	4.342174000
H	-4.103725000	2.529525000	5.257507000
H	-5.627474000	2.325556000	4.384012000
H	-4.786246000	3.885803000	4.344104000
C	-7.999957000	1.695292000	-1.356288000
H	-8.352155000	2.493165000	-2.018161000
H	-8.572711000	1.749222000	-0.424818000
H	-8.235524000	0.734202000	-1.825569000
C	-0.337877000	3.500486000	3.854861000
H	0.394647000	3.384631000	4.649129000
C	-4.144257000	-2.660299000	4.399902000
H	-5.159254000	-2.482275000	4.740401000
C	-1.768595000	-2.501874000	4.789007000
H	-0.941729000	-2.211565000	5.430849000
C	-3.074580000	-2.293133000	5.211365000
H	-3.263635000	-1.837408000	6.179485000
C	3.875752000	4.982899000	1.901360000
H	3.425851000	5.889114000	2.298060000
C	0.762416000	-2.043552000	3.161971000
H	0.304710000	-1.264647000	2.543867000
H	1.769105000	-2.229903000	2.774578000
H	0.863996000	-1.657631000	4.182388000
C	-3.971091000	2.039171000	-4.548662000
H	-3.332926000	2.079557000	-5.426932000
C	3.893439000	-0.536786000	-3.793876000
H	3.580686000	-1.407920000	-4.380713000
H	3.819913000	0.348079000	-4.435825000
H	3.188745000	-0.415876000	-2.963232000
C	-0.542208000	5.139358000	0.422310000
H	-1.421444000	5.205034000	-0.226845000
C	4.251702000	4.925717000	0.563565000
H	4.077200000	5.781671000	-0.081977000
C	1.806295000	-5.894802000	-1.545713000
H	1.170435000	-6.725401000	-1.870203000
H	2.454943000	-6.256427000	-0.740568000
H	2.449522000	-5.613476000	-2.385800000
C	-6.351200000	-3.997854000	2.958414000
H	-6.792431000	-3.165827000	3.517300000
H	-6.163770000	-4.817832000	3.659267000
H	-7.100485000	-4.334500000	2.234726000
C	3.895495000	3.623260000	-2.273455000
H	3.272040000	2.776445000	-1.965374000
H	4.134801000	3.504246000	-3.336187000
H	3.303948000	4.538335000	-2.159281000
C	0.580812000	-4.420784000	3.991514000
H	0.630316000	-4.112943000	5.042011000
H	1.602825000	-4.621774000	3.651817000
H	0.015328000	-5.357337000	3.941427000
C	-3.733111000	-3.394731000	-2.564442000
H	-3.934539000	-3.758899000	-1.551900000

C	-1.590899000	2.911119000	3.978286000
H	-1.829758000	2.333481000	4.866920000
C	-3.706289000	0.820486000	3.089407000
H	-3.183393000	0.483148000	2.187321000
H	-4.679787000	0.321156000	3.130778000
H	-3.123099000	0.489674000	3.955125000
C	0.544104000	4.383177000	-0.346734000
H	1.458653000	4.291702000	0.248474000
H	0.792929000	4.916568000	-1.271020000
H	0.215648000	3.372198000	-0.608608000
C	6.065605000	4.858757000	-1.892194000
H	5.550574000	5.820535000	-1.795683000
H	6.340267000	4.736253000	-2.945194000
H	6.987773000	4.908502000	-1.303947000
C	-0.098642000	6.567923000	0.740489000
H	-0.877221000	7.118635000	1.278789000
H	0.123312000	7.109600000	-0.185241000
H	0.808305000	6.577978000	1.355061000
C	6.319736000	-0.825487000	-4.435156000
H	7.345987000	-0.932293000	-4.068469000
H	6.275762000	0.067996000	-5.067128000
H	6.095171000	-1.690892000	-5.067960000
C	-2.330256000	3.577328000	-3.401996000
H	-2.195694000	4.001681000	-2.402207000
C	3.436862000	0.950918000	3.550696000
H	3.552635000	0.050384000	4.163454000
H	2.848973000	0.692553000	2.662861000
H	2.865470000	1.683322000	4.131915000
C	-6.030688000	0.670356000	-0.173917000
H	-6.178850000	-0.284680000	-0.688566000
H	-6.597022000	0.643715000	0.763297000
H	-4.966864000	0.758945000	0.067232000
C	-4.119807000	-1.914915000	-2.611688000
H	-3.537536000	-1.325943000	-1.894173000
H	-5.182033000	-1.792259000	-2.375416000
H	-3.948202000	-1.493396000	-3.607875000
C	-1.101178000	2.718309000	-3.703302000
H	-0.190560000	3.323640000	-3.637112000
H	-1.147824000	2.293605000	-4.712216000
H	-1.012899000	1.893290000	-2.988333000
C	5.645300000	1.846718000	4.391973000
H	5.158823000	2.610049000	5.008838000
H	6.635579000	2.220514000	4.111442000
H	5.781515000	0.954454000	5.012326000
C	-4.588381000	-4.217580000	-3.529357000
H	-5.648998000	-4.117532000	-3.274604000
H	-4.461563000	-3.878799000	-4.563374000
H	-4.327227000	-5.280210000	-3.488034000
C	-2.445988000	4.743657000	-4.385762000
H	-1.542589000	5.362091000	-4.348777000

H	-2.564670000	4.384661000	-5.414084000
H	-3.304824000	5.380787000	-4.149683000

Complex 3 (E = -4021.46511533 ; G = -4019.873159)

Au	-2.540688000	-0.798845000	0.025238000
Au	2.525978000	-0.683883000	-0.134518000
N	-4.570509000	-2.983156000	0.087417000
N	-0.240043000	4.580666000	-0.071571000
N	0.358493000	4.263542000	1.953481000
N	-5.508219000	-1.258065000	-0.760277000
C	0.052834000	3.597060000	0.812146000
N	5.187290000	-1.293153000	-1.556782000
N	4.948941000	-2.418769000	0.248320000
C	4.364670000	-1.445585000	-0.491187000
C	-0.641214000	-0.225791000	0.166960000
C	0.612876000	-0.215235000	0.137582000
C	-5.768599000	0.058160000	-1.261982000
C	-6.570722000	0.912437000	-0.484441000
C	-4.347141000	-1.679206000	-0.205601000
C	-3.576683000	-3.853803000	0.641187000
C	-5.273574000	0.408482000	-2.525029000
C	-0.697647000	4.325701000	-1.404674000
C	-6.436458000	-2.284111000	-0.819858000
H	-7.421488000	-2.136154000	-1.234709000
C	-7.016752000	0.510036000	0.908915000
H	-7.159645000	-0.576223000	0.913064000
C	0.248176000	5.632782000	1.788923000
H	0.443882000	6.325973000	2.592551000
C	-5.842456000	-3.375586000	-0.280032000
H	-6.197989000	-4.382356000	-0.125052000
C	-2.770939000	-4.583596000	-0.244355000
C	-0.127764000	5.834744000	0.501631000
H	-0.331061000	6.739159000	-0.050863000
C	0.757336000	3.604172000	3.160918000
C	5.741032000	0.796252000	-2.674424000
C	5.533497000	1.669040000	-3.743338000
H	6.106887000	2.588591000	-3.804118000
C	4.968605000	-0.376909000	-2.637202000
C	-5.622113000	1.669044000	-3.021344000
H	-5.261473000	1.970323000	-4.000771000
C	0.244330000	4.105251000	-2.419075000
C	-1.694061000	2.995312000	3.588602000
H	-1.809164000	3.511262000	2.630768000
C	-6.894718000	2.154808000	-1.028516000
H	-7.515435000	2.842275000	-0.463504000
C	-3.444901000	-3.923024000	2.035081000
C	2.120959000	3.616386000	3.503756000
C	6.710953000	1.136356000	-1.559921000
H	7.039863000	0.199436000	-1.098697000
C	-2.083346000	4.281707000	-1.621054000

C	-2.523401000	4.012380000	-2.918095000
H	-3.588553000	3.958506000	-3.120421000
C	3.540987000	3.390907000	1.456408000
H	4.203815000	3.922673000	0.766375000
H	2.671028000	3.038053000	0.894731000
H	4.072305000	2.511281000	1.830982000
C	-4.409431000	-0.519733000	-3.354560000
H	-4.260710000	-1.444639000	-2.789601000
C	6.264048000	-2.160180000	-1.485197000
H	7.027371000	-2.184140000	-2.247634000
C	3.500514000	-4.075604000	1.280991000
C	3.142190000	4.304526000	2.618289000
H	2.666851000	5.193431000	2.189318000
C	4.354627000	-2.972278000	1.429355000
C	-0.218481000	2.960863000	3.933696000
C	-6.433339000	2.525327000	-2.289105000
H	-6.708365000	3.492899000	-2.699693000
C	-5.916095000	0.829483000	1.927314000
H	-5.780172000	1.912910000	2.015317000
H	-4.956042000	0.392995000	1.634947000
H	-6.185800000	0.439981000	2.915294000
C	-8.340190000	1.141309000	1.331001000
H	-8.661590000	0.718665000	2.288306000
H	-9.130477000	0.955851000	0.596266000
H	-8.250725000	2.224220000	1.469148000
C	2.897242000	-4.578802000	2.435220000
H	2.221599000	-5.425679000	2.356824000
C	-4.294196000	-3.082860000	2.966471000
H	-5.071399000	-2.595752000	2.368655000
C	-0.246214000	3.836033000	-3.699920000
H	0.453095000	3.652610000	-4.510447000
C	6.114230000	-2.870199000	-0.340303000
H	6.716469000	-3.646639000	0.105201000
C	5.514152000	-1.155751000	2.801758000
H	5.922646000	-0.922790000	1.813218000
C	-2.895055000	-4.449103000	-1.748906000
H	-3.744153000	-3.792457000	-1.963667000
C	-2.459328000	-4.772241000	2.543294000
H	-2.320466000	-4.843527000	3.618232000
C	4.598588000	1.377540000	-4.731783000
H	4.451704000	2.069584000	-5.556509000
C	-3.063420000	4.437488000	-0.476366000
H	-2.549033000	4.950951000	0.342554000
C	4.022609000	-0.701824000	-3.620422000
C	-3.479044000	3.054763000	0.034121000
H	-3.995251000	2.490695000	-0.748730000
H	-4.156094000	3.151846000	0.888783000
H	-2.607736000	2.469599000	0.348142000
C	-1.613266000	3.793874000	-3.947377000
H	-1.973490000	3.583721000	-4.950849000

C	4.618905000	-2.369666000	2.666267000
C	6.692468000	-1.428031000	3.737073000
H	6.352651000	-1.645392000	4.755529000
H	7.348603000	-0.552252000	3.784989000
H	7.287524000	-2.279891000	3.391672000
C	7.961367000	1.864993000	-2.044480000
H	8.460467000	1.319514000	-2.851970000
H	8.670982000	1.970373000	-1.217433000
H	7.733622000	2.873672000	-2.405080000
C	3.140368000	-4.004015000	3.677695000
H	2.658844000	-4.408477000	4.563728000
C	2.497935000	2.932618000	4.659584000
H	3.541769000	2.915398000	4.955296000
C	0.212480000	2.287165000	5.081545000
H	-0.518554000	1.778144000	5.703541000
C	1.553864000	2.267474000	5.437401000
H	1.869113000	1.736588000	6.331489000
C	-1.653179000	-5.520291000	1.692551000
H	-0.895074000	-6.178873000	2.107831000
C	-2.262481000	1.585968000	3.422201000
H	-1.723802000	1.030515000	2.647677000
H	-3.317650000	1.633191000	3.134208000
H	-2.195555000	1.015248000	4.354619000
C	3.853683000	0.207304000	-4.669816000
H	3.132983000	-0.014004000	-5.451940000
C	-3.029304000	0.092199000	-3.601235000
H	-3.100494000	1.009435000	-4.195438000
H	-2.393927000	-0.613799000	-4.147397000
H	-2.530081000	0.336332000	-2.657230000
C	3.195987000	-4.688630000	-0.070921000
H	3.849550000	-4.220990000	-0.814240000
C	-1.808652000	-5.427555000	0.313920000
H	-1.166722000	-6.008840000	-0.341576000
C	-4.280877000	5.281575000	-0.845045000
H	-3.986804000	6.270508000	-1.212109000
H	-4.917188000	5.420600000	0.035292000
H	-4.891227000	4.799542000	-1.613969000
C	4.381265000	4.776077000	3.373217000
H	4.986430000	3.935229000	3.729274000
H	4.120563000	5.397683000	4.236083000
H	5.014544000	5.370345000	2.706581000
C	-1.646012000	-3.786540000	-2.336267000
H	-1.455050000	-2.815992000	-1.865193000
H	-1.771283000	-3.628605000	-3.413436000
H	-0.760691000	-4.413849000	-2.187053000
C	-2.476701000	3.787522000	4.637849000
H	-2.412654000	3.312366000	5.623079000
H	-3.534984000	3.842062000	4.359831000
H	-2.095709000	4.809983000	4.731281000
C	1.733128000	4.084377000	-2.141977000

H	1.896701000	4.523747000	-1.151896000
C	3.993505000	-2.912667000	3.791357000
H	4.171633000	-2.466535000	4.765827000
C	4.704238000	0.056941000	3.264814000
H	3.866337000	0.252277000	2.587280000
H	5.340279000	0.948387000	3.296370000
H	4.294074000	-0.100308000	4.268539000
C	1.750960000	-4.397315000	-0.479753000
H	1.044636000	-4.843811000	0.227497000
H	1.547562000	-4.813498000	-1.472713000
H	1.553598000	-3.319838000	-0.508036000
C	-3.175682000	-5.795983000	-2.415321000
H	-2.346459000	-6.496452000	-2.268227000
H	-3.310888000	-5.663532000	-3.494120000
H	-4.083173000	-6.258741000	-2.013452000
C	3.482596000	-6.190368000	-0.090707000
H	4.518236000	-6.404363000	0.193212000
H	3.315748000	-6.591537000	-1.096050000
H	2.824880000	-6.735081000	0.595455000
C	-5.097358000	-0.886748000	-4.670302000
H	-6.073850000	-1.350124000	-4.494366000
H	-4.481074000	-1.595237000	-5.234260000
H	-5.251319000	-0.003516000	-5.299856000
C	3.228498000	-1.993024000	-3.604213000
H	3.450230000	-2.521337000	-2.671959000
C	-3.445343000	-1.980788000	3.605484000
H	-4.068560000	-1.327448000	4.226026000
H	-2.956947000	-1.369891000	2.838484000
H	-2.662520000	-2.408929000	4.241895000
C	5.984909000	1.948010000	-0.484980000
H	5.665157000	2.914927000	-0.889042000
H	6.642648000	2.137732000	0.370681000
H	5.094883000	1.423345000	-0.125632000
C	2.225929000	2.636370000	-2.098851000
H	1.668630000	2.051248000	-1.359849000
H	3.287946000	2.592951000	-1.838827000
H	2.096361000	2.149858000	-3.071460000
C	1.722239000	-1.729863000	-3.632183000
H	1.173211000	-2.675192000	-3.565417000
H	1.418067000	-1.232841000	-4.560068000
H	1.416398000	-1.100388000	-2.790098000
C	-4.994513000	-3.932204000	4.026884000
H	-4.275322000	-4.417728000	4.695355000
H	-5.611205000	-4.713134000	3.569863000
H	-5.644963000	-3.302939000	4.643673000
C	2.535826000	4.904268000	-3.150487000
H	3.593140000	4.912851000	-2.864328000
H	2.475776000	4.478493000	-4.157784000
H	2.186681000	5.941034000	-3.197849000
C	3.653406000	-2.902580000	-4.759363000

H	3.111717000	-3.853528000	-4.710836000
H	3.435315000	-2.441640000	-5.729141000
H	4.726233000	-3.119473000	-4.723383000
Cu	0.000519000	1.714516000	0.501284000

Complex 3' (E = -4021.45337517 ; G = -4019.863032)

Au	-2.432422000	0.705132000	-0.180988000
N	4.090128000	3.643856000	0.242007000
N	1.151456000	-4.464737000	-0.247485000
N	0.484546000	-4.326120000	1.782833000
N	5.248385000	2.179486000	-0.788725000
C	0.680622000	-3.586277000	0.666623000
N	-5.184653000	1.013040000	-1.507830000
N	-5.073998000	2.062335000	0.354244000
C	-4.361881000	1.244713000	-0.457729000
C	0.768852000	0.551633000	0.142564000
C	-0.482126000	0.408327000	0.066508000
C	5.685854000	0.973279000	-1.423430000
C	6.707719000	0.230774000	-0.804294000
C	4.067283000	2.342319000	-0.141773000
C	2.988817000	4.291749000	0.887674000
C	5.122091000	0.618093000	-2.656809000
C	1.550249000	-4.089070000	-1.571576000
C	5.981630000	3.354295000	-0.818791000
H	6.945648000	3.413284000	-1.299987000
C	7.230995000	0.612843000	0.568086000
H	7.173667000	1.702888000	0.656503000
C	0.833771000	-5.647419000	1.573489000
H	0.751168000	-6.390836000	2.351331000
C	5.247152000	4.282873000	-0.160436000
H	5.432998000	5.324110000	0.053285000
C	2.040878000	4.937811000	0.081623000
C	1.252647000	-5.736134000	0.286075000
H	1.613635000	-6.572138000	-0.292684000
C	-0.024412000	-3.780220000	3.005649000
C	-5.391854000	-1.087525000	-2.719006000
C	-5.060887000	-1.857164000	-3.834973000
H	-5.457587000	-2.863136000	-3.929410000
C	-4.845300000	0.204317000	-2.640697000
C	5.628324000	-0.522999000	-3.288067000
H	5.218434000	-0.820541000	-4.249042000
C	0.571040000	-3.973493000	-2.566566000
C	2.325390000	-2.910743000	3.527700000
H	2.523093000	-3.378507000	2.558073000
C	7.180625000	-0.894810000	-1.478629000
H	7.970930000	-1.491545000	-1.035482000
C	2.899745000	4.240655000	2.286192000
C	-1.384567000	-3.972422000	3.300187000
C	-6.246890000	-1.649278000	-1.599662000
H	-6.728917000	-0.809757000	-1.087833000

C	2.910493000	-3.825209000	-1.795273000
C	3.284970000	-3.442800000	-3.083902000
H	4.325812000	-3.218300000	-3.292365000
C	-2.840607000	-3.833133000	1.265698000
H	-3.408256000	-4.417346000	0.534030000
H	-2.046736000	-3.295336000	0.737844000
H	-3.511423000	-3.086421000	1.700294000
C	4.024752000	1.424479000	-3.322882000
H	3.768794000	2.262223000	-2.667463000
C	-6.390221000	1.675513000	-1.355714000
H	-7.173312000	1.611447000	-2.095386000
C	-4.003832000	3.907842000	1.523449000
C	-2.284538000	-4.749858000	2.358835000
H	-1.670200000	-5.512225000	1.867102000
C	-4.554934000	2.617095000	1.569250000
C	0.848041000	-3.059810000	3.831868000
C	6.652217000	-1.263647000	-2.713104000
H	7.042330000	-2.138043000	-3.226854000
C	6.331631000	0.020923000	1.659486000
H	6.397727000	-1.072439000	1.658907000
H	5.283452000	0.297143000	1.509743000
H	6.643237000	0.379647000	2.646762000
C	8.686837000	0.216914000	0.796214000
H	9.034678000	0.624064000	1.751061000
H	9.339521000	0.601565000	0.005804000
H	8.813294000	-0.870097000	0.843050000
C	-3.469913000	4.417218000	2.708247000
H	-3.028654000	5.409088000	2.712870000
C	3.918762000	3.490904000	3.119814000
H	4.799642000	3.315116000	2.492984000
C	0.996983000	-3.582741000	-3.839375000
H	0.265053000	-3.476437000	-4.635092000
C	-6.322021000	2.334970000	-0.173350000
H	-7.032419000	2.968101000	0.335209000
C	-5.201083000	0.454516000	2.771585000
H	-5.582572000	0.222183000	1.772399000
C	2.131320000	4.937308000	-1.431094000
H	3.070800000	4.453864000	-1.716963000
C	1.801788000	4.865560000	2.881689000
H	1.690005000	4.835582000	3.961418000
C	-4.222648000	-1.355274000	-4.825891000
H	-3.976602000	-1.970546000	-5.687054000
C	3.918356000	-3.868777000	-0.664891000
H	3.509840000	-4.502415000	0.129394000
C	-4.003923000	0.741097000	-3.625093000
C	4.101941000	-2.462438000	-0.086183000
H	4.519806000	-1.787426000	-0.839395000
H	4.786263000	-2.489285000	0.767640000
H	3.147142000	-2.041869000	0.248718000
C	2.337740000	-3.325102000	-4.096376000

H	2.648282000	-3.023904000	-5.093300000
C	-4.598089000	1.843182000	2.736146000
C	-6.380700000	0.388528000	3.742971000
H	-6.061521000	0.583719000	4.772634000
H	-6.836035000	-0.607453000	3.718435000
H	-7.152156000	1.121434000	3.484461000
C	-7.355684000	-2.575204000	-2.092158000
H	-7.977295000	-2.091517000	-2.852629000
H	-8.001697000	-2.856409000	-1.254103000
H	-6.956306000	-3.501627000	-2.518061000
C	-3.489728000	3.669870000	3.881556000
H	-3.066068000	4.084172000	4.792278000
C	-1.873179000	-3.385340000	4.467938000
H	-2.918778000	-3.502898000	4.733024000
C	0.307730000	-2.491621000	4.989714000
H	0.953310000	-1.925679000	5.655608000
C	-1.036955000	-2.645591000	5.299654000
H	-1.438695000	-2.193344000	6.202253000
C	0.850118000	5.524122000	2.110202000
H	0.005381000	6.007918000	2.592958000
C	2.733681000	-1.441540000	3.419270000
H	2.147841000	-0.926856000	2.650721000
H	3.793464000	-1.361530000	3.154262000
H	2.586756000	-0.914153000	4.368010000
C	-3.701003000	-0.072702000	-4.721059000
H	-3.056704000	0.312579000	-5.506222000
C	2.758166000	0.586590000	-3.507523000
H	2.931098000	-0.253910000	-4.188032000
H	1.955698000	1.200839000	-3.930583000
H	2.403847000	0.180840000	-2.553959000
C	-3.928899000	4.696965000	0.230975000
H	-4.677254000	4.291295000	-0.457960000
C	0.971565000	5.563838000	0.726025000
H	0.220346000	6.077277000	0.132760000
C	5.259133000	-4.470557000	-1.078363000
H	5.135497000	-5.466323000	-1.516689000
H	5.909074000	-4.562717000	-0.201807000
H	5.781816000	-3.839369000	-1.803307000
C	-3.419076000	-5.480550000	3.071756000
H	-4.159074000	-4.785309000	3.482419000
H	-3.048343000	-6.106565000	3.889918000
H	-3.944096000	-6.127013000	2.361027000
C	0.991549000	4.116063000	-2.038722000
H	0.978405000	3.096512000	-1.639365000
H	1.103036000	4.059471000	-3.127333000
H	0.019818000	4.571547000	-1.820910000
C	3.168330000	-3.642611000	4.574353000
H	3.029721000	-3.206508000	5.569845000
H	4.232022000	-3.571531000	4.322224000
H	2.902284000	-4.703361000	4.631300000

C	-0.899215000	-4.218715000	-2.296345000
H	-1.001380000	-4.573139000	-1.265602000
C	-4.050503000	2.399129000	3.895488000
H	-4.063951000	1.824238000	4.817194000
C	-4.141243000	-0.592959000	3.115875000
H	-3.316313000	-0.573160000	2.395217000
H	-4.585274000	-1.593996000	3.106456000
H	-3.721033000	-0.421839000	4.113053000
C	-2.554270000	4.512532000	-0.417247000
H	-1.767170000	4.900430000	0.237015000
H	-2.506793000	5.050828000	-1.370731000
H	-2.335244000	3.455083000	-0.601969000
C	2.154828000	6.357539000	-1.995888000
H	1.225499000	6.893095000	-1.772813000
H	2.268823000	6.329930000	-3.084863000
H	2.985959000	6.937807000	-1.581580000
C	-4.244735000	6.178976000	0.423279000
H	-5.210456000	6.325005000	0.918072000
H	-4.282099000	6.679538000	-0.549847000
H	-3.477728000	6.684705000	1.019411000
C	4.502401000	2.008775000	-4.653365000
H	5.389273000	2.636455000	-4.516640000
H	3.714598000	2.625101000	-5.100013000
H	4.755139000	1.218705000	-5.369351000
C	-3.460032000	2.154218000	-3.559838000
H	-3.754944000	2.590661000	-2.600942000
C	3.360546000	2.124095000	3.525838000
H	4.112646000	1.549851000	4.077910000
H	3.059262000	1.545408000	2.644968000
H	2.478770000	2.238906000	4.166509000
C	-5.354180000	-2.359453000	-0.578529000
H	-4.886483000	-3.241225000	-1.029973000
H	-5.942186000	-2.691230000	0.284559000
H	-4.554428000	-1.704018000	-0.219522000
C	-1.690800000	-2.915119000	-2.419028000
H	-1.298855000	-2.148116000	-1.741897000
H	-2.744286000	-3.080433000	-2.172948000
H	-1.643356000	-2.519356000	-3.439249000
C	-1.931935000	2.178423000	-3.620337000
H	-1.570465000	3.208182000	-3.533051000
H	-1.559875000	1.774863000	-4.568637000
H	-1.494108000	1.593476000	-2.804554000
C	4.376373000	4.286176000	4.341138000
H	3.561280000	4.440676000	5.055968000
H	4.767067000	5.268446000	4.055779000
H	5.171079000	3.743644000	4.864118000
C	-1.471006000	-5.298486000	-3.215520000
H	-2.520363000	-5.488682000	-2.965359000
H	-1.431919000	-4.991786000	-4.266490000
H	-0.921476000	-6.240662000	-3.117938000

C	-4.069100000	3.016002000	-4.668154000
H	-3.707984000	4.047207000	-4.588370000
H	-3.792226000	2.638977000	-5.658952000
H	-5.162394000	3.032380000	-4.607478000
Cu	2.506532000	1.253064000	0.077757000
Au	0.404032000	-1.615924000	0.409149000

Complex 6 (E = -4035.98207399; G = -4034.385715)

Au	4.247832000	0.944968000	-0.189517000
Au	-1.035794000	-1.213113000	0.570178000
Au	-3.483344000	1.273206000	-0.115350000
N	-0.947637000	-4.174857000	0.033978000
N	6.885917000	0.134598000	-1.478705000
N	-6.386854000	1.491674000	-1.177044000
C	-0.871329000	-3.173526000	0.938451000
N	-0.638295000	-3.787529000	2.119077000
C	2.293074000	1.075219000	0.029494000
C	6.223216000	-0.643025000	-2.484459000
N	-5.750060000	3.204473000	-0.062447000
C	1.060880000	1.059632000	0.091944000
N	7.230643000	1.389373000	0.220284000
C	6.243034000	0.793369000	-0.485705000
C	5.641398000	0.022601000	-3.571961000
C	-1.552212000	0.858258000	0.116868000
C	-0.762926000	-5.403867000	0.637892000
H	-0.787852000	-6.325472000	0.077484000
C	-5.353955000	1.966024000	-0.442110000
C	6.991925000	2.208083000	1.371475000
C	-1.188654000	-3.947813000	-1.361311000
C	-0.570592000	-5.158696000	1.959119000
H	-0.391194000	-5.822013000	2.790925000
C	6.187292000	-2.038903000	-2.329586000
C	-0.297365000	0.915991000	0.136913000
C	-2.514862000	-3.989778000	-1.818311000
C	-1.613013000	-2.696085000	4.067136000
C	-5.732133000	0.004958000	-2.999601000
C	5.711102000	1.526643000	-3.745269000
H	6.237414000	1.947876000	-2.883324000
C	-4.922614000	4.095863000	0.695640000
C	-6.447972000	0.208374000	-1.812099000
C	8.253556000	0.323583000	-1.400359000
H	8.932856000	-0.121892000	-2.110478000
C	-7.406500000	2.424774000	-1.263022000
H	-8.313409000	2.226108000	-1.812960000
C	-5.864215000	3.040406000	2.818929000
H	-6.570621000	2.626460000	2.091429000
C	-7.004074000	3.507212000	-0.553713000
H	-7.484195000	4.452557000	-0.353968000
C	4.989630000	-0.761414000	-4.528853000

H	4.525747000	-0.277703000	-5.383793000
C	-4.975795000	4.032031000	2.095276000
C	-4.072045000	4.963726000	-0.003758000
C	-0.098171000	-3.646484000	-2.187936000
C	-3.245377000	5.796883000	0.753469000
H	-2.567731000	6.478029000	0.246349000
C	-4.848623000	1.067278000	-3.621853000
H	-4.832301000	1.934604000	-2.955234000
C	1.326139000	-3.589040000	-1.674091000
H	1.315814000	-3.837991000	-0.607922000
C	-1.675045000	-3.395124000	-4.013151000
H	-1.867626000	-3.171915000	-5.059038000
C	-0.469421000	-3.086185000	3.357629000
C	5.531041000	-2.775573000	-3.316540000
H	5.480120000	-3.856380000	-3.232958000
C	6.962407000	1.594334000	2.631549000
C	-3.272308000	5.757050000	2.142765000
H	-2.618633000	6.410210000	2.714265000
C	-3.666394000	-4.271172000	-0.874453000
H	-3.266094000	-4.810412000	-0.008944000
C	-7.285156000	-0.767475000	-1.241331000
C	-3.017916000	-2.983666000	3.577640000
H	-2.946239000	-3.555467000	2.646730000
C	6.772157000	3.579593000	1.178517000
C	1.891283000	-2.174137000	-1.809629000
H	1.966458000	-1.874627000	-2.860700000
H	2.893407000	-2.114185000	-1.373606000
H	1.253705000	-1.449054000	-1.294650000
C	-4.264604000	-2.956945000	-0.365221000
H	-4.675091000	-2.370017000	-1.192361000
H	-3.509922000	-2.343735000	0.137528000
H	-5.073307000	-3.158916000	0.344108000
C	4.935257000	-2.143248000	-4.403782000
H	4.427018000	-2.735270000	-5.160028000
C	-5.023402000	1.880056000	3.358634000
H	-4.309775000	2.233180000	4.111886000
H	-4.452834000	1.402877000	2.553827000
H	-5.664894000	1.124383000	3.824814000
C	-0.372339000	-3.371651000	-3.530650000
H	0.447960000	-3.129480000	-4.200402000
C	0.837046000	-2.801309000	3.786088000
C	-5.414041000	1.529817000	-4.966016000
H	-4.792771000	2.330387000	-5.381786000
H	-6.434582000	1.912048000	-4.858517000
H	-5.435701000	0.710679000	-5.693331000
C	6.509757000	1.894293000	-4.997274000
H	7.522026000	1.478205000	-4.960840000
H	6.592088000	2.982722000	-5.087962000
H	6.022796000	1.519062000	-5.904053000
C	-1.417819000	-1.996848000	5.261436000

H	-2.282867000	-1.680559000	5.837600000
C	2.046562000	-3.164263000	2.947448000
H	1.763680000	-3.987471000	2.282092000
C	6.773896000	-2.714239000	-1.105021000
H	7.574373000	-2.075134000	-0.717376000
C	-4.128224000	4.884581000	2.806338000
H	-4.133449000	4.859120000	3.892121000
C	8.472181000	1.116158000	-0.321711000
H	9.381416000	1.507257000	0.107463000
C	4.313757000	2.147257000	-3.776560000
H	3.737911000	1.791011000	-4.637817000
H	4.384929000	3.237802000	-3.849871000
H	3.754500000	1.900532000	-2.867278000
C	6.785172000	4.211110000	-0.199139000
H	7.157095000	3.465240000	-0.909373000
C	-4.362178000	6.382645000	-2.059326000
H	-3.636049000	7.134468000	-1.731635000
H	-5.354002000	6.703289000	-1.723591000
H	-4.360578000	6.373335000	-3.154554000
C	-4.012063000	4.996036000	-1.517654000
H	-4.757083000	4.293635000	-1.904894000
C	-4.743231000	-5.155580000	-1.498843000
H	-5.498860000	-5.404981000	-0.746614000
H	-4.323514000	-6.091088000	-1.882980000
H	-5.259319000	-4.650026000	-2.320913000
C	-2.643200000	4.531793000	-2.019028000
H	-2.629717000	4.502019000	-3.114228000
H	-2.408166000	3.529352000	-1.644314000
H	-1.849133000	5.211090000	-1.689955000
C	6.462175000	3.770335000	3.575893000
H	6.248765000	4.386237000	4.445180000
C	6.691258000	2.408112000	3.733944000
H	6.653137000	1.967449000	4.726218000
C	-2.735548000	-3.702927000	-3.166332000
H	-3.748805000	-3.712114000	-3.555014000
C	-0.138549000	-1.701146000	5.714662000
H	-0.007042000	-1.154919000	6.644618000
C	-6.683222000	3.695681000	3.929855000
H	-6.044913000	4.080026000	4.732379000
H	-7.364430000	2.962781000	4.375019000
H	-7.282663000	4.527905000	3.546673000
C	-5.884127000	-1.232526000	-3.634125000
H	-5.346923000	-1.422207000	-4.559221000
C	2.212034000	-4.617315000	-2.378524000
H	1.813412000	-5.631261000	-2.266653000
H	3.221628000	-4.599881000	-1.954687000
H	2.297002000	-4.403236000	-3.449490000
C	-3.795120000	-3.833738000	4.583654000
H	-3.924218000	-3.307514000	5.535693000
H	-3.281119000	-4.778738000	4.788095000

H	-4.791703000	-4.065465000	4.192889000
C	7.169658000	0.103538000	2.807219000
H	7.492028000	-0.309327000	1.845828000
C	0.976728000	-2.097383000	4.983157000
H	1.969813000	-1.849100000	5.344363000
C	-7.410993000	-1.978718000	-1.921196000
H	-8.050651000	-2.756700000	-1.517633000
C	6.503080000	4.349504000	2.312346000
H	6.318807000	5.414282000	2.202057000
C	-3.407717000	0.574399000	-3.762951000
H	-3.340161000	-0.278138000	-4.447449000
H	-3.000362000	0.266328000	-2.793709000
H	-2.772105000	1.373831000	-4.158997000
C	2.437723000	-1.970791000	2.070987000
H	1.607391000	-1.657581000	1.431133000
H	3.287558000	-2.223770000	1.429440000
H	2.721489000	-1.113285000	2.690565000
C	5.704821000	-2.826680000	-0.013394000
H	4.890396000	-3.483272000	-0.340471000
H	5.274663000	-1.848293000	0.222856000
H	6.133651000	-3.247183000	0.903088000
C	3.233181000	-3.641754000	3.781771000
H	3.647313000	-2.838185000	4.399776000
H	4.033813000	-3.988960000	3.120317000
H	2.955919000	-4.469996000	4.442267000
C	-3.757126000	-1.680775000	3.265068000
H	-4.764386000	-1.893097000	2.892429000
H	-3.225690000	-1.098908000	2.503582000
H	-3.853333000	-1.057258000	4.160498000
C	-6.723414000	-2.206382000	-3.110317000
H	-6.842221000	-3.154065000	-3.628230000
C	8.264476000	-0.204610000	3.828073000
H	7.989326000	0.139318000	4.830694000
H	9.210882000	0.273503000	3.555042000
H	8.433328000	-1.285240000	3.884323000
C	-7.974712000	-0.530019000	0.089338000
H	-8.185267000	0.541320000	0.173958000
C	5.366346000	4.590619000	-0.629525000
H	5.372241000	5.002698000	-1.644658000
H	4.943054000	5.347131000	0.040818000
H	4.703500000	3.717685000	-0.615306000
C	-7.033739000	-0.901935000	1.241011000
H	-7.484361000	-0.634339000	2.203156000
H	-6.070439000	-0.389554000	1.157979000
H	-6.843326000	-1.980576000	1.244172000
C	7.388619000	-4.078354000	-1.406283000
H	7.891829000	-4.462476000	-0.513095000
H	8.126635000	-4.019961000	-2.212863000
H	6.629637000	-4.814110000	-1.692602000
C	5.850959000	-0.576863000	3.182119000

H	5.986336000	-1.661297000	3.255144000
H	5.079004000	-0.376134000	2.432538000
H	5.483837000	-0.215536000	4.149638000
C	-9.306082000	-1.263338000	0.223459000
H	-9.171303000	-2.349219000	0.270410000
H	-9.977692000	-1.038899000	-0.611557000
H	-9.803431000	-0.958379000	1.149747000
C	7.724466000	5.415312000	-0.265347000
H	8.742042000	5.145625000	0.035886000
H	7.764613000	5.804511000	-1.288153000
H	7.383847000	6.228831000	0.384027000

TS 6 π,π (E = -4035.95991688; G = -4034.360951)

Au	3.864963000	1.019218000	-0.279910000
Au	0.026267000	-1.085873000	0.564942000
Au	-3.924169000	1.062985000	-0.233732000
N	0.007863000	-4.073954000	0.331547000
N	6.680351000	0.636256000	-1.469953000
N	-6.858934000	0.565830000	-1.063139000
C	0.103766000	-2.983147000	1.122908000
N	0.286442000	-3.468854000	2.371241000
C	1.900876000	0.997868000	-0.087885000
C	6.296608000	-0.385701000	-2.398970000
N	-6.561895000	2.485358000	-0.165121000
C	0.657890000	1.051642000	-0.066756000
N	6.645382000	2.105798000	0.086709000
C	5.861455000	1.207693000	-0.554811000
C	5.549833000	-0.025786000	-3.528913000
C	-1.958710000	0.983157000	-0.080224000
C	0.122943000	-5.235078000	1.074687000
H	0.066108000	-6.210911000	0.618371000
C	-5.914141000	1.338453000	-0.477565000
C	6.157314000	3.004913000	1.090926000
C	-0.292059000	-4.003291000	-1.067692000
C	0.295425000	-4.852827000	2.364423000
H	0.424718000	-5.425230000	3.269751000
C	6.729750000	-1.700152000	-2.149631000
C	-0.716765000	1.043286000	-0.065014000
C	-1.643011000	-4.030081000	-1.444283000
C	-0.759789000	-2.109139000	4.098663000
C	-6.036432000	-0.907546000	-2.825105000
C	5.134253000	1.402234000	-3.820365000
H	5.430822000	2.027209000	-2.972496000
C	-5.919601000	3.607632000	0.451694000
C	-6.651890000	-0.756743000	-1.575428000
C	7.951191000	1.180804000	-1.410542000
H	8.740591000	0.857391000	-2.071105000
C	-8.075036000	1.224579000	-1.122407000
H	-8.948487000	0.766902000	-1.560336000

C	-6.420499000	2.551230000	2.722733000
H	-6.989621000	1.869684000	2.082094000
C	-7.887224000	2.438540000	-0.549422000
H	-8.561668000	3.263571000	-0.381032000
C	5.218837000	-1.045177000	-4.427888000
H	4.642499000	-0.802268000	-5.316159000
C	-5.850049000	3.651201000	1.850977000
C	-5.362541000	4.592146000	-0.377133000
C	0.758991000	-3.862693000	-1.983746000
C	-4.712843000	5.659817000	0.246258000
H	-4.263355000	6.438861000	-0.363024000
C	-5.534221000	0.265550000	-3.642012000
H	-5.727639000	1.183551000	-3.079076000
C	2.208207000	-3.772595000	-1.551906000
H	2.251559000	-3.939339000	-0.470455000
C	-0.911968000	-3.783338000	-3.741412000
H	-1.155410000	-3.693300000	-4.796577000
C	0.408516000	-2.636538000	3.531424000
C	6.375609000	-2.677740000	-3.080026000
H	6.688838000	-3.705196000	-2.925701000
C	6.130698000	2.574324000	2.427264000
C	-4.624993000	5.731873000	1.632107000
H	-4.111870000	6.568696000	2.097843000
C	-2.759508000	-4.107744000	-0.423658000
H	-2.315203000	-4.318987000	0.554308000
C	-7.131615000	-1.840276000	-0.817617000
C	-2.143048000	-2.425692000	3.565395000
H	-2.030377000	-2.994361000	2.637130000
C	5.705397000	4.265529000	0.678157000
C	2.752948000	-2.369459000	-1.828363000
H	2.758480000	-2.154988000	-2.902023000
H	3.780169000	-2.272918000	-1.462083000
H	2.142499000	-1.606858000	-1.335385000
C	-3.466049000	-2.754605000	-0.321152000
H	-3.957984000	-2.493168000	-1.263052000
H	-2.754365000	-1.959407000	-0.080092000
H	-4.228602000	-2.780706000	0.462431000
C	5.625797000	-2.353593000	-4.207242000
H	5.361251000	-3.130121000	-4.919610000
C	-5.288789000	1.744697000	3.363928000
H	-4.696901000	2.370218000	4.041823000
H	-4.613249000	1.344489000	2.599975000
H	-5.694118000	0.907337000	3.942195000
C	0.417307000	-3.757894000	-3.334889000
H	1.205294000	-3.643825000	-4.074016000
C	1.695408000	-2.366957000	4.024701000
C	-6.286547000	0.373667000	-4.969812000
H	-5.950591000	1.257049000	-5.523326000
H	-7.366228000	0.462323000	-4.809937000
H	-6.109418000	-0.503549000	-5.601569000

C	5.857740000	1.931960000	-5.060146000
H	6.944970000	1.876166000	-4.941935000
H	5.588110000	2.978596000	-5.238425000
H	5.585583000	1.358460000	-5.953154000
C	-0.609252000	-1.275347000	5.210630000
H	-1.491985000	-0.851423000	5.680739000
C	2.930874000	-2.911743000	3.336053000
H	2.651672000	-3.846116000	2.836306000
C	7.501616000	-2.046846000	-0.889972000
H	8.123925000	-1.184306000	-0.627784000
C	-5.187115000	4.738071000	2.425408000
H	-5.105744000	4.800581000	3.506863000
C	7.929444000	2.109021000	-0.423176000
H	8.692930000	2.771899000	-0.047206000
C	3.617352000	1.523846000	-3.968908000
H	3.252498000	0.958297000	-4.833200000
H	3.334508000	2.572097000	-4.113052000
H	3.103522000	1.152417000	-3.075651000
C	5.682716000	4.677039000	-0.780677000
H	6.229459000	3.923775000	-1.357028000
C	-6.017693000	5.756459000	-2.514887000
H	-5.398082000	6.639079000	-2.323591000
H	-7.019896000	5.955505000	-2.121392000
H	-6.095899000	5.636339000	-3.600601000
C	-5.412889000	4.499798000	-1.889156000
H	-6.057447000	3.656531000	-2.157608000
C	-3.740882000	-5.238399000	-0.728740000
H	-4.515684000	-5.282959000	0.044176000
H	-3.233079000	-6.208311000	-0.757776000
H	-4.242887000	-5.090042000	-1.689656000
C	-4.020595000	4.212011000	-2.456280000
H	-4.068379000	4.096060000	-3.544806000
H	-3.603740000	3.292186000	-2.030057000
H	-3.328573000	5.031801000	-2.232807000
C	5.197301000	4.739154000	2.999346000
H	4.818832000	5.422879000	3.754215000
C	5.643558000	3.475542000	3.375208000
H	5.603783000	3.184461000	4.419792000
C	-1.932260000	-3.915054000	-2.805425000
H	-2.968578000	-3.913979000	-3.131507000
C	0.649829000	-0.984264000	5.719651000
H	0.745457000	-0.331271000	6.582771000
C	-7.381560000	3.099958000	3.777146000
H	-6.870913000	3.766041000	4.480723000
H	-7.815854000	2.277678000	4.355625000
H	-8.201174000	3.660805000	3.316018000
C	-5.911361000	-2.208436000	-3.324423000
H	-5.445051000	-2.361391000	-4.293697000
C	3.068484000	-4.843942000	-2.222048000
H	2.686634000	-5.849839000	-2.017961000

H	4.097438000	-4.788471000	-1.851320000
H	3.101045000	-4.708656000	-3.308475000
C	-2.916977000	-3.300361000	4.553943000
H	-3.072067000	-2.777977000	5.504492000
H	-2.384293000	-4.233439000	4.765732000
H	-3.901183000	-3.554969000	4.145546000
C	6.624630000	1.195740000	2.821037000
H	6.422540000	0.522984000	1.979676000
C	1.790444000	-1.524885000	5.133510000
H	2.766816000	-1.283981000	5.541952000
C	-6.985599000	-3.115501000	-1.363323000
H	-7.344710000	-3.978699000	-0.812511000
C	5.223301000	5.128348000	1.666241000
H	4.856565000	6.110898000	1.383549000
C	-4.023401000	0.173748000	-3.865224000
H	-3.757321000	-0.722914000	-4.436115000
H	-3.485098000	0.142464000	-2.911314000
H	-3.670060000	1.046120000	-4.425701000
C	3.402398000	-1.936690000	2.253020000
H	2.610829000	-1.721133000	1.529259000
H	4.255420000	-2.357882000	1.714028000
H	3.712861000	-0.983872000	2.694912000
C	6.527510000	-2.274494000	0.269364000
H	5.892071000	-3.144827000	0.069673000
H	5.876995000	-1.407895000	0.418305000
H	7.072243000	-2.457902000	1.202462000
C	4.066962000	-3.236600000	4.302281000
H	4.479118000	-2.334048000	4.765406000
H	4.883813000	-3.723424000	3.759374000
H	3.740224000	-3.910419000	5.101088000
C	-2.920809000	-1.153015000	3.230540000
H	-3.893610000	-1.408507000	2.798459000
H	-2.380451000	-0.536470000	2.505335000
H	-3.102217000	-0.547608000	4.124955000
C	-6.387616000	-3.297502000	-2.607395000
H	-6.292847000	-4.298884000	-3.017975000
C	8.138501000	1.209718000	3.056418000
H	8.392553000	1.876023000	3.888556000
H	8.685234000	1.550989000	2.172415000
H	8.494711000	0.204114000	3.305587000
C	-7.743191000	-1.630680000	0.555755000
H	-8.238916000	-0.653954000	0.556017000
C	4.245043000	4.700159000	-1.306597000
H	4.233413000	4.950680000	-2.373177000
H	3.644788000	5.448086000	-0.776312000
H	3.761492000	3.724989000	-1.176092000
C	-6.648712000	-1.586076000	1.627731000
H	-7.081649000	-1.338323000	2.603209000
H	-5.882105000	-0.841358000	1.392797000
H	-6.157823000	-2.562059000	1.714437000

C	8.434048000	-3.242551000	-1.056742000
H	9.046490000	-3.359034000	-0.157002000
H	9.106686000	-3.116516000	-1.911255000
H	7.879520000	-4.176799000	-1.194372000
C	5.903285000	0.627163000	4.040502000
H	6.190053000	-0.420065000	4.180686000
H	4.815829000	0.671090000	3.924246000
H	6.170207000	1.162871000	4.958019000
C	-8.800752000	-2.671494000	0.912098000
H	-8.363558000	-3.665784000	1.052666000
H	-9.573446000	-2.745057000	0.139980000
H	-9.286867000	-2.395682000	1.853349000
C	6.378224000	6.018720000	-1.008693000
H	7.409551000	6.001500000	-0.641307000
H	6.401858000	6.252013000	-2.078390000
H	5.853197000	6.836884000	-0.504438000

Complex 6-fw (E = -4035.98101204; G = -4034.384362)

Au	3.498810000	1.196245000	-0.386450000
Au	1.079871000	-1.353500000	0.304305000
Au	-4.230868000	0.907513000	-0.375196000
N	0.652136000	-4.325690000	0.082400000
N	6.383621000	1.696095000	-1.320285000
N	-7.175007000	0.050014000	-0.536809000
C	0.899255000	-3.262876000	0.879530000
N	0.953898000	-3.771385000	2.130637000
C	1.581751000	0.674074000	-0.337408000
C	6.364242000	0.651019000	-2.300434000
N	-6.963940000	2.036778000	0.227314000
C	0.326787000	0.695795000	-0.390059000
N	5.795433000	3.020182000	0.256026000
C	5.363759000	1.964614000	-0.472642000
C	5.642293000	0.852524000	-3.484634000
C	-2.263390000	0.880002000	-0.473269000
C	0.548523000	-5.487639000	0.822262000
H	0.350171000	-6.442801000	0.361184000
C	-6.246204000	0.981490000	-0.222186000
C	4.986190000	3.678593000	1.238839000
C	0.462846000	-4.209731000	-1.333447000
C	0.736021000	-5.136284000	2.120118000
H	0.738201000	-5.721683000	3.026454000
C	7.082393000	-0.525017000	-2.027351000
C	-1.030821000	0.830549000	-0.460012000
C	-0.849246000	-4.104279000	-1.817434000
C	0.126696000	-2.245799000	3.840799000
C	-6.648392000	-1.403128000	-2.421045000
C	4.916213000	2.147057000	-3.791525000
H	4.973238000	2.789881000	-2.907898000
C	-6.366436000	3.258227000	0.678573000

C	-6.876807000	-1.255154000	-1.046667000
C	7.434630000	2.575364000	-1.129651000
H	8.337644000	2.519880000	-1.717542000
C	-8.454395000	0.516750000	-0.293912000
H	-9.327440000	-0.086579000	-0.488590000
C	-6.087674000	2.186836000	2.984565000
H	-6.625043000	1.378669000	2.478151000
C	-8.319863000	1.774669000	0.194356000
H	-9.050493000	2.499932000	0.516930000
C	5.641841000	-0.189162000	-4.417847000
H	5.095163000	-0.066948000	-5.348604000
C	-5.938545000	3.338508000	2.010866000
C	-6.209942000	4.303531000	-0.243535000
C	1.593881000	-4.151661000	-2.159497000
C	-5.597074000	5.472350000	0.212650000
H	-5.451689000	6.300890000	-0.474244000
C	-6.662527000	-0.230772000	-3.380607000
H	-6.943931000	0.666369000	-2.820105000
C	3.005696000	-4.242643000	-1.616685000
H	2.945767000	-4.412198000	-0.536641000
C	0.088556000	-3.873933000	-4.041186000
H	-0.058573000	-3.737458000	-5.109022000
C	1.195159000	-2.967747000	3.293014000
C	7.052414000	-1.531649000	-2.992743000
H	7.589159000	-2.458403000	-2.816236000
C	4.996397000	3.193538000	2.553735000
C	-5.162953000	5.584646000	1.529232000
H	-4.686320000	6.501803000	1.864080000
C	-2.057138000	-4.126997000	-0.902980000
H	-1.713379000	-4.338199000	0.114625000
C	-6.846598000	-2.328977000	-0.142475000
C	-1.286527000	-2.337401000	3.301371000
H	-1.272639000	-2.973420000	2.410762000
C	4.190887000	4.757541000	0.824222000
C	3.759817000	-2.930920000	-1.840586000
H	3.853244000	-2.705555000	-2.908088000
H	4.769421000	-2.993806000	-1.422887000
H	3.244709000	-2.089518000	-1.363282000
C	-2.738477000	-2.757061000	-0.876860000
H	-3.116321000	-2.478733000	-1.866727000
H	-2.041375000	-1.978757000	-0.553771000
H	-3.587111000	-2.763166000	-0.185422000
C	6.337341000	-1.366415000	-4.175466000
H	6.325617000	-2.163242000	-4.914067000
C	-4.715385000	1.644479000	3.390920000
H	-4.133988000	2.403672000	3.926157000
H	-4.139967000	1.337022000	2.510838000
H	-4.827166000	0.777664000	4.051273000
C	1.376671000	-3.980919000	-3.528944000
H	2.229883000	-3.924888000	-4.199265000

C	2.502060000	-2.919441000	3.804244000
C	-7.697031000	-0.424933000	-4.489626000
H	-7.724339000	0.457150000	-5.138205000
H	-8.700073000	-0.575233000	-4.077053000
H	-7.456139000	-1.290490000	-5.116093000
C	5.598778000	2.885655000	-4.945092000
H	6.650478000	3.091854000	-4.720208000
H	5.097081000	3.840936000	-5.133741000
H	5.560523000	2.297852000	-5.869007000
C	0.406260000	-1.428013000	4.939951000
H	-0.397895000	-0.853008000	5.390515000
C	3.627237000	-3.700263000	3.154384000
H	3.188097000	-4.582029000	2.675258000
C	7.807583000	-0.716016000	-0.709264000
H	8.083538000	0.273224000	-0.329269000
C	-5.331892000	4.529310000	2.417799000
H	-4.982936000	4.624012000	3.442341000
C	7.062991000	3.411616000	-0.128623000
H	7.572700000	4.239169000	0.339638000
C	3.435272000	1.908248000	-4.087234000
H	3.298745000	1.304463000	-4.990782000
H	2.923374000	2.863556000	-4.244818000
H	2.944635000	1.392322000	-3.254809000
C	4.156495000	5.232055000	-0.614988000
H	4.937498000	4.701299000	-1.168979000
C	-7.445866000	5.357979000	-2.181175000
H	-6.853434000	6.278973000	-2.188047000
H	-8.324923000	5.525427000	-1.550307000
H	-7.790936000	5.182624000	-3.205510000
C	-6.627699000	4.163085000	-1.693861000
H	-7.262329000	3.274823000	-1.779497000
C	-3.031734000	-5.237384000	-1.294724000
H	-3.868481000	-5.272063000	-0.589518000
H	-2.541317000	-6.216686000	-1.290712000
H	-3.446748000	-5.071410000	-2.294297000
C	-5.398559000	3.937436000	-2.579124000
H	-5.700983000	3.780384000	-3.620455000
H	-4.827572000	3.061828000	-2.249990000
H	-4.730218000	4.805494000	-2.547486000
C	3.373613000	4.910137000	3.101925000
H	2.735979000	5.394383000	3.836373000
C	4.170840000	3.835990000	3.479688000
H	4.148817000	3.482771000	4.506715000
C	-1.012323000	-3.936605000	-3.194204000
H	-2.014935000	-3.842435000	-3.602056000
C	1.691470000	-1.344569000	5.459789000
H	1.889294000	-0.700232000	6.311987000
C	-6.911349000	2.592003000	4.207481000
H	-6.416940000	3.386369000	4.776933000
H	-7.042677000	1.734923000	4.876560000

H	-7.903853000	2.952591000	3.917697000
C	-6.385874000	-2.693131000	-2.891767000
H	-6.200943000	-2.844410000	-3.951601000
C	3.762229000	-5.423128000	-2.227587000
H	3.238224000	-6.368215000	-2.050684000
H	4.761948000	-5.499999000	-1.786671000
H	3.884718000	-5.302472000	-3.309418000
C	-2.214230000	-2.995038000	4.325058000
H	-2.281826000	-2.395366000	5.239579000
H	-1.860563000	-3.993730000	4.602290000
H	-3.224475000	-3.093190000	3.913099000
C	5.829214000	1.999589000	2.972544000
H	6.430565000	1.685329000	2.113377000
C	2.727999000	-2.087200000	4.901133000
H	3.725349000	-2.015891000	5.322812000
C	-6.588668000	-3.597551000	-0.663974000
H	-6.559257000	-4.453986000	0.002508000
C	3.383594000	5.365307000	1.788026000
H	2.748781000	6.199260000	1.502372000
C	-5.264793000	0.004523000	-3.957096000
H	-4.930027000	-0.859057000	-4.542825000
H	-4.535161000	0.175748000	-3.157400000
H	-5.264766000	0.879737000	-4.615800000
C	4.293708000	-2.862877000	2.059554000
H	3.564408000	-2.480828000	1.337133000
H	5.033962000	-3.462418000	1.519645000
H	4.807379000	-2.002299000	2.499241000
C	6.865223000	-1.356062000	0.313650000
H	6.599799000	-2.372593000	0.003986000
H	5.937477000	-0.784778000	0.417503000
H	7.345843000	-1.415800000	1.296427000
C	4.666372000	-4.201484000	4.154265000
H	5.235008000	-3.378077000	4.599397000
H	5.384588000	-4.852722000	3.645547000
H	4.204697000	-4.773960000	4.965327000
C	-1.814782000	-0.966994000	2.877072000
H	-2.807437000	-1.067169000	2.427035000
H	-1.155983000	-0.499876000	2.138929000
H	-1.900975000	-0.288933000	3.733326000
C	-6.364689000	-3.778386000	-2.025429000
H	-6.168185000	-4.774570000	-2.411484000
C	6.793193000	2.355404000	4.104812000
H	6.254714000	2.649273000	5.012272000
H	7.452657000	3.182342000	3.821616000
H	7.418763000	1.491738000	4.354771000
C	-7.009808000	-2.113578000	1.349179000
H	-7.542266000	-1.168440000	1.499136000
C	2.814501000	4.880920000	-1.262656000
H	2.809380000	5.181192000	-2.316328000
H	1.989035000	5.397111000	-0.759767000

H	2.620424000	3.803513000	-1.208899000
C	-5.629994000	-1.973535000	2.001061000
H	-5.729599000	-1.748386000	3.068690000
H	-5.049899000	-1.172461000	1.530401000
H	-5.062740000	-2.906018000	1.899919000
C	9.096239000	-1.523397000	-0.841723000
H	9.629724000	-1.528008000	0.114286000
H	9.762486000	-1.099293000	-1.599926000
H	8.898864000	-2.567205000	-1.107582000
C	4.927786000	0.824737000	3.359206000
H	5.534879000	-0.049760000	3.617246000
H	4.259834000	0.551007000	2.535335000
H	4.306236000	1.071153000	4.227219000
C	-7.826773000	-3.210035000	2.028202000
H	-7.305022000	-4.172855000	2.018336000
H	-8.799776000	-3.345607000	1.544987000
H	-8.001842000	-2.946890000	3.076450000
C	4.451877000	6.727583000	-0.727761000
H	5.415881000	6.979596000	-0.273781000
H	4.482894000	7.026750000	-1.780735000
H	3.679173000	7.328198000	-0.236110000

TS 6 σ,π (E = -4035.97936373; G = -4034.382911)

Au	3.226890000	1.288949000	-0.499356000
Au	1.554569000	-1.227313000	0.272572000
Au	-4.460673000	0.784986000	-0.474460000
N	1.336359000	-4.223792000	0.215465000
N	6.105507000	2.080989000	-1.306779000
N	-7.219728000	-0.509646000	-0.540348000
C	1.519122000	-3.110826000	0.962480000
N	1.516400000	-3.551297000	2.241936000
C	1.307861000	0.713472000	-0.507543000
C	6.291933000	1.013058000	-2.243336000
N	-7.304812000	1.413625000	0.392858000
C	0.072869000	0.869824000	-0.620445000
N	5.249032000	3.368580000	0.174402000
C	5.009856000	2.236451000	-0.528307000
C	5.607408000	1.063917000	-3.464773000
C	-2.511055000	0.963763000	-0.667913000
C	1.219180000	-5.346947000	1.012642000
H	1.069421000	-6.330521000	0.595272000
C	-6.449481000	0.547923000	-0.196512000
C	4.297567000	3.932714000	1.085177000
C	1.195950000	-4.194142000	-1.210099000
C	1.328979000	-4.921221000	2.295424000
H	1.295723000	-5.455838000	3.231980000
C	7.173594000	-0.025418000	-1.896213000
C	-1.275181000	0.977278000	-0.684653000
C	-0.098078000	-4.108631000	-1.744576000

C	0.533616000	-1.954683000	3.796282000
C	-6.548662000	-1.695512000	-2.561295000
C	4.704065000	2.216848000	-3.853861000
H	4.633497000	2.900601000	-3.002695000
C	-6.901106000	2.680872000	0.925344000
C	-6.712781000	-1.692656000	-1.169770000
C	7.012335000	3.105708000	-1.098391000
H	7.947745000	3.155327000	-1.633866000
C	-8.538046000	-0.308574000	-0.176054000
H	-9.302647000	-1.044595000	-0.369735000
C	-6.230325000	1.481834000	3.083543000
H	-6.638596000	0.631493000	2.527756000
C	-8.591300000	0.909605000	0.418497000
H	-9.412120000	1.460118000	0.851151000
C	5.814982000	0.008497000	-4.358637000
H	5.300141000	0.015312000	-5.315125000
C	-6.382306000	2.722771000	2.226876000
C	-7.017534000	3.814307000	0.106985000
C	2.353713000	-4.204786000	-1.999669000
C	-6.594710000	5.033856000	0.639310000
H	-6.662089000	5.932803000	0.033907000
C	-6.886353000	-0.495626000	-3.422226000
H	-7.322438000	0.274393000	-2.777973000
C	3.742580000	-4.258011000	-1.398281000
H	3.640993000	-4.412369000	-0.318978000
C	0.915793000	-4.040160000	-3.945690000
H	0.805815000	-3.976110000	-5.024728000
C	1.652267000	-2.687257000	3.376619000
C	7.351670000	-1.048673000	-2.827453000
H	8.021582000	-1.871401000	-2.599595000
C	4.299288000	3.489364000	2.414620000
C	-6.079104000	5.110032000	1.928827000
H	-5.753821000	6.068362000	2.324116000
C	-1.336165000	-4.065590000	-0.872374000
H	-1.022400000	-4.170564000	0.171207000
C	-6.389361000	-2.788688000	-0.354294000
C	-0.835043000	-2.111933000	3.163469000
H	-0.738967000	-2.775394000	2.298245000
C	3.378532000	4.868191000	0.587414000
C	4.469409000	-2.929477000	-1.612833000
H	4.591066000	-2.715412000	-2.679797000
H	5.466251000	-2.963002000	-1.162777000
H	3.917962000	-2.095081000	-1.163889000
C	-2.042802000	-2.714925000	-1.001102000
H	-2.410164000	-2.550485000	-2.020027000
H	-1.364362000	-1.893820000	-0.750548000
H	-2.902699000	-2.664995000	-0.325654000
C	6.676673000	-1.033739000	-4.045091000
H	6.828499000	-1.842179000	-4.755002000
C	-4.751944000	1.190940000	3.352259000

H	-4.291480000	1.995967000	3.935847000
H	-4.194921000	1.092760000	2.414106000
H	-4.642468000	0.259471000	3.918204000
C	2.184902000	-4.123947000	-3.383939000
H	3.059951000	-4.119477000	-4.027508000
C	2.907652000	-2.605714000	4.001340000
C	-7.924917000	-0.843877000	-4.489011000
H	-8.194269000	0.051358000	-5.059622000
H	-8.837954000	-1.247904000	-4.039424000
H	-7.540568000	-1.585528000	-5.197548000
C	5.302525000	2.998759000	-5.025317000
H	6.301941000	3.375760000	-4.783857000
H	4.666535000	3.855151000	-5.274227000
H	5.386180000	2.371616000	-5.919846000
C	0.710860000	-1.080522000	4.873163000
H	-0.133297000	-0.494242000	5.224706000
C	4.086106000	-3.415934000	3.495721000
H	3.690945000	-4.342760000	3.065397000
C	7.860787000	-0.051905000	-0.543640000
H	8.016199000	0.984872000	-0.226841000
C	-5.973237000	3.967302000	2.712552000
H	-5.562642000	4.037127000	3.715924000
C	6.471050000	3.918225000	-0.157156000
H	6.834553000	4.824378000	0.301822000
C	3.287527000	1.737616000	-4.172534000
H	3.275132000	1.070752000	-5.041552000
H	2.641175000	2.592677000	-4.398066000
H	2.854618000	1.199951000	-3.321627000
C	3.375104000	5.309719000	-0.862527000
H	4.218798000	4.828738000	-1.367739000
C	-8.532647000	4.807095000	-1.661680000
H	-8.083816000	5.805707000	-1.640769000
H	-9.375233000	4.799700000	-0.962581000
H	-8.926906000	4.647533000	-2.670710000
C	-7.514149000	3.720314000	-1.322028000
H	-8.014232000	2.753985000	-1.444511000
C	-2.276492000	-5.229178000	-1.187582000
H	-3.134550000	-5.218048000	-0.507902000
H	-1.768043000	-6.193080000	-1.078665000
H	-2.661574000	-5.163903000	-2.210729000
C	-6.328629000	3.751618000	-2.291365000
H	-6.674245000	3.628120000	-3.323658000
H	-5.613580000	2.951723000	-2.067929000
H	-5.796413000	4.707188000	-2.222854000
C	2.404533000	4.950258000	2.806722000
H	1.655590000	5.349614000	3.485013000
C	3.329365000	4.020417000	3.268079000
H	3.296340000	3.693915000	4.303724000
C	-0.213291000	-4.034353000	-3.134307000
H	-1.200430000	-3.960833000	-3.582124000

C	1.943909000	-0.961569000	5.500708000
H	2.061522000	-0.276069000	6.335508000
C	-7.019138000	1.599358000	4.388097000
H	-6.643502000	2.417885000	5.011702000
H	-6.931606000	0.673454000	4.966588000
H	-8.081749000	1.781545000	4.196628000
C	-6.034896000	-2.858193000	-3.142795000
H	-5.887874000	-2.894123000	-4.218613000
C	4.557785000	-5.425932000	-1.954677000
H	4.044463000	-6.380867000	-1.801331000
H	5.530055000	-5.478064000	-1.453122000
H	4.745350000	-5.311567000	-3.027804000
C	-1.804296000	-2.772243000	4.146899000
H	-1.955305000	-2.144278000	5.032120000
H	-1.433061000	-3.745950000	4.483789000
H	-2.779737000	-2.924340000	3.672568000
C	5.281441000	2.454400000	2.921309000
H	5.974465000	2.214490000	2.108459000
C	3.029097000	-1.723280000	5.075387000
H	3.982273000	-1.625524000	5.584840000
C	-5.888862000	-3.928769000	-0.985156000
H	-5.626465000	-4.797943000	-0.389908000
C	2.430107000	5.370028000	1.481235000
H	1.697611000	6.091559000	1.130329000
C	-5.618625000	0.090452000	-4.046918000
H	-5.140249000	-0.630778000	-4.718882000
H	-4.893432000	0.365342000	-3.272210000
H	-5.857600000	0.987043000	-4.628991000
C	4.815107000	-2.665688000	2.377366000
H	4.133349000	-2.366104000	1.575288000
H	5.599138000	-3.297355000	1.946374000
H	5.286694000	-1.759985000	2.771042000
C	6.956310000	-0.717316000	0.497989000
H	6.822892000	-1.778760000	0.265400000
H	5.965274000	-0.254592000	0.530272000
H	7.403902000	-0.642266000	1.495044000
C	5.062262000	-3.811173000	4.600312000
H	5.592763000	-2.943027000	5.005796000
H	5.818286000	-4.492919000	4.197487000
H	4.554921000	-4.317533000	5.427837000
C	-1.386673000	-0.778744000	2.659547000
H	-2.337052000	-0.934991000	2.139569000
H	-0.691621000	-0.305137000	1.959666000
H	-1.567387000	-0.081150000	3.484970000
C	-5.712650000	-3.963104000	-2.364791000
H	-5.318574000	-4.859365000	-2.835626000
C	6.109845000	2.992221000	4.088771000
H	5.479469000	3.221785000	4.954695000
H	6.646001000	3.905390000	3.810164000
H	6.848134000	2.246937000	4.403663000

C	-6.508490000	-2.721980000	1.155718000
H	-7.173618000	-1.889253000	1.406678000
C	2.098180000	4.851510000	-1.570055000
H	2.129358000	5.132044000	-2.628718000
H	1.210164000	5.313752000	-1.124821000
H	1.980951000	3.764214000	-1.505120000
C	-5.140534000	-2.419673000	1.776121000
H	-5.231083000	-2.302328000	2.861676000
H	-4.711797000	-1.499707000	1.363898000
H	-4.438583000	-3.238051000	1.579170000
C	9.230824000	-0.723507000	-0.574008000
H	9.725410000	-0.595751000	0.394292000
H	9.877265000	-0.291010000	-1.344476000
H	9.154240000	-1.800436000	-0.757967000
C	4.554557000	1.164835000	3.307383000
H	5.274582000	0.407743000	3.634798000
H	3.986856000	0.758300000	2.463102000
H	3.852342000	1.338538000	4.129878000
C	-7.116120000	-3.986684000	1.758745000
H	-6.460791000	-4.854702000	1.631005000
H	-8.084034000	-4.222626000	1.304863000
H	-7.269313000	-3.849376000	2.834186000
C	3.567516000	6.821589000	-0.986046000
H	4.494288000	7.146504000	-0.501733000
H	3.614439000	7.111887000	-2.041015000
H	2.737171000	7.369105000	-0.527188000

Complex 6-*gem* (E = -4035.97936357; G = -4034.387087)

Au	3.197952000	1.296492000	-0.503241000
Au	1.606004000	-1.219113000	0.265433000
Au	-4.481994000	0.771893000	-0.475152000
N	1.397193000	-4.215759000	0.208483000
N	6.066723000	2.120474000	-1.313192000
N	-7.228144000	-0.551350000	-0.522334000
C	1.585086000	-3.103172000	0.955050000
N	1.582128000	-3.543285000	2.234678000
C	1.280217000	0.706421000	-0.509637000
C	6.262539000	1.056989000	-2.252889000
N	-7.326987000	1.372061000	0.408906000
C	0.048275000	0.875883000	-0.628500000
N	5.199545000	3.394895000	0.173290000
C	4.970305000	2.263307000	-0.533342000
C	5.574519000	1.103259000	-3.472516000
C	-2.534926000	0.967492000	-0.676075000
C	1.277630000	-5.338320000	1.006196000
H	1.124172000	-6.321485000	0.589220000
C	-6.466727000	0.514818000	-0.185488000
C	4.243824000	3.945870000	1.087658000
C	1.248896000	-4.187281000	-1.216411000

C	1.390465000	-4.912654000	2.288665000
H	1.357059000	-5.446976000	3.225376000
C	7.156685000	0.027588000	-1.910422000
C	-1.298989000	0.986548000	-0.694104000
C	-0.047991000	-4.101106000	-1.744064000
C	0.601863000	-1.942287000	3.786727000
C	-6.561495000	-1.738680000	-2.543913000
C	4.657731000	2.247268000	-3.856480000
H	4.581111000	2.927554000	-3.003075000
C	-6.932530000	2.644996000	0.934620000
C	-6.711271000	-1.730421000	-1.150801000
C	6.964304000	3.152539000	-1.101586000
H	7.898657000	3.212971000	-1.637791000
C	-8.545772000	-0.364082000	-0.148680000
H	-9.303578000	-1.108876000	-0.335418000
C	-6.237597000	1.460782000	3.093349000
H	-6.642303000	0.604676000	2.543923000
C	-8.607810000	0.854408000	0.444506000
H	-9.431324000	1.396910000	0.882124000
C	5.791583000	0.052412000	-4.369523000
H	5.274120000	0.055632000	-5.324602000
C	-6.406535000	2.696927000	2.232899000
C	-7.063266000	3.773787000	0.112068000
C	2.402172000	-4.202398000	-2.012534000
C	-6.648545000	4.999394000	0.636931000
H	-6.727102000	5.895201000	0.028112000
C	-6.925255000	-0.548280000	-3.407336000
H	-7.370454000	0.216919000	-2.763561000
C	3.794147000	-4.250589000	-1.418033000
H	3.698657000	-4.419944000	-0.340371000
C	0.953640000	-4.042831000	-3.951102000
H	0.837735000	-3.983011000	-5.029760000
C	1.718100000	-2.680967000	3.370727000
C	7.344139000	-0.990993000	-2.844872000
H	8.024070000	-1.806499000	-2.620939000
C	4.249137000	3.493818000	2.414166000
C	-6.126798000	5.085608000	1.923352000
H	-5.808030000	6.048476000	2.312884000
C	-1.281749000	-4.053258000	-0.865904000
H	-0.962733000	-4.150252000	0.176791000
C	-6.360612000	-2.815867000	-0.332558000
C	-0.763363000	-2.086227000	3.143380000
H	-0.659610000	-2.718872000	2.256429000
C	3.317590000	4.877324000	0.595858000
C	4.505017000	-2.911162000	-1.619077000
H	4.619759000	-2.683789000	-2.684070000
H	5.503982000	-2.938110000	-1.173196000
H	3.945201000	-2.088494000	-1.158757000
C	-1.991248000	-2.704721000	-1.000775000
H	-2.366459000	-2.549123000	-2.018209000

H	-1.312278000	-1.880631000	-0.761876000
H	-2.846109000	-2.650025000	-0.319314000
C	6.665839000	-0.980718000	-4.060762000
H	6.824969000	-1.785684000	-4.773012000
C	-4.754816000	1.183589000	3.352350000
H	-4.296849000	1.994476000	3.929694000
H	-4.204051000	1.086771000	2.410362000
H	-4.633187000	0.255044000	3.920608000
C	2.225716000	-4.126568000	-3.396073000
H	3.097124000	-4.126563000	-4.044451000
C	2.970314000	-2.609993000	4.003028000
C	-7.963966000	-0.918202000	-4.466608000
H	-8.251772000	-0.029813000	-5.038925000
H	-8.867393000	-1.335966000	-4.010208000
H	-7.571292000	-1.655874000	-5.174735000
C	5.245396000	3.039582000	-5.026443000
H	6.240904000	3.427077000	-4.785463000
H	4.599321000	3.889533000	-5.271398000
H	5.334508000	2.416410000	-5.923222000
C	0.777271000	-1.077689000	4.871605000
H	-0.065076000	-0.488013000	5.221691000
C	4.148265000	-3.420500000	3.496715000
H	3.752767000	-4.345571000	3.062971000
C	7.848149000	0.006532000	-0.559810000
H	8.000217000	1.044693000	-0.245713000
C	-6.006227000	3.947130000	2.711192000
H	-5.590565000	4.024820000	3.711911000
C	6.416327000	3.956711000	-0.157011000
H	6.771861000	4.864548000	0.304882000
C	3.246000000	1.753511000	-4.174178000
H	3.239088000	1.090129000	-5.045903000
H	2.589639000	2.602218000	-4.394783000
H	2.820947000	1.207558000	-3.324597000
C	3.310416000	5.327884000	-0.851292000
H	4.158292000	4.857532000	-1.359503000
C	-8.609434000	4.736073000	-1.645725000
H	-8.180242000	5.743266000	-1.626004000
H	-9.447230000	4.710859000	-0.941323000
H	-9.006744000	4.569964000	-2.652509000
C	-7.567176000	3.669628000	-1.313697000
H	-8.048700000	2.693558000	-1.432946000
C	-2.222269000	-5.220274000	-1.167911000
H	-3.076701000	-5.205361000	-0.483773000
H	-1.712239000	-6.182889000	-1.055087000
H	-2.612789000	-5.162346000	-2.189456000
C	-6.389366000	3.724732000	-2.291311000
H	-6.740198000	3.597085000	-3.321342000
H	-5.658249000	2.937421000	-2.075307000
H	-5.874171000	4.689703000	-2.224634000
C	2.343320000	4.937689000	2.815743000

H	1.591509000	5.327079000	3.496655000
C	3.275396000	4.012140000	3.271133000
H	3.245552000	3.679282000	4.304869000
C	-0.170786000	-4.031671000	-3.133435000
H	-1.160365000	-3.958160000	-3.575823000
C	2.006555000	-0.971875000	5.508841000
H	2.122564000	-0.293800000	6.349899000
C	-7.018050000	1.576628000	4.403055000
H	-6.645227000	2.400971000	5.020642000
H	-6.918184000	0.653886000	4.984589000
H	-8.083572000	1.748686000	4.218544000
C	-6.035262000	-2.896308000	-3.124222000
H	-5.899055000	-2.936342000	-4.201311000
C	4.620362000	-5.401308000	-1.993312000
H	4.117301000	-6.363869000	-1.854468000
H	5.593666000	-5.451021000	-1.493551000
H	4.806107000	-5.267985000	-3.064574000
C	-1.731139000	-2.786524000	4.100315000
H	-1.885431000	-2.192436000	5.008147000
H	-1.356265000	-3.770463000	4.401541000
H	-2.705383000	-2.925102000	3.619436000
C	5.240356000	2.464381000	2.914448000
H	5.932588000	2.232216000	2.098673000
C	3.090155000	-1.736037000	5.084112000
H	4.040991000	-1.646897000	5.599446000
C	-5.847966000	-3.951330000	-0.962142000
H	-5.564656000	-4.812368000	-0.364548000
C	2.365325000	5.366019000	1.492921000
H	1.627084000	6.083968000	1.146743000
C	-5.671554000	0.057263000	-4.041932000
H	-5.184684000	-0.658087000	-4.714066000
H	-4.946572000	0.347632000	-3.272761000
H	-5.929662000	0.947257000	-4.625948000
C	4.878369000	-2.666942000	2.381208000
H	4.197616000	-2.366524000	1.578617000
H	5.663969000	-3.296573000	1.950019000
H	5.348117000	-1.761324000	2.777473000
C	6.950610000	-0.660670000	0.486766000
H	6.820469000	-1.723007000	0.256617000
H	5.957787000	-0.202142000	0.523173000
H	7.402536000	-0.582062000	1.481574000
C	5.123193000	-3.819956000	4.600777000
H	5.654005000	-2.953553000	5.009544000
H	5.879033000	-4.500955000	4.196367000
H	4.614812000	-4.328648000	5.426233000
C	-1.323720000	-0.741260000	2.682139000
H	-2.266187000	-0.889319000	2.145680000
H	-0.626674000	-0.234118000	2.008269000
H	-1.522771000	-0.076329000	3.529862000
C	-5.686270000	-3.991125000	-2.343355000

H	-5.282458000	-4.883528000	-2.813290000
C	6.068243000	3.005570000	4.080767000
H	5.438409000	3.228339000	4.948863000
H	6.596660000	3.923436000	3.802795000
H	6.813040000	2.265135000	4.391752000
C	-6.465224000	-2.743847000	1.178287000
H	-7.126695000	-1.909400000	1.432988000
C	2.037478000	4.862991000	-1.561683000
H	2.065967000	5.150653000	-2.618509000
H	1.145521000	5.314446000	-1.113290000
H	1.929660000	3.774350000	-1.503934000
C	-5.091508000	-2.441952000	1.785670000
H	-5.172207000	-2.322482000	2.871755000
H	-4.665960000	-1.522971000	1.367954000
H	-4.392030000	-3.261338000	1.584284000
C	9.220904000	-0.659306000	-0.593012000
H	9.717954000	-0.527156000	0.373425000
H	9.863178000	-0.225986000	-1.366500000
H	9.148221000	-1.737003000	-0.774107000
C	4.525304000	1.168040000	3.299592000
H	5.252639000	0.415928000	3.622262000
H	3.957685000	0.758734000	2.456508000
H	3.824917000	1.333463000	4.125311000
C	-7.069571000	-4.006330000	1.789469000
H	-6.418498000	-4.876356000	1.653893000
H	-8.043499000	-4.239389000	1.347113000
H	-7.209603000	-3.868202000	2.866619000
C	3.489541000	6.842122000	-0.965346000
H	4.413343000	7.172141000	-0.478803000
H	3.534139000	7.139359000	-2.018474000
H	2.654338000	7.379427000	-0.503298000

Complex 7 (E = -4097.53175485; G = -4095.931442)

Au	4.143276000	0.821985000	-0.174092000
Au	-3.587340000	1.071964000	-0.108473000
N	-0.815037000	-4.065359000	0.209340000
N	6.835641000	0.011828000	-1.371203000
N	-6.486440000	1.248162000	-1.205230000
C	-0.816809000	-3.041418000	1.095279000
N	-0.563813000	-3.636729000	2.285729000
C	2.185294000	0.911793000	0.050344000
C	6.235565000	-0.871940000	-2.326731000
N	-5.849771000	3.008070000	-0.167843000
C	0.954118000	0.874366000	0.125639000
N	7.090827000	1.435961000	0.205458000
C	6.144719000	0.728365000	-0.453503000
C	5.667063000	-0.321280000	-3.483256000
C	-1.655751000	0.689604000	0.159223000
C	-0.571769000	-5.277156000	0.828062000

H	-0.536590000	-6.205600000	0.279499000
C	-5.455149000	1.752265000	-0.488013000
C	6.787340000	2.379107000	1.239870000
C	-1.071194000	-3.880729000	-1.187704000
C	-0.416109000	-5.005363000	2.147988000
H	-0.215517000	-5.647697000	2.991651000
C	6.256963000	-2.251476000	-2.063299000
C	-0.405982000	0.747998000	0.176144000
C	-2.404641000	-3.919504000	-1.622447000
C	-1.658383000	-2.533578000	4.162626000
C	-5.828155000	-0.294296000	-2.975823000
C	5.658255000	1.168336000	-3.761323000
H	6.142361000	1.677227000	-2.921981000
C	-5.020953000	3.930118000	0.550984000
C	-6.538427000	-0.058201000	-1.791257000
C	8.191489000	0.274149000	-1.292911000
H	8.903120000	-0.202308000	-1.949261000
C	-7.503448000	2.178359000	-1.338223000
H	-8.408011000	1.957274000	-1.883441000
C	-6.000232000	3.003576000	2.717090000
H	-6.699922000	2.554418000	2.004227000
C	-7.100872000	3.291281000	-0.677693000
H	-7.579053000	4.246262000	-0.524574000
C	5.100467000	-1.208547000	-4.403676000
H	4.651144000	-0.816655000	-5.311867000
C	-5.089064000	3.941091000	1.951061000
C	-4.151824000	4.748989000	-0.184428000
C	0.008790000	-3.633636000	-2.046152000
C	-3.317936000	5.606154000	0.536865000
H	-2.624717000	6.249568000	0.002072000
C	-4.972223000	0.760290000	-3.648151000
H	-4.988276000	1.662684000	-3.029465000
C	1.442635000	-3.596545000	-1.558405000
H	1.434577000	-3.725129000	-0.471269000
C	-1.595119000	-3.431919000	-3.854471000
H	-1.801765000	-3.249253000	-4.905557000
C	-0.473599000	-2.923608000	3.524652000
C	5.684285000	-3.094151000	-3.017024000
H	5.680759000	-4.166508000	-2.851081000
C	6.760828000	1.932216000	2.568586000
C	-3.358703000	5.639487000	1.926133000
H	-2.699759000	6.311278000	2.469262000
C	-3.546074000	-4.150223000	-0.653181000
H	-3.130623000	-4.598721000	0.255716000
C	-7.355673000	-1.024162000	-1.176881000
C	-3.032479000	-2.847570000	3.605916000
H	-2.906308000	-3.381128000	2.658588000
C	6.504436000	3.700556000	0.866674000
C	2.092838000	-2.244597000	-1.854136000
H	2.181899000	-2.068436000	-2.931302000

H	3.100427000	-2.203130000	-1.428438000
H	1.513847000	-1.423159000	-1.424012000
C	-4.178237000	-2.812489000	-0.263242000
H	-4.596724000	-2.309877000	-1.140273000
H	-3.437257000	-2.140543000	0.184986000
H	-4.984773000	-2.966216000	0.460054000
C	5.111662000	-2.578172000	-4.175591000
H	4.670222000	-3.252040000	-4.904751000
C	-5.181764000	1.868299000	3.337734000
H	-4.470923000	2.257809000	4.075561000
H	-4.609924000	1.333915000	2.570995000
H	-5.839026000	1.152178000	3.842862000
C	-0.283527000	-3.412914000	-3.395110000
H	0.529247000	-3.216477000	-4.088903000
C	0.803518000	-2.639724000	4.039147000
C	-5.540208000	1.130555000	-5.019482000
H	-4.939721000	1.925086000	-5.475302000
H	-6.572540000	1.487031000	-4.939990000
H	-5.531475000	0.271988000	-5.700146000
C	6.458344000	1.497275000	-5.022783000
H	7.491566000	1.143457000	-4.942920000
H	6.480525000	2.580209000	-5.185279000
H	6.011053000	1.035501000	-5.909798000
C	-1.539607000	-1.826414000	5.363359000
H	-2.439137000	-1.508689000	5.883295000
C	2.058614000	-3.020309000	3.278155000
H	1.850159000	-3.942619000	2.724248000
C	6.809397000	-2.797482000	-0.761346000
H	7.551775000	-2.085666000	-0.384631000
C	-4.235689000	4.817303000	2.625424000
H	-4.252565000	4.849617000	3.710956000
C	8.353153000	1.175482000	-0.292519000
H	9.234814000	1.651031000	0.108077000
C	4.227389000	1.700212000	-3.855270000
H	3.690810000	1.252795000	-4.699215000
H	4.234281000	2.785837000	-4.000208000
H	3.667159000	1.479701000	-2.939726000
C	6.522266000	4.155909000	-0.578791000
H	6.845707000	3.312940000	-1.198049000
C	-4.373646000	6.079454000	-2.306430000
H	-3.613499000	6.813661000	-2.018653000
H	-5.348801000	6.459835000	-1.984702000
H	-4.376982000	6.015787000	-3.399794000
C	-4.083339000	4.706909000	-1.697999000
H	-4.855638000	4.018400000	-2.055874000
C	-4.594578000	-5.117347000	-1.198992000
H	-5.356145000	-5.310082000	-0.435899000
H	-4.146539000	-6.075331000	-1.482559000
H	-5.107767000	-4.708164000	-2.074320000
C	-2.732804000	4.163119000	-2.168539000

H	-2.706257000	4.105884000	-3.262390000
H	-2.552078000	3.159942000	-1.765867000
H	-1.910809000	4.810660000	-1.843800000
C	6.138008000	4.185451000	3.213347000
H	5.879036000	4.897044000	3.992448000
C	6.425989000	2.867217000	3.550474000
H	6.385077000	2.557578000	4.590624000
C	-2.645614000	-3.682623000	-2.977024000
H	-3.666801000	-3.685798000	-3.345361000
C	-0.292754000	-1.530442000	5.896638000
H	-0.220229000	-0.980474000	6.830876000
C	-6.827216000	3.733383000	3.774133000
H	-6.195110000	4.155505000	4.562441000
H	-7.525198000	3.036872000	4.250487000
H	-7.408841000	4.549545000	3.333364000
C	-5.958754000	-1.559674000	-3.558002000
H	-5.427029000	-1.775413000	-4.480522000
C	2.252998000	-4.747688000	-2.156898000
H	1.804716000	-5.718525000	-1.920325000
H	3.274418000	-4.738904000	-1.762009000
H	2.315690000	-4.662369000	-3.247422000
C	-3.814496000	-3.761015000	4.551336000
H	-3.996340000	-3.274261000	5.515825000
H	-3.273084000	-4.693662000	4.741196000
H	-4.787070000	-4.014122000	4.115581000
C	7.027902000	0.485764000	2.932692000
H	7.467572000	-0.006035000	2.058547000
C	0.866960000	-1.933755000	5.240866000
H	1.833945000	-1.690870000	5.669241000
C	-7.456520000	-2.266541000	-1.802763000
H	-8.078579000	-3.039617000	-1.364046000
C	6.178962000	4.597878000	1.886486000
H	5.949782000	5.629536000	1.634363000
C	-3.516038000	0.305145000	-3.754501000
H	-3.420035000	-0.584041000	-4.386821000
H	-3.103603000	0.068260000	-2.767607000
H	-2.903182000	1.098210000	-4.196365000
C	2.389164000	-1.930754000	2.253748000
H	1.556508000	-1.756701000	1.566772000
H	3.264888000	-2.211965000	1.660690000
H	2.607386000	-0.982502000	2.756563000
C	5.686106000	-2.882991000	0.277478000
H	4.925418000	-3.606497000	-0.038004000
H	5.195283000	-1.913576000	0.411138000
H	6.082422000	-3.206271000	1.246540000
C	3.258644000	-3.293849000	4.180391000
H	3.616080000	-2.382526000	4.671271000
H	4.086991000	-3.685280000	3.580953000
H	3.024921000	-4.030164000	4.956514000
C	-3.806230000	-1.561508000	3.313267000

H	-4.790728000	-1.793561000	2.895579000
H	-3.270904000	-0.931475000	2.594294000
H	-3.957546000	-0.974341000	4.225215000
C	-6.770119000	-2.529665000	-2.985310000
H	-6.871050000	-3.500825000	-3.461841000
C	8.024721000	0.350660000	4.082770000
H	7.630070000	0.771061000	5.013743000
H	8.968289000	0.857700000	3.856086000
H	8.242528000	-0.706677000	4.266877000
C	-8.057490000	-0.737041000	0.137764000
H	-8.314398000	0.327735000	0.155772000
C	5.119541000	4.554564000	-1.041955000
H	5.134731000	4.837681000	-2.100153000
H	4.743298000	5.410381000	-0.470582000
H	4.412207000	3.727293000	-0.916987000
C	-7.107657000	-0.995230000	1.312592000
H	-7.578385000	-0.697115000	2.255891000
H	-6.171219000	-0.439897000	1.205294000
H	-6.862429000	-2.060906000	1.377765000
C	7.508960000	-4.144804000	-0.919318000
H	7.982447000	-4.429036000	0.025980000
H	8.285187000	-4.108875000	-1.690529000
H	6.805197000	-4.942047000	-1.181694000
C	5.712011000	-0.228893000	3.252143000
H	5.891670000	-1.289655000	3.457667000
H	5.010668000	-0.152808000	2.414394000
H	5.232862000	0.212655000	4.133644000
C	-9.355606000	-1.518770000	0.316415000
H	-9.171858000	-2.592082000	0.434216000
H	-10.033474000	-1.379770000	-0.531989000
H	-9.869660000	-1.177338000	1.220515000
Cu	-1.127615000	-1.203651000	0.683443000
C	7.522040000	5.292999000	-0.792976000
H	8.530581000	5.000554000	-0.482534000
H	7.558840000	5.569913000	-1.851966000
H	7.239905000	6.186190000	-0.225154000

TS 7 π,π (E = -4097.51891381; G = -4095.916562)

Au	3.906625000	0.809572000	-0.191270000
Au	-3.862499000	0.928185000	-0.168062000
N	-0.094203000	-4.015107000	0.386067000
N	6.652869000	0.530173000	-1.529102000
N	-6.829326000	0.584370000	-0.971408000
C	0.043380000	-2.923096000	1.175895000
N	0.222915000	-3.436270000	2.417994000
C	1.945666000	0.727671000	0.050696000
C	6.228074000	-0.479420000	-2.454514000
N	-6.396064000	2.527469000	-0.187667000
C	0.707557000	0.837463000	0.045505000

N	6.675954000	2.006974000	0.019892000
C	5.878704000	1.080438000	-0.563516000
C	5.477660000	-0.091394000	-3.572851000
C	-1.902346000	0.762844000	0.006449000
C	-0.005240000	-5.187208000	1.115397000
H	-0.098106000	-6.157772000	0.653208000
C	-5.830770000	1.321472000	-0.431649000
C	6.241575000	2.890728000	1.061432000
C	-0.433862000	-3.934944000	-1.002608000
C	0.191183000	-4.819893000	2.405583000
H	0.307529000	-5.403509000	3.305754000
C	6.625115000	-1.806238000	-2.218964000
C	-0.663222000	0.846422000	0.025802000
C	-1.794819000	-3.990361000	-1.342258000
C	-0.806344000	-2.069041000	4.151029000
C	-6.126179000	-1.018672000	-2.668151000
C	5.076945000	1.346703000	-3.833018000
H	5.438730000	1.959756000	-3.001699000
C	-5.669557000	3.643158000	0.341872000
C	-6.710704000	-0.771746000	-1.418649000
C	7.908962000	1.109583000	-1.554323000
H	8.663800000	0.800677000	-2.260552000
C	-7.996544000	1.321661000	-1.071524000
H	-8.900843000	0.901000000	-1.483059000
C	-6.260927000	2.821903000	2.684879000
H	-6.920211000	2.171415000	2.100629000
C	-7.722280000	2.551424000	-0.571636000
H	-8.335554000	3.431279000	-0.454914000
C	5.114618000	-1.092506000	-4.479225000
H	4.532697000	-0.826221000	-5.357111000
C	-5.606153000	3.802477000	1.733201000
C	-5.032083000	4.505755000	-0.561522000
C	0.585485000	-3.772880000	-1.950245000
C	-4.300359000	5.567235000	-0.024487000
H	-3.786995000	6.252129000	-0.693652000
C	-5.568972000	0.082552000	-3.547648000
H	-5.708046000	1.037846000	-3.032345000
C	2.049448000	-3.704700000	-1.566949000
H	2.120088000	-3.813142000	-0.479801000
C	-1.138082000	-3.694537000	-3.656441000
H	-1.415112000	-3.593075000	-4.702273000
C	0.352008000	-2.630877000	3.595527000
C	6.239864000	-2.766353000	-3.155646000
H	6.525501000	-3.803159000	-3.009668000
C	6.507316000	2.538252000	2.393431000
C	-4.216100000	5.752222000	1.350958000
H	-3.640137000	6.582816000	1.749548000
C	-2.877988000	-4.128399000	-0.291724000
H	-2.400851000	-4.385864000	0.658939000
C	-7.236101000	-1.787014000	-0.600286000

C	-2.187348000	-2.314648000	3.576460000
H	-2.068750000	-2.801180000	2.603861000
C	5.564510000	4.062856000	0.696495000
C	2.641474000	-2.344579000	-1.937356000
H	2.635622000	-2.191533000	-3.021319000
H	3.678858000	-2.269975000	-1.595354000
H	2.076181000	-1.528296000	-1.479497000
C	-3.595092000	-2.792207000	-0.094339000
H	-4.116993000	-2.484155000	-1.005866000
H	-2.887476000	-2.001075000	0.170159000
H	-4.334935000	-2.871937000	0.707533000
C	5.493015000	-2.412709000	-4.275525000
H	5.203410000	-3.175575000	-4.992931000
C	-5.196591000	1.935303000	3.336935000
H	-4.514479000	2.533483000	3.951816000
H	-4.599234000	1.419245000	2.577377000
H	-5.664067000	1.182621000	3.981033000
C	0.201902000	-3.655746000	-3.289426000
H	0.965594000	-3.526929000	-4.051264000
C	1.633433000	-2.442398000	4.139947000
C	-6.323233000	0.162140000	-4.876126000
H	-5.938292000	0.990960000	-5.479838000
H	-7.394276000	0.325577000	-4.717093000
H	-6.204619000	-0.758327000	-5.457980000
C	5.728995000	1.872840000	-5.112828000
H	6.820111000	1.792372000	-5.066673000
H	5.471742000	2.926837000	-5.263404000
H	5.386865000	1.315974000	-5.992048000
C	-0.651324000	-1.282488000	5.296905000
H	-1.527204000	-0.834189000	5.756943000
C	2.858293000	-3.026789000	3.464408000
H	2.574220000	-3.995270000	3.036306000
C	7.379847000	-2.188474000	-0.960936000
H	7.963162000	-1.318194000	-0.640162000
C	-4.862463000	4.879941000	2.220075000
H	-4.782927000	5.032512000	3.292608000
C	7.923218000	2.044429000	-0.573022000
H	8.692617000	2.724224000	-0.241554000
C	3.555546000	1.494285000	-3.882036000
H	3.125565000	0.919858000	-4.710022000
H	3.280228000	2.544842000	-4.024318000
H	3.097550000	1.145026000	-2.950131000
C	5.314353000	4.443715000	-0.748934000
H	5.723869000	3.654200000	-1.386962000
C	-5.627968000	5.531825000	-2.782210000
H	-4.957928000	6.389461000	-2.658866000
H	-6.615318000	5.817751000	-2.404846000
H	-5.718367000	5.333701000	-3.855532000
C	-5.092050000	4.294864000	-2.061156000
H	-5.784893000	3.471347000	-2.262140000

C	-3.859589000	-5.252191000	-0.620287000
H	-4.596341000	-5.354393000	0.183854000
H	-3.343196000	-6.211389000	-0.732661000
H	-4.410064000	-5.052283000	-1.544405000
C	-3.721797000	3.884539000	-2.605277000
H	-3.780946000	3.695288000	-3.682817000
H	-3.359658000	2.974324000	-2.113661000
H	-2.980870000	4.674846000	-2.441099000
C	5.361442000	4.569412000	3.058397000
H	5.011026000	5.229306000	3.847242000
C	6.048167000	3.404757000	3.387131000
H	6.226429000	3.165678000	4.430947000
C	-2.126733000	-3.860959000	-2.692180000
H	-3.171880000	-3.878656000	-2.987949000
C	0.602494000	-1.074039000	5.855452000
H	0.702053000	-0.459931000	6.746248000
C	-7.122193000	3.520919000	3.735608000
H	-6.520051000	4.148942000	4.400501000
H	-7.631581000	2.777446000	4.357885000
H	-7.884434000	4.154518000	3.270481000
C	-6.081822000	-2.347647000	-3.102579000
H	-5.641733000	-2.574952000	-4.069690000
C	2.846346000	-4.845481000	-2.201214000
H	2.436714000	-5.822515000	-1.923845000
H	3.889887000	-4.807429000	-1.870792000
H	2.842017000	-4.774627000	-3.294560000
C	-2.984967000	-3.262165000	4.475118000
H	-3.143964000	-2.823599000	5.466732000
H	-2.467637000	-4.218075000	4.608425000
H	-3.967610000	-3.465606000	4.034998000
C	7.206154000	1.239814000	2.744239000
H	7.776742000	0.917490000	1.866273000
C	1.733684000	-1.649327000	5.283530000
H	2.705905000	-1.476506000	5.733451000
C	-7.168343000	-3.094615000	-1.081612000
H	-7.564813000	-3.907583000	-0.482093000
C	5.125861000	4.896329000	1.728749000
H	4.594861000	5.811804000	1.482833000
C	-4.067226000	-0.104257000	-3.771149000
H	-3.856290000	-1.040617000	-4.299351000
H	-3.526840000	-0.121192000	-2.818119000
H	-3.667851000	0.718473000	-4.373993000
C	3.295512000	-2.119604000	2.311131000
H	2.500733000	-1.988800000	1.571525000
H	4.168413000	-2.541905000	1.805029000
H	3.562324000	-1.126945000	2.686693000
C	6.380568000	-2.508887000	0.154696000
H	5.779863000	-3.387122000	-0.108903000
H	5.697466000	-1.671584000	0.325883000
H	6.903391000	-2.723785000	1.093353000

C	4.024076000	-3.273486000	4.417215000
H	4.455405000	-2.336057000	4.784649000
H	4.820260000	-3.809549000	3.890544000
H	3.724992000	-3.874146000	5.282534000
C	-2.945907000	-1.009319000	3.340564000
H	-3.903369000	-1.214012000	2.851411000
H	-2.377953000	-0.330158000	2.697517000
H	-3.158964000	-0.489695000	4.280875000
C	-6.603727000	-3.371578000	-2.323565000
H	-6.571080000	-4.395842000	-2.684093000
C	8.192508000	1.383567000	3.900820000
H	7.684622000	1.598059000	4.846903000
H	8.918097000	2.182231000	3.715494000
H	8.744751000	0.447618000	4.034632000
C	-7.811869000	-1.475046000	0.768861000
H	-8.237063000	-0.466121000	0.731155000
C	3.818205000	4.544098000	-1.048896000
H	3.659464000	4.780691000	-2.106659000
H	3.344595000	5.333107000	-0.454468000
H	3.308430000	3.599933000	-0.828046000
C	-6.699170000	-1.462296000	1.822555000
H	-7.097176000	-1.148434000	2.793830000
H	-5.890052000	-0.779524000	1.545575000
H	-6.271148000	-2.464277000	1.940151000
C	8.357794000	-3.341799000	-1.164984000
H	8.952058000	-3.485873000	-0.256989000
H	9.045745000	-3.146526000	-1.993893000
H	7.840394000	-4.285762000	-1.366235000
C	6.163366000	0.158499000	3.038759000
H	6.646479000	-0.805447000	3.233078000
H	5.478712000	0.038251000	2.193806000
H	5.567714000	0.427067000	3.918930000
C	-8.933623000	-2.423403000	1.182375000
H	-8.563493000	-3.438632000	1.360562000
H	-9.720321000	-2.475860000	0.422836000
H	-9.385875000	-2.074717000	2.116286000
Cu	0.046327000	-1.099079000	0.645839000
C	6.039481000	5.743579000	-1.102676000
H	7.116005000	5.662526000	-0.919276000
H	5.891514000	5.984542000	-2.160828000
H	5.659660000	6.584134000	-0.511678000

Complex 7-fw (E = -4097.53067683; G = -4095.929826)

Au	3.342550000	1.221714000	0.037938000
Au	-4.290287000	0.521819000	-0.138092000
Cu	1.499599000	-1.540836000	0.136538000
N	-6.924150000	1.933376000	-0.614621000
N	5.356842000	3.242693000	0.977785000
N	6.097474000	2.292217000	-0.792100000

N	-7.286180000	-0.132680000	-0.201779000
N	2.444611000	-4.373282000	-0.083885000
N	0.519520000	-4.193724000	0.817261000
C	1.514643000	0.451413000	-0.005062000
C	0.271306000	0.323251000	-0.009130000
C	5.068180000	2.268598000	0.085157000
C	-2.326610000	0.358629000	-0.033021000
C	-6.290757000	0.776568000	-0.311625000
C	-8.521759000	0.442581000	-0.432874000
H	-9.435208000	-0.129758000	-0.388432000
C	7.014654000	3.272348000	-0.456790000
H	7.902978000	3.447366000	-1.043737000
C	-8.291450000	1.753728000	-0.694179000
H	-8.961966000	2.566641000	-0.925500000
C	1.526684000	-3.450857000	0.296741000
C	-1.093021000	0.323621000	-0.019231000
C	-7.065957000	-1.507259000	0.131457000
C	4.487143000	3.592238000	2.062200000
C	6.547348000	3.871495000	0.666829000
H	6.942067000	4.677640000	1.265453000
C	6.209457000	1.417847000	-1.921808000
C	-5.994429000	3.983075000	0.311440000
C	-6.242697000	3.178218000	-0.809287000
C	-1.814492000	-3.631106000	0.446383000
C	2.015842000	-5.661941000	0.187510000
H	2.614457000	-6.526181000	-0.056021000
C	-5.831096000	3.518414000	-2.105773000
C	-0.704884000	-3.636456000	1.305567000
C	-7.102995000	-1.874558000	1.484287000
C	7.098796000	0.332944000	-1.829989000
C	-6.061687000	2.603038000	-3.291301000
H	-6.708956000	1.782410000	-2.965614000
C	-7.406813000	-0.874436000	2.582724000
H	-7.712594000	0.064950000	2.111298000
C	5.440686000	1.687189000	-3.061680000
C	3.550536000	4.616279000	1.856761000
C	3.073229000	2.445908000	-3.453472000
H	2.991793000	1.948035000	-4.425892000
H	2.411401000	3.318573000	-3.468885000
H	2.707826000	1.755507000	-2.684987000
C	2.691120000	4.919342000	2.914814000
H	1.948013000	5.701689000	2.790299000
C	3.695795000	-4.091408000	-0.719262000
C	3.697879000	3.207860000	4.285080000
H	3.738270000	2.662553000	5.223834000
C	4.580794000	2.866437000	3.257807000
C	2.764986000	4.224601000	4.117133000
H	2.085493000	4.473764000	4.927433000
C	4.511083000	2.879963000	-3.164847000
H	4.510363000	3.397879000	-2.201024000

C	7.207610000	-0.497817000	-2.944582000
H	7.888124000	-1.341405000	-2.917293000
C	-5.149545000	4.726947000	-2.264465000
H	-4.809242000	5.020930000	-3.253182000
C	5.556998000	1.722254000	3.438946000
H	6.190885000	1.669789000	2.547867000
C	0.796501000	-5.546810000	0.765731000
H	0.105206000	-6.287783000	1.136355000
C	7.866892000	0.057839000	-0.550221000
H	8.146698000	1.023403000	-0.113771000
C	-6.809113000	-2.414229000	-0.906561000
C	-6.408312000	3.573636000	1.710619000
H	-6.988216000	2.647878000	1.637751000
C	5.013150000	3.869461000	-4.218458000
H	6.030149000	4.208276000	-3.994587000
H	4.361098000	4.748910000	-4.253050000
H	5.020091000	3.418004000	-5.216718000
C	-4.892917000	5.550977000	-1.174268000
H	-4.360112000	6.486822000	-1.318514000
C	3.703747000	-3.737792000	-2.075527000
C	-5.310117000	5.182158000	0.099550000
H	-5.096860000	5.829590000	0.945218000
C	6.473390000	1.942724000	4.642732000
H	5.906865000	1.969739000	5.579768000
H	7.026410000	2.883711000	4.555570000
H	7.200136000	1.126797000	4.717835000
C	3.419689000	5.337083000	0.529821000
H	4.257634000	5.035150000	-0.106810000
C	-6.771685000	3.321572000	-4.438523000
H	-7.725101000	3.749656000	-4.111918000
H	-6.975170000	2.619337000	-5.253878000
H	-6.158977000	4.132489000	-4.846211000
C	-0.735458000	-3.114887000	2.606420000
C	-1.722074000	-4.145878000	-0.976479000
H	-0.843839000	-4.795806000	-1.047971000
C	-6.163249000	-0.578787000	3.422869000
H	-5.813899000	-1.480519000	3.938684000
H	-6.390900000	0.174514000	4.184995000
H	-5.344802000	-0.199717000	2.801852000
C	-2.997573000	-3.074123000	0.932739000
H	-3.877608000	-3.039823000	0.298960000
C	-6.839108000	-3.211780000	1.790921000
H	-6.846732000	-3.532116000	2.828767000
C	-4.737619000	1.990081000	-3.754589000
H	-4.048961000	2.765693000	-4.108121000
H	-4.908422000	1.288529000	-4.578547000
H	-4.246564000	1.450163000	-2.937163000
C	5.578737000	0.814667000	-4.146477000
H	5.000722000	0.994862000	-5.048383000
C	6.453133000	-0.261595000	-4.090618000

H	6.553152000	-0.922264000	-4.947462000
C	4.868960000	-4.247198000	0.040580000
C	-6.561275000	-3.741566000	-0.547970000
H	-6.355214000	-4.473536000	-1.323516000
C	4.950420000	-3.551498000	-2.681915000
H	4.993968000	-3.267348000	-3.729189000
C	-6.568124000	-4.134310000	0.786264000
H	-6.361852000	-5.169231000	1.044641000
C	4.803980000	0.394012000	3.551434000
H	5.508915000	-0.439060000	3.642019000
H	4.175285000	0.221985000	2.670289000
H	4.156140000	0.385865000	4.435206000
C	2.434392000	-3.550582000	-2.881990000
H	1.586464000	-3.853824000	-2.260048000
C	-3.059067000	-2.544355000	2.216859000
H	-3.988697000	-2.111792000	2.571298000
C	-6.774937000	-1.984844000	-2.359465000
H	-7.123655000	-0.948704000	-2.413057000
C	0.482866000	-3.128873000	3.508278000
H	1.247257000	-3.752510000	3.032749000
C	-5.343697000	-2.015746000	-2.899279000
H	-4.678201000	-1.402112000	-2.281921000
H	-5.313407000	-1.630477000	-3.924362000
H	-4.949912000	-3.037844000	-2.912776000
C	-1.941427000	-2.559163000	3.042441000
H	-2.004080000	-2.135049000	4.040734000
C	-8.566467000	-1.343158000	3.462183000
H	-9.463993000	-1.541979000	2.867472000
H	-8.811521000	-0.573695000	4.201809000
H	-8.313361000	-2.258302000	4.008096000
C	2.129828000	4.916111000	-0.179974000
H	1.247909000	5.212447000	0.398984000
H	2.091248000	3.829524000	-0.318565000
H	2.063196000	5.391817000	-1.164742000
C	4.803567000	-4.503075000	1.534798000
H	3.935933000	-5.141627000	1.733797000
C	-5.175839000	3.280435000	2.569337000
H	-4.562379000	4.179488000	2.696062000
H	-4.550424000	2.507236000	2.109407000
H	-5.476665000	2.934404000	3.564163000
C	3.492926000	6.854787000	0.694505000
H	3.462895000	7.342277000	-0.285670000
H	4.417533000	7.157777000	1.196763000
H	2.649572000	7.237289000	1.279294000
C	-7.711897000	-2.828726000	-3.223368000
H	-7.399058000	-3.877881000	-3.251645000
H	-7.710814000	-2.456079000	-4.253148000
H	-8.740400000	-2.792726000	-2.849636000
C	6.970420000	-0.665057000	0.461665000
H	6.680221000	-1.646847000	0.076407000

H	6.052068000	-0.107797000	0.667555000
H	7.504643000	-0.810679000	1.407284000
C	6.126014000	-3.744307000	-1.970573000
H	7.084459000	-3.619608000	-2.465965000
C	6.085983000	-4.085146000	-0.621277000
H	7.015201000	-4.218113000	-0.077040000
C	6.031951000	-5.218746000	2.087680000
H	6.240548000	-6.146534000	1.544977000
H	5.868414000	-5.471227000	3.140160000
H	6.926227000	-4.587796000	2.041041000
C	-2.936921000	-4.979876000	-1.376130000
H	-3.852309000	-4.381171000	-1.386696000
H	-3.090254000	-5.818042000	-0.688089000
H	-2.797257000	-5.387065000	-2.383178000
C	2.421280000	-4.427036000	-4.134442000
H	3.215461000	-4.144606000	-4.833603000
H	1.465117000	-4.317729000	-4.657235000
H	2.552530000	-5.484391000	-3.882163000
C	-7.303444000	4.626157000	2.365232000
H	-7.630177000	4.282850000	3.352636000
H	-8.195459000	4.822915000	1.761389000
H	-6.772942000	5.574523000	2.502603000
C	2.240308000	-2.074142000	-3.236255000
H	2.187092000	-1.455416000	-2.332228000
H	1.310987000	-1.933454000	-3.798966000
H	3.068568000	-1.701117000	-3.849046000
C	-1.507934000	-2.977465000	-1.943160000
H	-1.395019000	-3.345059000	-2.969598000
H	-0.610246000	-2.407605000	-1.681067000
H	-2.358164000	-2.288104000	-1.916016000
C	4.578115000	-3.176102000	2.270433000
H	5.434238000	-2.509917000	2.122042000
H	4.460593000	-3.347798000	3.346118000
H	3.684286000	-2.659798000	1.906459000
C	1.063026000	-1.720217000	3.654742000
H	1.332707000	-1.301471000	2.677791000
H	1.962697000	-1.736838000	4.279852000
H	0.337200000	-1.043051000	4.118916000
C	0.175883000	-3.744176000	4.873551000
H	-0.540742000	-3.139225000	5.438849000
H	1.092280000	-3.811660000	5.469708000
H	-0.238638000	-4.752606000	4.772403000
C	9.154628000	-0.729440000	-0.772944000
H	9.715765000	-0.790536000	0.164883000
H	9.797547000	-0.256225000	-1.522283000
H	8.951342000	-1.755833000	-1.096959000

Complex 7'-*gem* (E = -4097.52583790; G = -4095.928369)

Au	-4.499121000	0.465291000	-0.041784000

N	-7.489324000	0.839399000	-0.486238000
N	4.129598000	4.025842000	1.101094000
N	5.202055000	3.400066000	-0.635377000
N	-7.017274000	-1.228393000	-0.209896000
N	3.919869000	-3.741992000	-0.268723000
N	1.931364000	-3.935711000	0.503404000
C	1.254490000	0.655488000	0.160222000
C	0.019104000	0.802499000	0.170636000
C	4.178502000	3.039490000	0.174309000
C	-2.568205000	0.818585000	0.109527000
C	-6.455399000	0.001475000	-0.246923000
C	-8.381220000	-1.163422000	-0.423916000
H	-9.000748000	-2.046700000	-0.430951000
C	5.776189000	4.591537000	-0.226449000
H	6.602414000	5.040079000	-0.756167000
C	-8.680790000	0.148321000	-0.598709000
H	-9.616446000	0.650361000	-0.790198000
C	2.860820000	-3.014399000	0.148821000
C	-1.334206000	0.875969000	0.149743000
C	-6.273908000	-2.437170000	-0.013982000
C	3.134246000	4.056096000	2.130475000
C	5.097023000	4.987070000	0.879074000
H	5.206976000	5.851361000	1.515623000
C	5.625883000	2.653141000	-1.781002000
C	-7.433234000	3.064395000	0.504464000
C	-7.333843000	2.255661000	-0.636201000
C	-0.395566000	-3.418447000	-0.002392000
C	3.655839000	-5.098809000	-0.182562000
H	4.384935000	-5.838082000	-0.475512000
C	-7.055931000	2.760053000	-1.914967000
C	0.617565000	-3.575092000	0.955803000
C	-6.110995000	-2.919858000	1.292587000
C	6.801709000	1.889153000	-1.674327000
C	-6.909946000	1.859600000	-3.125199000
H	-7.194574000	0.844332000	-2.830628000
C	-6.655237000	-2.180056000	2.497960000
H	-7.307842000	-1.378332000	2.137989000
C	4.867399000	2.730766000	-2.956117000
C	1.952523000	4.772572000	1.889225000
C	2.383688000	2.604656000	-3.219736000
H	2.463172000	2.012066000	-4.138140000
H	1.454879000	3.183197000	-3.269212000
H	2.304617000	1.910357000	-2.374456000
C	0.971253000	4.746100000	2.882520000
H	0.039511000	5.282027000	2.725272000
C	5.146602000	-3.209791000	-0.781423000
C	2.348620000	3.331113000	4.269208000
H	2.487248000	2.772095000	5.190254000
C	3.360386000	3.325517000	3.305874000
C	1.166869000	4.033280000	4.060319000

H	0.389711000	4.021029000	4.819502000
C	3.587328000	3.535673000	-3.056512000
H	3.452731000	4.085699000	-2.119918000
C	7.225455000	1.205811000	-2.813766000
H	8.132916000	0.613154000	-2.776856000
C	-6.875593000	4.139996000	-2.032991000
H	-6.651634000	4.567226000	-3.006407000
C	4.622735000	2.516168000	3.524121000
H	5.312417000	2.734048000	2.702100000
C	2.398273000	-5.219862000	0.309587000
H	1.798840000	-6.087632000	0.537258000
C	7.536840000	1.774616000	-0.351794000
H	7.490973000	2.751841000	0.142445000
C	-5.736113000	-3.076756000	-1.140089000
C	-7.687325000	2.487497000	1.882390000
H	-7.919375000	1.423841000	1.767616000
C	3.652541000	4.562232000	-4.187633000
H	4.503813000	5.239411000	-4.062389000
H	2.737784000	5.164383000	-4.201421000
H	3.746533000	4.078894000	-5.166000000
C	-6.969365000	4.969966000	-0.921444000
H	-6.823135000	6.040774000	-1.033484000
C	5.179122000	-2.758011000	-2.107504000
C	-7.244212000	4.437822000	0.333274000
H	-7.304746000	5.096374000	1.194888000
C	5.326601000	2.896056000	4.826771000
H	4.710857000	2.661479000	5.701488000
H	5.561278000	3.965160000	4.855520000
H	6.264674000	2.339116000	4.923657000
C	1.709769000	5.513799000	0.589578000
H	2.634525000	5.487236000	0.004290000
C	-7.837541000	2.282602000	-4.264399000
H	-8.882695000	2.308674000	-3.939179000
H	-7.757837000	1.575572000	-5.096969000
H	-7.577936000	3.275110000	-4.647942000
C	0.426232000	-3.323890000	2.322197000
C	-0.169922000	-3.681073000	-1.477834000
H	0.847410000	-4.065273000	-1.606141000
C	-5.511979000	-1.529158000	3.280593000
H	-4.831876000	-2.288673000	3.682573000
H	-5.906052000	-0.949524000	4.122636000
H	-4.930004000	-0.855008000	2.642498000
C	-1.641617000	-2.978155000	0.448727000
H	-2.447115000	-2.825826000	-0.263127000
C	-5.379767000	-4.099080000	1.454210000
H	-5.225282000	-4.496881000	2.452978000
C	-5.450745000	1.815147000	-3.584671000
H	-5.112192000	2.805706000	-3.908663000
H	-5.336545000	1.126064000	-4.428618000
H	-4.792326000	1.480428000	-2.774982000

C	5.328795000	2.014909000	-4.065985000
H	4.762604000	2.049968000	-4.992381000
C	6.498063000	1.270648000	-3.999423000
H	6.847003000	0.731815000	-4.876187000
C	6.272323000	-3.216897000	0.059473000
C	-5.020878000	-4.257514000	-0.925768000
H	-4.591001000	-4.780306000	-1.775588000
C	6.408796000	-2.308813000	-2.598358000
H	6.471289000	-1.947137000	-3.620369000
C	-4.843868000	-4.763711000	0.356851000
H	-4.276213000	-5.678419000	0.503021000
C	4.307668000	1.018659000	3.481778000
H	5.221879000	0.428750000	3.606684000
H	3.845545000	0.739409000	2.527684000
H	3.613315000	0.742221000	4.282968000
C	3.955378000	-2.745734000	-3.001607000
H	3.122088000	-3.191553000	-2.449769000
C	-1.860713000	-2.712626000	1.795343000
H	-2.834827000	-2.357975000	2.118542000
C	-5.885948000	-2.518414000	-2.540741000
H	-6.496935000	-1.612082000	-2.483827000
C	1.549362000	-3.451255000	3.331176000
H	2.398781000	-3.932788000	2.835433000
C	-4.523302000	-2.117452000	-3.110596000
H	-4.023878000	-1.390201000	-2.461332000
H	-4.642576000	-1.666092000	-4.101515000
H	-3.866571000	-2.988129000	-3.216743000
C	-0.839922000	-2.889448000	2.722225000
H	-1.021648000	-2.672829000	3.771031000
C	-7.494495000	-3.087338000	3.397144000
H	-8.313037000	-3.555200000	2.840543000
H	-7.928961000	-2.505791000	4.216978000
H	-6.889316000	-3.884071000	3.842532000
C	0.625395000	4.814851000	-0.234232000
H	-0.335610000	4.820024000	0.292183000
H	0.889536000	3.771162000	-0.432527000
H	0.487613000	5.323949000	-1.194603000
C	6.158719000	-3.617513000	1.517872000
H	5.346166000	-4.346822000	1.605501000
C	-6.426871000	2.594125000	2.744273000
H	-6.153271000	3.642068000	2.910486000
H	-5.577587000	2.095454000	2.263872000
H	-6.591026000	2.128038000	3.722059000
C	1.362896000	6.982845000	0.831422000
H	1.250343000	7.506187000	-0.124001000
H	2.146269000	7.488168000	1.405908000
H	0.420685000	7.088091000	1.379510000
C	-6.607447000	-3.503018000	-3.461448000
H	-6.037124000	-4.430470000	-3.581322000
H	-6.741411000	-3.063345000	-4.455553000

H	-7.595787000	-3.763657000	-3.068723000
C	6.828696000	0.761838000	0.556008000
H	6.864884000	-0.236440000	0.109943000
H	5.777740000	1.020457000	0.714550000
H	7.322186000	0.718372000	1.533287000
C	7.546606000	-2.331327000	-1.803195000
H	8.496464000	-1.993694000	-2.208525000
C	7.478535000	-2.775158000	-0.485562000
H	8.374650000	-2.769413000	0.126464000
C	7.420623000	-4.275658000	2.068409000
H	7.738341000	-5.122122000	1.450967000
H	7.231777000	-4.646703000	3.080862000
H	8.254584000	-3.568708000	2.133620000
C	-1.135017000	-4.746095000	-2.000718000
H	-2.174600000	-4.411459000	-1.918329000
H	-1.037754000	-5.682828000	-1.441640000
H	-0.933509000	-4.956377000	-3.056611000
C	4.173986000	-3.586511000	-4.260000000
H	4.973307000	-3.173502000	-4.884858000
H	3.259207000	-3.608383000	-4.861984000
H	4.440605000	-4.618410000	-4.008759000
C	-8.884880000	3.147172000	2.565935000
H	-9.083672000	2.661926000	3.527431000
H	-9.788281000	3.068259000	1.952386000
H	-8.702631000	4.208840000	2.764040000
C	3.560872000	-1.310319000	-3.356530000
H	3.369718000	-0.719970000	-2.453145000
H	2.650825000	-1.303124000	-3.966302000
H	4.353724000	-0.811113000	-3.922802000
C	-0.281104000	-2.388710000	-2.289443000
H	-0.083974000	-2.587416000	-3.348898000
H	0.436164000	-1.639465000	-1.937898000
H	-1.283787000	-1.955702000	-2.206993000
C	5.772529000	-2.394484000	2.358459000
H	6.575096000	-1.649507000	2.338166000
H	5.602464000	-2.684373000	3.401176000
H	4.861833000	-1.919139000	1.980784000
C	2.006274000	-2.064587000	3.792285000
H	2.296591000	-1.444832000	2.936679000
H	2.862968000	-2.146794000	4.470221000
H	1.200585000	-1.544334000	4.322684000
C	1.154602000	-4.327417000	4.519919000
H	0.336278000	-3.880420000	5.094099000
H	2.005998000	-4.448115000	5.198059000
H	0.835286000	-5.322989000	4.194469000
C	9.009887000	1.408989000	-0.503116000
H	9.505235000	1.466470000	0.471328000
H	9.530325000	2.086472000	-1.187885000
H	9.137149000	0.385315000	-0.871443000
Cu	2.885638000	1.633531000	0.109931000

Au	2.386735000	-1.053731000	0.158191000

TS 7' π,π (E = -4097.49420405; G = -4095.893828)

Au	0.313507000	-1.008613000	0.584625000
Au	-3.646907000	1.027506000	-0.230918000
N	0.374953000	-4.000747000	0.367552000
N	6.734529000	0.884396000	-1.564966000
N	-6.567030000	0.452531000	-1.071524000
C	0.450693000	-2.900937000	1.148809000
N	0.661231000	-3.371995000	2.398692000
C	2.181811000	1.110405000	-0.064359000
C	6.398827000	-0.161486000	-2.484360000
N	-6.314482000	2.387915000	-0.194391000
C	0.934352000	1.141979000	-0.039226000
N	6.629824000	2.362712000	-0.028418000
C	5.892061000	1.399138000	-0.633479000
C	5.579631000	0.142212000	-3.580492000
C	-1.680798000	1.003411000	-0.062304000
C	0.528882000	-5.152469000	1.118544000
H	0.493716000	-6.133254000	0.670706000
C	-5.641173000	1.250929000	-0.490068000
C	6.101948000	3.223334000	0.988354000
C	0.068947000	-3.948804000	-1.030903000
C	0.706152000	-4.755234000	2.403080000
H	0.861158000	-5.316752000	3.311214000
C	6.938281000	-1.441925000	-2.263077000
C	-0.440334000	1.099648000	-0.041303000
C	-1.281358000	-4.014444000	-1.403875000
C	-0.388869000	-2.013631000	4.124366000
C	-5.712585000	-1.032117000	-2.809008000
C	5.034207000	1.532536000	-3.839021000
H	5.316341000	2.174202000	-2.999112000
C	-5.700323000	3.529325000	0.416043000
C	-6.333889000	-0.873471000	-1.563312000
C	7.961573000	1.525485000	-1.551440000
H	8.754071000	1.258524000	-2.233335000
C	-7.796024000	1.085564000	-1.144742000
H	-8.657548000	0.604920000	-1.581741000
C	-6.192787000	2.483429000	2.694062000
H	-6.738475000	1.780383000	2.056369000
C	-7.636258000	2.309667000	-0.585286000
H	-8.328422000	3.122559000	-0.430257000
C	5.294616000	-0.893497000	-4.476718000
H	4.664174000	-0.690544000	-5.338168000
C	-5.640373000	3.586873000	1.815221000
C	-5.157843000	4.517368000	-0.418136000
C	1.113846000	-3.786157000	-1.950265000
C	-4.534623000	5.604068000	0.199337000
H	-4.097253000	6.386347000	-0.414713000

C	-5.228065000	0.137188000	-3.641744000
H	-5.445216000	1.060115000	-3.095624000
C	2.560748000	-3.654198000	-1.521658000
H	2.612085000	-3.820571000	-0.440474000
C	-0.562676000	-3.759757000	-3.703963000
H	-0.811046000	-3.681266000	-4.758908000
C	0.782083000	-2.524201000	3.547429000
C	6.626106000	-2.437784000	-3.189316000
H	7.023105000	-3.438759000	-3.056278000
C	6.164204000	2.805550000	2.327652000
C	-4.457543000	5.690695000	1.585012000
H	-3.964839000	6.542153000	2.046256000
C	-2.391546000	-4.116046000	-0.378799000
H	-1.938063000	-4.314474000	0.597534000
C	-6.796849000	-1.953829000	-0.790626000
C	-1.772544000	-2.372862000	3.620124000
H	-1.660990000	-2.956741000	2.701293000
C	5.520513000	4.435105000	0.588403000
C	3.060563000	-2.234943000	-1.799303000
H	3.052113000	-2.020209000	-2.872442000
H	4.087210000	-2.108199000	-1.440717000
H	2.429550000	-1.492759000	-1.300765000
C	-3.127257000	-2.778592000	-0.276920000
H	-3.627707000	-2.530400000	-1.217881000
H	-2.431176000	-1.968209000	-0.040881000
H	-3.886055000	-2.818658000	0.509649000
C	5.812473000	-2.166581000	-4.285955000
H	5.583410000	-2.956872000	-4.995493000
C	-5.048332000	1.712801000	3.356589000
H	-4.478623000	2.360948000	4.032115000
H	-4.355781000	1.319546000	2.604380000
H	-5.438823000	0.873110000	3.941657000
C	0.766542000	-3.697317000	-3.301091000
H	1.549569000	-3.565888000	-4.042676000
C	2.070379000	-2.220686000	4.017480000
C	-5.971332000	0.207328000	-4.977070000
H	-5.650209000	1.088726000	-5.542448000
H	-7.053976000	0.274824000	-4.827249000
H	-5.769934000	-0.675905000	-5.592947000
C	5.652201000	2.130916000	-5.104333000
H	6.744533000	2.165325000	-5.035365000
H	5.288067000	3.152695000	-5.256614000
H	5.388469000	1.544781000	-5.991633000
C	-0.240115000	-1.158604000	5.220320000
H	-1.124979000	-0.747415000	5.697658000
C	3.306468000	-2.760878000	3.325199000
H	3.039054000	-3.718141000	2.863766000
C	7.777700000	-1.738399000	-1.033815000
H	8.358596000	-0.840045000	-0.797893000
C	-5.003948000	4.692479000	2.383814000

H	-4.930528000	4.766014000	3.465145000
C	7.896236000	2.461222000	-0.573298000
H	8.617369000	3.184519000	-0.225773000
C	3.507206000	1.526762000	-3.915866000
H	3.150534000	0.919358000	-4.754818000
H	3.130922000	2.545280000	-4.058624000
H	3.064501000	1.128159000	-2.996678000
C	5.414544000	4.828783000	-0.871807000
H	6.087306000	4.183649000	-1.446377000
C	-5.834033000	5.643027000	-2.569068000
H	-5.243600000	6.545585000	-2.378277000
H	-6.845153000	5.813536000	-2.185040000
H	-5.899463000	5.513511000	-3.654564000
C	-5.195961000	4.410009000	-1.929398000
H	-5.813518000	3.545570000	-2.193875000
C	-3.347350000	-5.270025000	-0.677086000
H	-4.117468000	-5.331353000	0.099385000
H	-2.816836000	-6.227714000	-0.706417000
H	-3.857091000	-5.135434000	-1.635864000
C	-3.792326000	4.158611000	-2.485599000
H	-3.830296000	4.028509000	-3.572925000
H	-3.350527000	3.256710000	-2.046415000
H	-3.126583000	5.001414000	-2.268127000
C	5.043365000	4.872637000	2.923495000
H	4.624652000	5.521594000	3.687752000
C	5.624585000	3.662445000	3.288731000
H	5.651976000	3.377211000	4.335469000
C	-1.576893000	-3.914154000	-2.764824000
H	-2.613597000	-3.942161000	-3.088474000
C	1.019847000	-0.831540000	5.704515000
H	1.114332000	-0.162452000	6.555353000
C	-7.177698000	3.021401000	3.731907000
H	-6.690335000	3.708478000	4.431882000
H	-7.597642000	2.195990000	4.316499000
H	-8.005988000	3.556347000	3.255715000
C	-5.563320000	-2.337942000	-3.288455000
H	-5.090699000	-2.497150000	-4.253628000
C	3.450827000	-4.698804000	-2.194985000
H	3.100339000	-5.715652000	-1.989026000
H	4.479006000	-4.611994000	-1.828150000
H	3.475291000	-4.563170000	-3.281487000
C	-2.507844000	-3.247467000	4.637925000
H	-2.660650000	-2.711209000	5.581061000
H	-1.948268000	-4.162673000	4.858273000
H	-3.491781000	-3.534541000	4.251019000
C	6.812368000	1.491009000	2.715787000
H	6.676007000	0.796985000	1.878810000
C	2.163197000	-1.358600000	5.111008000
H	3.139403000	-1.092148000	5.503455000
C	-6.627819000	-3.234264000	-1.317139000

H	-6.973870000	-4.095250000	-0.754639000
C	4.988910000	5.253436000	1.588551000
H	4.520681000	6.194615000	1.315991000
C	-3.713864000	0.072751000	-3.850908000
H	-3.424235000	-0.828448000	-4.402957000
H	-3.182994000	0.069803000	-2.892299000
H	-3.373682000	0.942108000	-4.424131000
C	3.742689000	-1.818588000	2.198820000
H	2.936078000	-1.642114000	1.481227000
H	4.594479000	-2.246277000	1.662523000
H	4.044551000	-0.845244000	2.600273000
C	6.864508000	-2.016997000	0.163688000
H	6.272843000	-2.923250000	-0.009250000
H	6.172517000	-1.188412000	0.338494000
H	7.455198000	-2.165749000	1.074647000
C	4.465237000	-3.028767000	4.281995000
H	4.869405000	-2.102596000	4.703491000
H	5.281113000	-3.521109000	3.742691000
H	4.165214000	-3.677749000	5.111225000
C	-2.587981000	-1.126888000	3.274028000
H	-3.560206000	-1.415827000	2.861736000
H	-2.074808000	-0.511067000	2.528840000
H	-2.771865000	-0.508691000	4.159290000
C	-6.023321000	-3.424294000	-2.556838000
H	-5.911198000	-4.429773000	-2.952712000
C	8.318502000	1.674836000	2.927532000
H	8.509148000	2.371254000	3.751946000
H	8.809403000	2.068827000	2.032787000
H	8.788192000	0.716977000	3.176692000
C	-7.416003000	-1.734515000	0.577824000
H	-7.927516000	-0.766069000	0.562142000
C	3.992895000	4.582255000	-1.384036000
H	3.920992000	4.825885000	-2.449939000
H	3.270104000	5.203659000	-0.843150000
H	3.700576000	3.533854000	-1.248408000
C	-6.326224000	-1.657628000	1.652893000
H	-6.766958000	-1.403785000	2.623299000
H	-5.570986000	-0.903682000	1.410526000
H	-5.820146000	-2.624335000	1.754547000
C	8.769805000	-2.880087000	-1.231154000
H	9.423835000	-2.953110000	-0.356466000
H	9.399126000	-2.723038000	-2.113080000
H	8.264790000	-3.845877000	-1.339688000
C	6.176894000	0.852043000	3.948541000
H	6.568816000	-0.161712000	4.080060000
H	5.088119000	0.789604000	3.856148000
H	6.408351000	1.411339000	4.861608000
C	-8.458347000	-2.786854000	0.945272000
H	-8.006179000	-3.772183000	1.100955000
H	-9.227220000	-2.883256000	0.171817000

H	-8.951936000	-2.505704000	1.881055000
Cu	4.025951000	1.135176000	-0.292677000
C	5.847917000	6.273098000	-1.118289000
H	5.842845000	6.486557000	-2.192300000
H	5.170571000	6.987874000	-0.639228000
H	6.858509000	6.457787000	-0.739656000

Complex 7' (E = -4097.51399897; G = -4095.917180)

N	-7.011413000	2.240492000	-0.645652000
N	5.024482000	3.377159000	1.015925000
N	5.678925000	2.451529000	-0.800955000
N	-7.507612000	0.218539000	-0.184717000
N	2.012197000	-4.460011000	-0.160437000
N	0.080533000	-4.277763000	0.746253000
C	1.120634000	0.606292000	0.019340000
C	-0.131744000	0.509987000	0.002529000
C	4.667984000	2.451494000	0.097412000
C	-2.730483000	0.540204000	-0.065232000
C	-6.448076000	1.048871000	-0.332869000
C	-8.706404000	0.873642000	-0.399530000
H	-9.657278000	0.369511000	-0.322510000
C	6.653633000	3.369234000	-0.453016000
H	7.537742000	3.518915000	-1.053139000
C	-8.390299000	2.159964000	-0.694029000
H	-9.007806000	3.013051000	-0.929263000
C	1.096485000	-3.546477000	0.231584000
C	-1.493004000	0.514786000	-0.027061000
C	-7.373131000	-1.161619000	0.169246000
C	4.198117000	3.727365000	2.133429000
C	6.241166000	3.952260000	0.700447000
H	6.690839000	4.714773000	1.317191000
C	5.702305000	1.609671000	-1.960136000
C	-5.916734000	4.242412000	0.205068000
C	-6.237129000	3.420840000	-0.886157000
C	-2.228287000	-3.603961000	0.383642000
C	1.571723000	-5.747187000	0.099193000
H	2.167049000	-6.611895000	-0.150022000
C	-5.800151000	3.678724000	-2.192671000
C	-1.131370000	-3.704023000	1.252480000
C	-7.409509000	-1.508431000	1.527148000
C	6.498092000	0.452439000	-1.919841000
C	-6.117576000	2.751046000	-3.347725000
H	-6.811263000	1.984911000	-2.987130000
C	-7.630201000	-0.480099000	2.618877000
H	-7.763028000	0.496683000	2.143715000
C	4.920545000	1.967919000	-3.066345000
C	3.320649000	4.813080000	1.992991000
C	2.618121000	2.946940000	-3.347922000
H	2.453630000	2.467795000	-4.319258000

H	2.040476000	3.877379000	-3.332641000
H	2.221337000	2.285280000	-2.569827000
C	2.497804000	5.116388000	3.079549000
H	1.799818000	5.945138000	3.005286000
C	3.271118000	-4.171723000	-0.780195000
C	3.423234000	3.287085000	4.351048000
H	3.446909000	2.698161000	5.263660000
C	4.268307000	2.942852000	3.293083000
C	2.549514000	4.362984000	4.247257000
H	1.898914000	4.613274000	5.080640000
C	4.100352000	3.241547000	-3.113348000
H	4.186360000	3.739303000	-2.142642000
C	6.487825000	-0.367001000	-3.048603000
H	7.086524000	-1.271334000	-3.061147000
C	-5.011818000	4.814283000	-2.394400000
H	-4.649217000	5.040118000	-3.393185000
C	5.185554000	1.742487000	3.410150000
H	5.797566000	1.690743000	2.503963000
C	0.352017000	-5.630578000	0.677766000
H	-0.343658000	-6.371312000	1.040123000
C	7.291832000	0.092920000	-0.677531000
H	7.642011000	1.026465000	-0.222267000
C	-7.187263000	-2.099315000	-0.858007000
C	-6.356536000	3.905628000	1.615565000
H	-7.130033000	3.133049000	1.552192000
C	4.649694000	4.199116000	-4.172993000
H	5.702260000	4.437658000	-3.987495000
H	4.081991000	5.135857000	-4.167994000
H	4.573533000	3.766390000	-5.176622000
C	-4.676328000	5.647313000	-1.333498000
H	-4.058180000	6.523422000	-1.509164000
C	3.295828000	-3.853046000	-2.144423000
C	-5.123342000	5.363336000	-0.047505000
H	-4.846222000	6.016743000	0.774643000
C	6.136656000	1.873480000	4.600221000
H	5.591791000	1.888757000	5.550277000
H	6.730430000	2.791264000	4.536974000
H	6.826372000	1.023073000	4.626818000
C	3.205960000	5.593230000	0.698460000
H	4.042919000	5.308142000	0.052748000
C	-6.803796000	3.489329000	-4.497157000
H	-7.720459000	3.984935000	-4.161120000
H	-7.069447000	2.785039000	-5.292691000
H	-6.149027000	4.250665000	-4.934360000
C	-1.154057000	-3.246034000	2.576996000
C	-2.148928000	-4.053348000	-1.061524000
H	-1.266408000	-4.692252000	-1.171403000
C	-6.416574000	-0.376702000	3.543837000
H	-6.231670000	-1.323390000	4.063965000
H	-6.584958000	0.392764000	4.305082000

H	-5.512480000	-0.110569000	2.986820000
C	-3.391517000	-3.019684000	0.887715000
H	-4.261845000	-2.915717000	0.247329000
C	-7.235481000	-2.857368000	1.849209000
H	-7.251387000	-3.162017000	2.892002000
C	-4.847972000	2.040217000	-3.822550000
H	-4.116557000	2.759358000	-4.207867000
H	-5.081914000	1.333564000	-4.626181000
H	-4.374803000	1.486942000	-3.003882000
C	4.940441000	1.107773000	-4.168977000
H	4.348408000	1.354537000	-5.045833000
C	5.713648000	-0.045005000	-4.160078000
H	5.719274000	-0.699024000	-5.027900000
C	4.430893000	-4.285014000	0.005790000
C	-7.018790000	-3.434628000	-0.484284000
H	-6.866625000	-4.189093000	-1.250427000
C	4.549963000	-3.666063000	-2.734325000
H	4.607412000	-3.418573000	-3.790278000
C	-7.038663000	-3.809220000	0.855542000
H	-6.898833000	-4.852060000	1.126281000
C	4.370026000	0.449332000	3.492119000
H	5.035530000	-0.418847000	3.542512000
H	3.720035000	0.336100000	2.616655000
H	3.736356000	0.440625000	4.386172000
C	2.038194000	-3.709226000	-2.977279000
H	1.177784000	-3.943298000	-2.342833000
C	-3.448482000	-2.561461000	2.199302000
H	-4.364713000	-2.113421000	2.570070000
C	-7.123081000	-1.682293000	-2.313306000
H	-7.515315000	-0.662795000	-2.387544000
C	0.051554000	-3.337879000	3.490635000
H	0.839900000	-3.882826000	2.961274000
C	-5.669015000	-1.653416000	-2.790560000
H	-5.055247000	-1.010663000	-2.148065000
H	-5.606871000	-1.272538000	-3.815711000
H	-5.235291000	-2.659146000	-2.777712000
C	-2.342618000	-2.670785000	3.033362000
H	-2.397009000	-2.303150000	4.054364000
C	-8.903018000	-0.785404000	3.410434000
H	-9.777663000	-0.837186000	2.753792000
H	-9.082512000	-0.003276000	4.155799000
H	-8.822794000	-1.740271000	3.941467000
C	1.913070000	5.215620000	-0.030714000
H	1.034429000	5.500508000	0.559049000
H	1.860624000	4.135659000	-0.210159000
H	1.854457000	5.730045000	-0.996290000
C	4.344979000	-4.512628000	1.502868000
H	3.450180000	-5.111374000	1.704690000
C	-5.183336000	3.321277000	2.407646000
H	-4.381678000	4.060181000	2.519844000

H	-4.761554000	2.444870000	1.901716000
H	-5.508109000	3.017728000	3.408953000
C	3.295575000	7.102399000	0.921079000
H	3.280393000	7.624956000	-0.041181000
H	4.219258000	7.375629000	1.441674000
H	2.451545000	7.475489000	1.510890000
C	-7.981219000	-2.569817000	-3.213062000
H	-7.610831000	-3.600065000	-3.239847000
H	-7.963837000	-2.188823000	-4.239514000
H	-9.022770000	-2.592930000	-2.876518000
C	6.391083000	-0.610125000	0.343871000
H	6.020787000	-1.555253000	-0.064059000
H	5.522410000	-0.001026000	0.610262000
H	6.953450000	-0.828240000	1.258906000
C	5.715550000	-3.810106000	-1.994608000
H	6.681113000	-3.682297000	-2.475816000
C	5.656739000	-4.109555000	-0.636302000
H	6.577262000	-4.202041000	-0.069191000
C	5.538890000	-5.272868000	2.072395000
H	5.708761000	-6.216672000	1.544048000
H	5.360891000	-5.502119000	3.127896000
H	6.459740000	-4.682132000	2.020129000
C	-3.363387000	-4.881821000	-1.476395000
H	-4.287552000	-4.297616000	-1.427342000
H	-3.486099000	-5.759436000	-0.832951000
H	-3.246293000	-5.229899000	-2.508122000
C	2.027057000	-4.691627000	-4.149074000
H	2.843879000	-4.488592000	-4.850044000
H	1.085275000	-4.607382000	-4.701774000
H	2.127526000	-5.725659000	-3.803020000
C	-6.967789000	5.105189000	2.337883000
H	-7.341539000	4.799153000	3.320692000
H	-7.804616000	5.528198000	1.772654000
H	-6.232620000	5.900590000	2.500135000
C	1.873126000	-2.266331000	-3.459578000
H	1.836784000	-1.571144000	-2.612890000
H	0.943149000	-2.159561000	-4.028726000
H	2.703250000	-1.966673000	-4.108881000
C	-1.956989000	-2.843627000	-1.980877000
H	-1.853289000	-3.168505000	-3.022551000
H	-1.061374000	-2.276440000	-1.704087000
H	-2.812676000	-2.163881000	-1.917909000
C	4.172395000	-3.165794000	2.216289000
H	5.062502000	-2.544471000	2.073280000
H	4.029874000	-3.316796000	3.292067000
H	3.312035000	-2.610219000	1.829143000
C	0.591294000	-1.942562000	3.814758000
H	0.846142000	-1.396889000	2.899042000
H	1.491834000	-2.014237000	4.434613000
H	-0.150905000	-1.351723000	4.363242000

C	-0.271669000	-4.115654000	4.766607000
H	-1.036831000	-3.605137000	5.361345000
H	0.624529000	-4.211053000	5.388997000
H	-0.637161000	-5.122342000	4.538649000
C	8.524149000	-0.756404000	-0.972523000
H	9.117466000	-0.873498000	-0.060101000
H	9.162032000	-0.298049000	-1.735279000
H	8.252589000	-1.761161000	-1.313271000
Au	1.074978000	-1.544583000	0.099623000
Au	2.928092000	1.433338000	0.056421000
Cu	-4.573706000	0.712176000	-0.175800000

10. References

[1] F. Lazreg, S. Guidone, A. Gómez-Herrera, F. Nahra, C. S. J. Cazin. *Dalton Trans.*, **2017**, *46*, 2439.

[2] L-J. Cheng, C. J. Cordier. *Angew. Chem. Int. Ed.* **2015**, *54*, 13734.

[3] J. S. Siegel, F. A. L. Anet. *J. Org. Chem.,* **1988**, *53*, 2629.

[4] J. Cayuela-Castillo, F. J. Fernández-de-Córdova, M. S. See, I. Fernández, P. Ríos. *Chem. Sci.,* **2025**, *16*, 4684.

[5] T. J. Robilotto, J. Bacsa, T. G. Gray, J. P. Sadighi. *Angew. Chem. Int. Ed.,* **2012**, *51*, 12077.

[6] a) J. Sändstrom in *Dynamic NMR Spectroscopy*, Academic Press: London, **1982**, 77-92. b) F. P. Gasparro, N. H. Kolodny. *J. Chem. Educ.,* **1977**, *54*, 258.

[7] M. T. Huggins, T. Kesharwani, J. Buttrick, C. Nicholson. *J. Chem. Educ.,* **2020**, *97*, 1425.

[8] G. M. Sheldrick. *Acta Cryst*. **2008**, *A64*, 112.

[9] O. V. Dolomanov, L. J. Bourhis, R. J. Gildea, J. A. K. Howard, J.A.K., H. Puschmann. *J. Appl. Cryst*. **2009**, *42*, 339.

[10] P. van der Sluis, A. L. Spek. *Acta Cryst.* **1990**, *A46*, 194.

[11] C. Adamo, V. Barone. *J. Chem. Phys.,* **1999**, *110*, 6158.

[12] a) S. Grimme, J. Antony, S. Ehrlich, H. Krieg. *J. Chem. Phys.* **2010**, *132*, 154104. b) S. Grimme, S. Ehrlich, L. Goerigk. *J. Comput. Chem.* **2011**, *32*, 1456

[13] *Gaussian 09*, Revision E.01; M. J. Frisch; G. W. Trucks; H. B. Schlegel; G. E. Scuseria; M. A. Robb; J. R. Cheeseman; G. Scalmani; V. Barone; B. Mennucci; G. A. Petersson; H. Nakatsuji; M. Caricato; X. Li; H. P. Hratchian; A. F. Izmaylov; J. Bloino; G. Zheng; J. L. Sonnenberg; M. Hada; M. Ehara; K. Toyota; R. Fukuda; J. Hasegawa; M. Ishida; T. Nakajima; Y. Honda; O. Kitao; H. Nakai; T. Vreven; J. A. Montgomery, Jr.; J. E. Peralta; F. Ogliaro; M. Bearpark; J. J. Heyd; E. Brothers; K. N. Kudin; V. N. Staroverov; T. Keith; R. Kobayashi; J. Normand; K. Raghavachari; A. Rendell; J. C. Burant; S. S. Iyengar; J. Tomasi; M. Cossi; N. Rega; J. M. Millam; M. Klene; J. E. Knox; J. B. Cross; V. Bakken; C. Adamo; J. Jaramillo; R. Gomperts; R. E. Stratmann; O. Yazyev; A. J. Austin; R. Cammi; C. Pomelli; J. W. Ochterski; R. L. Martin; K. Morokuma; V. G. Zakrzewski; G. A. Voth; P. Salvador; J. J. Dannenberg; S. Dapprich; A. D. Daniels; O. Farkas; J. B. Foresman; J. V. Ortiz; J. Cioslowski; D. J. Fox, Gaussian, Inc.: Wallingford, CT, **2013**.

[14] A. V. Marenich, C. J. Cramer, D. G. Truhlar. *J. Phys. Chem. B,* **2009**, *113*, 6378.
[15] a) W. J. Hehre, R. Ditchfield, J. A. Pople. *J. Chem. Phys.,* **1972**, *56*, 2257. b) P. C. Hariharan, J. A. Pople. *Theor. Chim. Acta,* **1973**, *28*, 213.
[16] a) M. Dolg, U. Wedig, H. Stoll, H. Preuss. *J. Chem. Phys.,* **1987**, *86*, 866. b) D. Andrae, U. Häussermann, M. Dolg, H. Stoll, H. Preuss. *Theor. Chim. Acta,* **1990**, *77*, 123.
[17] A. W. Ehlers, M. Böhme, S. Dapprich, A. Gobbi, A. Höllwarth, V. Jonas, K. F. Köhler, R. Stegmann, A. Veldkamp, G. Frenking. *Chem. Phys. Lett.,* **1993**, *208*, 111.
[18] a) F. Weigend, R. Ahlrichs, *Phys. Chem. Chem. Phys.,* **2005**, *7*, 3297. b) F. Weigend. *Phys. Chem. Chem. Phys.,* **2006**, *8*, 1057.
[19] S. Grimme. *Chem. Eur. J.,* **2012**, *18*, 9955.
[20] G. Luchini, J. V. Alegre-Requena, I. Funes-Ardoiz, R. S. Paton. *F1000Research*, **2020**, *9*, 291
[21] Chemcraft - graphical software for visualization of quantum chemistry computations. https://www.chemcraftprog.com
[22] For reviews on the EDA method, see: a) F. M. Bickelhaupt, E. J. Baerends, in *Reviews in Computational Chemistry*, (Eds. K. B. Lipkowitz, D. B. Boyd), Wiley-VCH: New York, 2000, Vol. 15, pp. 1–86; b) M. von Hopffgarten, G. Frenking, *WIREs Comput. Mol. Sci.* **2012**, *2*, 43–62; c) I. Fernández, in *Applied Theoretical Organic Chemistry*, (Ed. D. J. Tantillo), World Scientific, New Jersey, 2018, pp. 191–226.
[23] NM. P. Mitoraj, A. Michalak and T. Ziegler, *J. Chem. Theory Comput.* **2009**,*5*, 962–975.
[24] a) G. te Velde, F. M. Bickelhaupt, E. J. Baerends, C. Fonseca Guerra, S. J. A. van Gisbergen, J. G. Snijders, T. Ziegler, *J. Comput. Chem*. **2001**, *22*, 931−967; b) *ADF2021*, SCM, Theoretical Chemistry, Vrije Universiteit, Amsterdam, The Netherlands, http://www.scm.com.
[25] J. G. Snijders, P. Vernooijs, E. J. Baerends, *At. Data Nucl. Data Tables* **1981**, *26*, 483−574.
[26] J. Krijn, E. J. Baerends, *Fit Functions in the HFS-Method*, Internal Report (in Dutch), Vrije Universiteit Amsterdam, The Netherlands, 1984.
[27] a) E. van Lenthe, E. J. Baerends, J. G. Snijders, J. Chem. Phys. 1993, 99, 4597−4610; b) E. van Lenthe, E. J. Baerends, J. G. Snijders, J. Chem. Phys. 1994, 101, 9783−9792; c) E. van Lenthe, A. Ehlers, E. J. Baerends, J. Chem. Phys. 1999, 110, 8943−8953.
